# Exceptionally large “through-space” nuclear spin coupling in a 2,4,6-tri(phosphanyl)–1,3,5-triphosphabenzene

**DOI:** 10.1039/d5sc07729j

**Published:** 2025-11-04

**Authors:** David C. Meier, Álvaro García-Romero, Daniel González-Pinardo, Nicholas H. Rees, Alex Lovstedt, Israel Fernández, Jose M. Goicoechea

**Affiliations:** a Department of Chemistry, Indiana University 800 East Kirkwood Ave. Bloomington Indiana 47405 USA jgoicoec@iu.edu; b Departamento de Química Orgánica and Centro de Innovación en Química Avanzada (ORFEO-CINQA), Universidad Complutense de Madrid, Facultad de Ciencias Químicas 28040 Madrid Spain israel@quim.ucm.es; c Department of Chemistry, University of Oxford, Chemistry Research Laboratory 12 Mansfield Rd. Oxford OX1 3TA UK

## Abstract

We describe the synthesis of a phosphanyl-functionalized 1,3,5-triphosphabenzene that exhibits remarkably large indirect spin–spin coupling (432 Hz) between phosphorus-31 nuclei. The magnitude of this coupling interaction is indicative of highly effective orbital overlap between two lone-pair type orbitals, each of which possesses significant s-orbital character. This was probed computationally using density functional theory calculations in order to deconvolute individual transmission pathways by dividing these into “through-space” and “through-bond” type interactions. The “through-space” transmission pathway can be chemically disrupted by oxidation of the pendant phosphanyl groups using either elemental sulfur or selenium. By engaging the phosphanyl s-orbitals in bonds to the chalcogen elements, the indirect interaction is perturbed, and the NMR spectra of the resulting compounds exhibit conventional “through-bond” coupling.

## Abstract 2

We describe the synthesis of a phosphanyl-functionalized 1,3,5-triphosphabenzene that exhibits remarkably large indirect spin–spin coupling (432 Hz) between phosphorus-31 nuclei.

## Graphical abstract

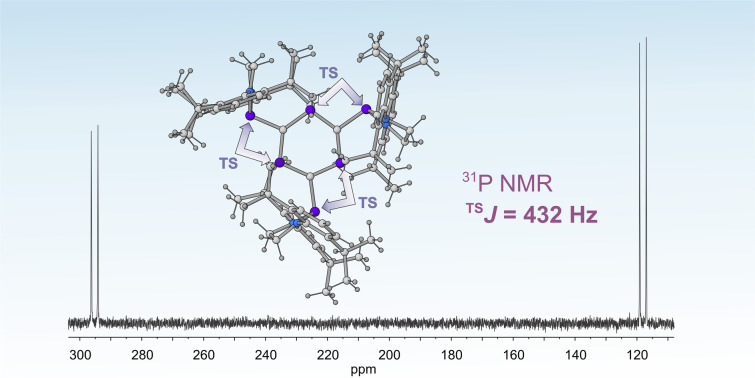

## Introduction

**Fig. 1:**
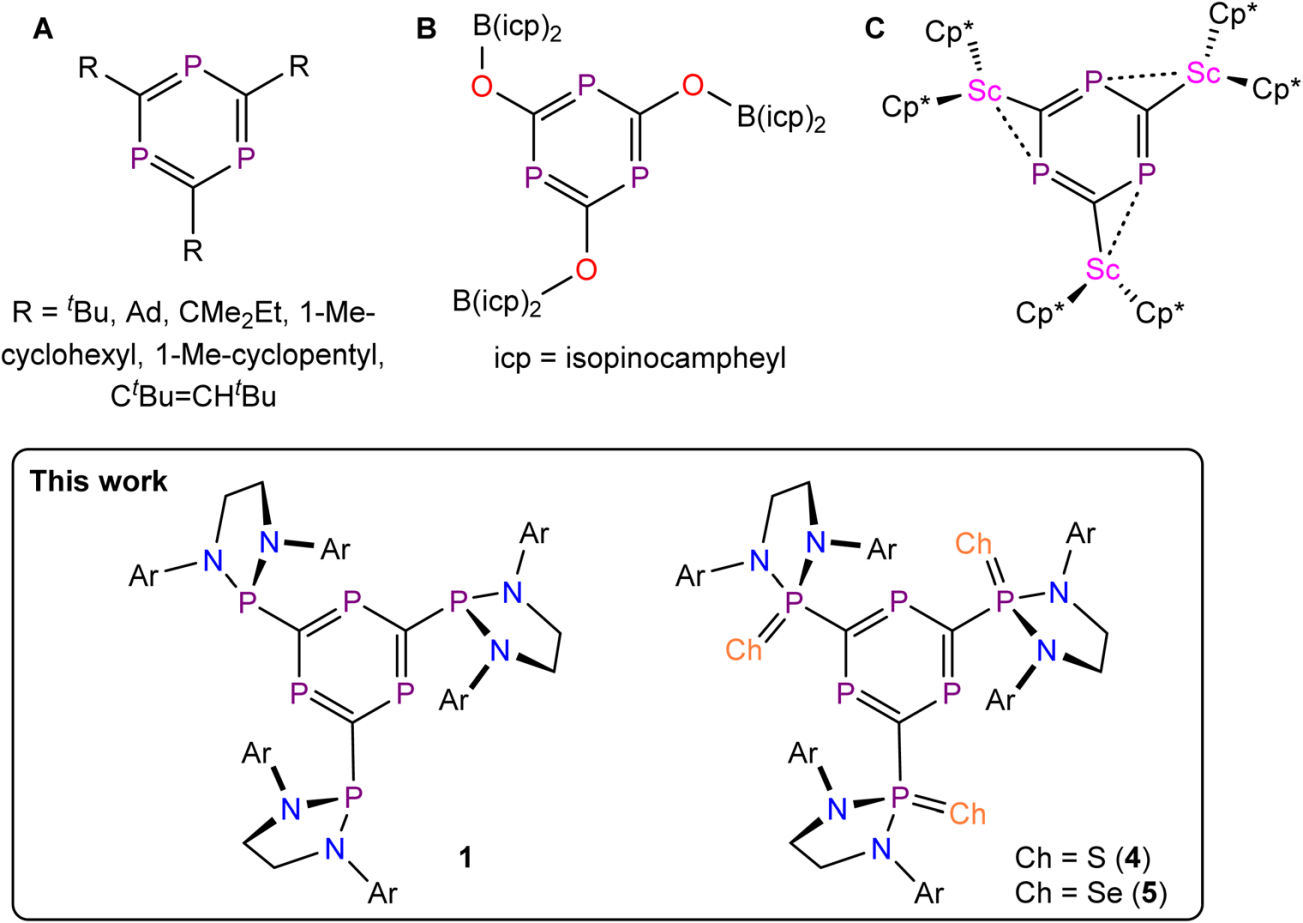
1,3,5-Triphosphabenzenes.

1,3,5-Triphosphabenzenes (or triphosphinines) are benzene isosteres in which alternating C–H units of a benzene ring have been replaced by (isolobal and valence isoelectronic) phosphorus atoms. They were first synthesized by the transition-metal mediated cyclotrimerization of kinetically stabilized phosphaalkynes (P≡C–R).^1–6^ Unfortunately, this methodology suffers from several limitations which prevent its widespread use. As a result, for over thirty years, the only accessible triphosphabenzenes featured sterically demanding carbon-based substituents associated with the central ring (*e.g.*^*t*^Bu, Ad; Fig. 1A). The planar, benzene-like structure of these compounds was confirmed by single-crystal X-ray diffraction shortly after their isolation.^7–9^ The coordination chemistry and reactivity of 1,3,5-triphosphabenzenes have been thoroughly reviewed by Falconer and Russell; the reader is referred to this publication for further information.^10^ In addition to its fundamental interest, 2,4,6-tri-*tert*-butyl-1,3,5-triphospha-benzene (^*t*^Bu_3_C_3_P_3_) has also been shown to be a viable platform for the activation of dihydrogen.^11^

More recently, Grützmacher and co-workers demonstrated that sodium phosphaethynolate, Na(PCO),^12^ can be functionalized with a commercially available chloro-borane, (–)-B-chlorodiisopinocampheylborane, to afford a boryloxy-functionalized phosphaalkyne that trimerizes at room temperature yielding a 1,3,5-triphosphabenzene (Fig. 1B).^13^ This species can be used to access 2,4,6-oxyfunctionalized phosphabenzenes and their coordination compounds.^14^ The cyclotrimerization of Grützmacher's boryloxy-functionalized phosphaalkyne proceeds spontaneously and in the absence of a metal catalyst. This suggests that the inductive capabilities of phosphaalkyne substituents strongly influence their reactivity. This hypothesis is further supported by the recent observation that metal cyaphido-complexes can also undergo a similar oligomerization reaction.^15^ For example, the reaction of the cyaphide-transfer reagent Mg(^Dipp^NacNac)(dioxane)(CP) (^Dipp^NacNac = CH{C(CH_3_)N(Dipp)}_2_)^16^ with Cp*_2_ScCl (Cp* = pentamethylcyclopentadienyl) is believed to give rise to an unobserved transient scandium cyaphido complex, which spontaneously cyclotrimerizes to give a trimetallated 1,3,5-triphosphabenzene (Fig. 1C).^17^

These observations prompted us to further explore the use of cyaphide transfer reagents for the synthesis of novel 1,3,5-triphosphabenzenes to better understand the electronic properties and reactivity of these heavier benzene analogues. Specifically, we were interested in appending a spectroscopic probe onto the triphosphabenzene ring that would allow us to experimentally measure the electronic properties of the ring-based phosphorus atoms. While these atoms are capable of coordinating to metal centers, they have also been shown to be extremely weak Brønsted bases. For example, 2,4,6-tri-*tert*-butyl-1,3,5-triphosphabenzene has a basicity somewhere between that of mesitylene and toluene.^18^ This is attributed to the high s-orbital character of the phosphorus atom lone pairs in such compounds.^19^ Using phosphanyl functional groups as an intramolecular probe, we can experimentally address the s-orbital character of the phosphorus lone-pairs in the triphosphabenzene ring. This approach gives rise to a very large “through-space” nuclear spin coupling between the two distinct types of phosphorus nuclei (*I* = ½) of the title compound.

## Results and discussion

In an effort to generate a phosphanyl-phosphaalkyne, we reacted the known bis(aryl-amino)-chlorophosphine [(H_2_C)_2_(NDipp)_2_]PCl (Dipp = 2,6-diisopropylphenyl)^20^ with [Mg(^Dipp^NacNac)(CP)]_2_ ({[Mg]CP}_2_).^21^ Bertrand and co-workers have previously shown that this phosphanyl-chloride can be used to access stable phosphanyl-phosphaketenes.^22^ However, upon reaction of [(H_2_C)_2_(NDipp)_2_]PCl with half of an equivalent of {[Mg]CP}_2_ we observed no evidence for the formation of a phosphanyl-phosphaalkyne, [(H_2_C)_2_(NDipp)_2_]P(CP). Rather, a mixture of products was observed in the *in situ*^31^P{^1^H} NMR spectra (Fig. S18). Over time, the reaction mixture gave rise to one major product, 1, and a minor impurity, 2. From this mixture, red needle-like crystals of a triphosphabenzene {[(H_2_C)_2_(NDipp)_2_]P}_3_C_3_P_3_ (1) were isolated.

**Fig. 2:**
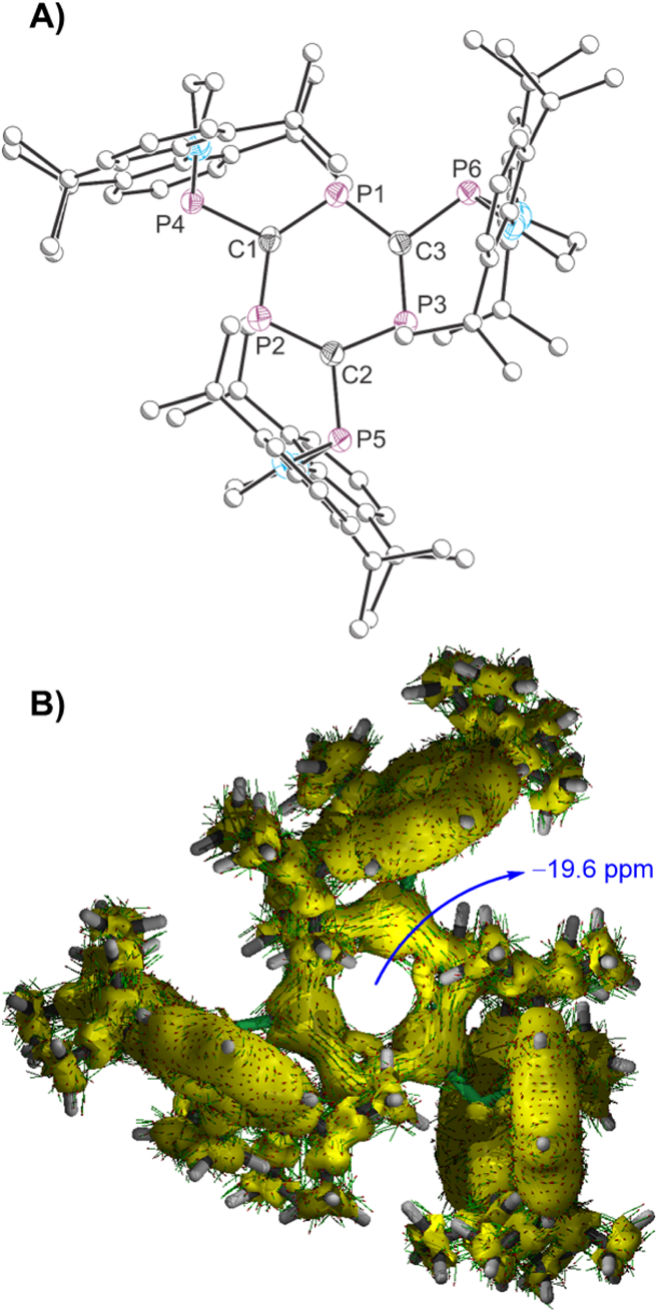
(A) Molecular structure of 1 as determined by SXRD at 173(2) K. Only one of the two crystallographically unique molecules in the asymmetric unit is pictured. Carbon atoms of phosphanyl groups depicted with an arbitrary radius. Thermal ellipsoids pictured at 50% probability level. Hydrogen atoms are removed for clarity. (B) AICD plot and computed NICS(1)_*zz*_ value.

The structure of 1 was determined by single-crystal X-ray diffraction (Fig. 2A).^51*a*–*e*^ The compound features a planar triphosphabenzene core with pendant phosphanyl groups. Due to the large steric profile of these groups, they are oriented in such a manner that the overall molecule has *pseudo-C*_3*h*_ symmetry (broken by the methylene groups of the phosphanyl backbone). Consequently, the phosphorus atom lone pairs of the phosphanyl groups point towards one phosphorus atom of the triphosphabenzene ring and away from another. The core of the molecule is structurally related to other reported triphosphabenzene molecules, with average interring C–P distances of 1.731 Å (*cf.* 1.727 Å for ^*t*^Bu_3_C_3_P_3_).^7^ These values are between those expected for C–P single (1.86 Å) and C=P double bonds (1.69 Å).^23,24^ The interring bond angles alternate between 108.5 and 131.4° for the C–P–C and P–C–P bond angles, respectively (*cf.* 109.3 and 130.7° for ^*t*^Bu_3_C_3_P_3_).^7^ These geometrical features point to a significant electronic-delocalization within the C_3_P_3_ ring. Indeed, the Anisotropy of the Induced Current Density (AICD) method^25,26^ clearly shows the occurrence of a diatropic (*i.e.*, clockwise vectors) ring current delocalized within the C_3_P_3_ ring. As a consequence, the corresponding Nuclear Independent Chemical Shift (NICS) value,^27^ computed at 1 Å above the [3, +1] ring critical point of the electron density,^28^ is highly negative (NICS(1)_*zz*_ = −19.6 ppm), which confirms the aromatic nature of the C_3_P_3_ heterocycle in 1. The NICS(1) value of 1 (−8.4 ppm) compares favourably with reported values for Me_3_C_3_P_3_ and ^*t*^Bu_3_C_3_P_3_.^29,30^

Dissolution of the red crystals of 1 in C_6_D_6_ and acquisition of a ^31^P{^1^H} NMR spectrum revealed a clean spectrum with two doublet resonances integrating in a 1 : 1 ratio at 295.5 and 118.5 ppm. An identical spectrum was obtained when recording the proton-coupled ^31^P NMR spectrum. The chemical shifts of these resonances are comparable to other literature-reported values for 1,3,5-triphosphabenzenes (*cf.* 232.6 ppm for ^*t*^Bu_3_C_3_P_3_)^3^ and compounds bearing the same phosphanyl group (*cf.* 154.0 ppm for [(H_2_C)_2_(NDipp)_2_]PCl).^20^ The observation of a single, remarkably large, spin–spin coupling constant (or *J*-coupling) between ^31^P nuclei (432 Hz) was initially perplexing as, based on the X-ray structure, we would have expected to observe two more complex multiplet resonances with weaker coupling (assuming restricted rotation of the pendant phosphanyl moieties in solution). It is worth highlighting that the magnitude of the observed coupling constant is considerably larger than that observed for related compounds featuring phosphorus-containing aromatic rings with *ortho*-phosphanyl substituents. For instance, Mathey and Le Floch reported an extensive family of 2-phosphanyl-phosphinine derivatives exhibiting ^2^*J*_P–P_ coupling constants in the range of 55.1 to 264.0 Hz.^31,32^ The ^1^H and ^13^C{^1^H} NMR spectra of 1 are consistent with a cyclic structure, and with restricted rotation about the C–P bonds of the phosphanyl substituents (*e.g.* magnetically inequivalent methylene resonances for the phosphanyl ligand backbone and the methine protons of the Dipp isopropyl substituents). Solution-phase atmospheric pressure chemical ionization (AP-CI) mass spectrometry measurements revealed a molecular ion peak at 1357.7602 Da, as expected for the [1 + H]^+^ ion (1357.7603 Da). This suggests that compound 1 remains intact in solution. We thus attribute the large ^31^P–^31^P nuclear spin coupling to a strong “through-space” interaction between the phosphorus atom of the pendant phosphanyl groups and adjacent triphosphabenzene phosphorus nuclei.

“Through-space” (TS) spin–spin coupling (^TS^*J*) differs from “through-bond” coupling (^TB^*J*) as it involves the interaction of nuclear spins through non-bonding electron pairs.^33^ Mallory and co-workers have attributed the magnitude of the TS coupling constant to the effectiveness of the overlap interactions between the lone pair orbitals.^34,35^ The magnitude of the coupling constant observed for 1, *J*(^31^P–^31^P) = 432 Hz, suggests effective overlap of two “lone pair” orbitals both of which have a significant degree of s-orbital character. Such interactions are relatively well established,^36^ and are common in rigid polyphosphines (for example, tetraphosphine ferrocenyl derivatives),^37^ and asymmetric *peri*-substituted bis-(phosphines).^38–40^ However, to the best of our knowledge, the magnitude of the coupling constant observed for 1 is the largest of its kind, and almost double that of other reported interactions. Such interactions have recently attracted considerable attention, and computational efforts to distinguish between “through-space” and “through-bond” interactions have proven to be effective at predicting the magnitude of experimentally determined coupling constants (*vide infra*).^41^

**Scheme 1:**
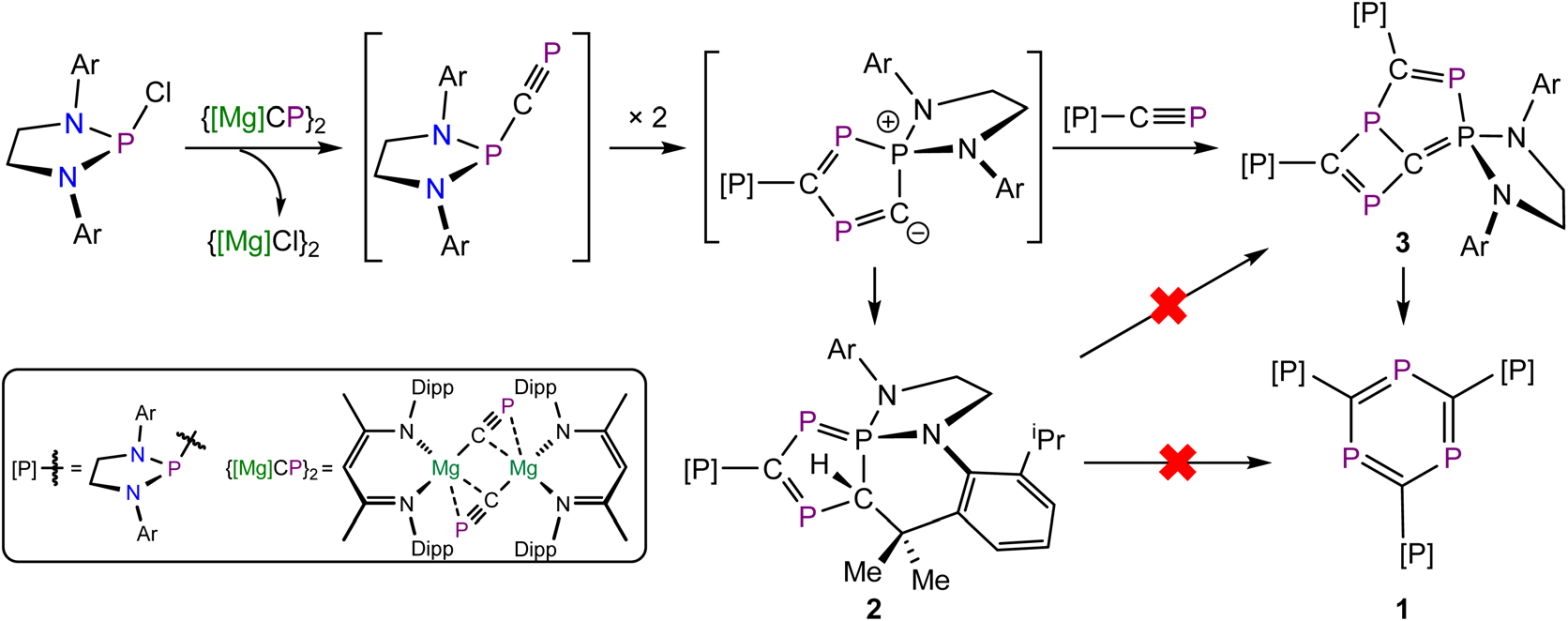
Synthesis of 1–3*via* an intermediate phosphanyl-phosphaalkyne.

**Fig. 3:**
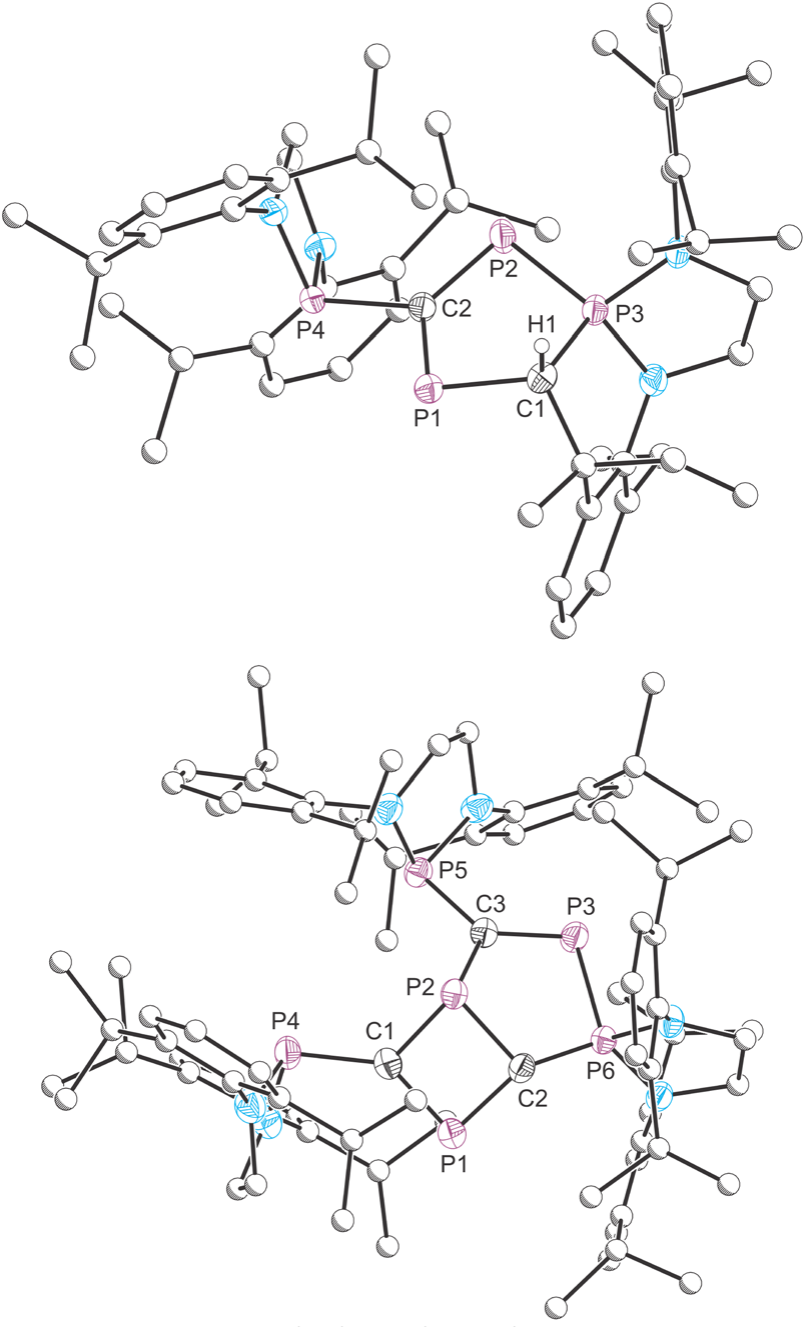
Molecular structures of 2 (top) and 3 (bottom) as determined by SXRD at 173(2) K. Carbon atoms of phosphanyl groups depicted with an arbitrary radius. Thermal ellipsoids pictured at 50% probability level. Hydrogen atoms (except for H1 in 2) are removed for clarity.

To understand the mechanism that afforded 1, we turned our attention to the reaction mixtures from which it was isolated. Monitoring the reaction of [(H_2_C)_2_(NDipp)_2_]PCl and {[Mg]CP}_2_ by ^31^P{^1^H} NMR spectroscopy reveals the formation of 1 over the course of approximately 36 hours (Scheme 1). During the reaction, two additional species were observed in the ^31^P{^1^H} NMR spectra. One of these, compound 2, corresponds to a side-product, and is associated with four multiplet resonances in the ^31^P NMR spectrum at 242.0, 127.3, 107.0 and −40.6 ppm. This species is still present in the final reaction mixture from which 1 was isolated. The other species present in these reaction mixtures, 3, is consumed over time. We attribute these resonances to a long-lived intermediate in the formation of 1. This compound gives rise to six broadened multiplet resonances in the ^31^P{^1^H} NMR spectrum which were observed at 338.6, 303.3, 100.0, 94.1, 83.2 and 55.5 ppm. Compound 2 was isolated by fractional crystallization from the reaction mixtures, while single crystals of intermediate 3 were obtained by storing the reaction mixture at −35 °C (see SI). Samples of both compounds were analyzed by single-crystal X-ray diffraction (Fig. 3).

The molecular structure of 2 (Fig. 3, top) reveals a rather unusual fused tricyclic core (composed of two five-membered rings sharing adjacent edges of a central six-membered heterocycle). On closer inspection, it becomes apparent that this compound is a dimer of the targeted phosphaalkyne [(H_2_C)_2_(NDipp)_2_]P(CP). The five-membered ring composed of atoms P1/C1/P3/P2/C2 can be rationalized as resulting from a [3 + 2] cycloaddition product of two molecules of [(H_2_C)_2_(NDipp)_2_]P(CP). Furthermore, a methine C–H bond of a phosphanyl group has been activated at a carbon atom of the five-membered ring, affording a protonated tertiary carbon center (C1). This five-membered ring has localized P1=C2 (1.701(2) Å) and P2=P3 (2.096(1) Å) double bonds. The P1–C1, P3–C1 and C2–P2 bonds lengths are 1.892(2), 1.824(2), 1.776(2) Å, respectively. The presence of four chemically inequivalent phosphorus atoms is consistent with the observation of four resonances in the crude ^31^P{^1^H} NMR spectrum of the mixture from which this sample was crystallized.

The structure of 3 (Fig. 3, bottom) is an isomer of 1, and thus an alternate trimer of [(H_2_C)_2_(NDipp)_2_]P(CP). The X-ray structure reveals a fused bicyclic core composed of four- and five-membered rings. The four-membered ring is the result of the dimerization of two cyaphide groups and possesses one short (C1–P1: 1.707(3) Å) and three longer C–P bonds (C1–P2: 1.825(3) Å, C2–P2: 1.806(3) Å; C2–P1: 1.780(3) Å). In the adjacent five-membered ring there are also two short C–P bond lengths (C3–P3: 1.698(3) Å, C2–P6: 1.682(3) Å) and two notably longer ones (C2–P2: 1.806(3) Å, C3–P2: 1.803(3) Å). It is worth noting that the transannular P1–P2 bond length is relatively short at 2.566(1) Å, which while longer than the expected length for a P–P single bond (2.22 Å),^23^ is still shorter than the sum of van der Waals radii for two phosphorus atoms (3.72 Å),^42^ and may indicate some degree of bonding interaction.

Based on previous reactivity studies,^15–17^ we propose that the formation of compounds 1–3 proceeds *via* the formation of a phosphanyl-phosphaalkyne, [(H_2_C)_2_(NDipp)_2_]P(CP) (Scheme 1). This species can readily dimerize to afford an unobserved [3 + 2] cycloaddition product, which appears to be a reasonable intermediate to the formation of either 2 or 3 (*vide infra* for a detailed computational investigation). The formation of 2 can be accounted for as the result of an intramolecular C–H activation reaction involving the unobserved dimer and the methine C–H bond of one of the isopropyl groups of the Dipp substituents. Given the irreversibility of this bond activation reaction, the compound cannot be converted to 1 or 3 upon heating. The unobserved dimer can also react with another equivalent of the phosphanyl-phosphaalkyne *via* a [2 + 2] cycloaddition reaction to afford intermediate 3 which we know, based on the *in situ*^31^P NMR studies, ultimately rearranges to afford 1. It should be noted that solutions of 3 ultimately isomerize to 1 over the course of a day at room temperature (Fig. S19).

**Fig. 4:**
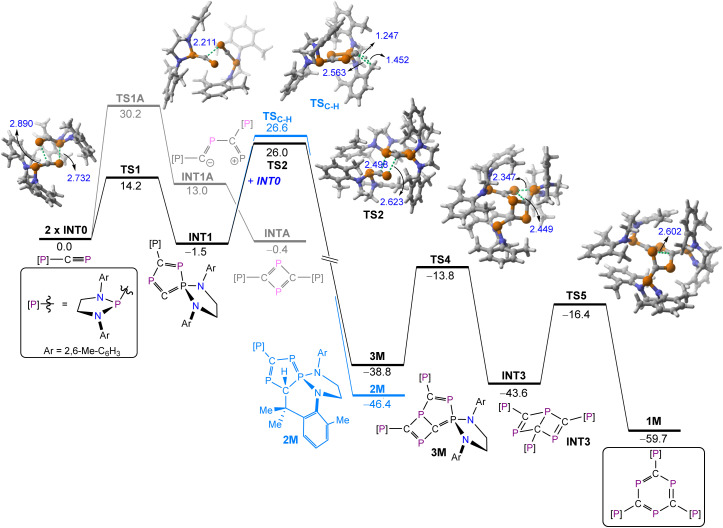
Computed reaction profile for the formation of 1M. Relative free energies (Δ*G*, at 298 K) and bond distances are given in kcal mol^−1^ and angstroms, respectively. All data have been computed at the CPCM(hexane)-M062X/def2-TZVPP//CPCM(hexane)-ωB97x-D3BJ/def2-SVP level.

Density Functional Theory (DFT) calculations at the CPCM(hexane)-M062X/def2-TZVPP//CPCM(hexane)-ωB97x-D3BJ/def2-SVP level were carried out to gain more insight into the mechanism involved in the formation of 1. Fig. 4 shows the computed reaction profile for the formation of the observed triphosphabenzene starting from INT0, a model of the phosphanyl-phosphaalkyne, [(H_2_C)_2_(NDipp)_2_]P(CP), in which the isopropyl groups of the Dipp substituent are replaced by methyl groups. Two different cycloaddition reactions, namely [2 + 2] or [3 + 2], can be envisaged for the initial dimerization of INT0. Although the formation of a [2 + 2] cycloadduct INTA is thermodynamically feasible (Δ*G* = −0.4 kcal mol^−1^), the corresponding transition state associated with a concerted cycloaddition could not be located on the potential energy surface. We thus explored a stepwise reaction and located the corresponding zwitterionic intermediate INT1A, which lies 13.0 kcal mol^−1^ above the separate reactants and is formed *via*TS1A with an activation barrier of 30.2 kcal mol^−1^. Interestingly, we also found a lower lying transition state, TS1 (Δ*G*^≠^ = 14.2 kcal mol^−1^) which directly leads to the formation of the [3 + 2] cycloadduct INT1 in a slightly exergonic reaction (Δ*G* = −1.5 kcal mol^−1^). Therefore, in this [3 + 2] dipolar cycloaddition step one [P]–C≡P moiety acts as a dipole whereas the –C≡P fragment from another molecule of INT0 acts as a dipolarophile. From this intermediate, two alternative processes can be envisaged, namely an intramolecular C–H activation leading to compound 2M or a new cycloaddition reaction with another molecule of INT0 to produce the observed intermediate 3M. Our calculations indicate that the C–H activation reaction occurs through TS_C–H_ with an activation barrier of 28.1 kcal mol^−1^ in a highly exergonic reaction (Δ*G* = −44.9 kcal mol^−1^, from INT1), which confirms the irreversible nature of the transformation. The alternative cycloaddition reaction proceeds with a lower barrier *via*TS2 (Δ*G*^≠^ = 27.5 kcal mol^−1^), also in a highly exergonic reaction (Δ*G* = −37.3 kcal mol^−1^), therefore indicating that this step is kinetically preferred over the alternative C–H activation reaction (despite the entropic penalty associated with the incorporation of a new molecule of INT0), which is consistent with the experimental findings. Intermediate 3M is finally transformed into the final 1,3,5-triphosphabenzene 1M in a stepwise process through the formation of the corresponding Dewar-phosphabenzene intermediate INT3. Thus, intermediate 3M is first transformed into INT3*via*TS4, a saddle point associated with the formation of the new C–P bond with concomitant P–P bond rupture, followed by a new C–P bond breaking *via*TS5 which finally affords the observed triphosphabenzene.^43^ The calculated activation barriers for the final two steps (Δ*G*^≠^ = 25.0 and 27.2 kcal mol^−1^, respectively) along with the exergonic nature of the reactions (Δ*G* = −4.8 and −16.1 kcal mol^−1^, respectively), are consistent not only with a process occurring at room temperature but also with the experimental observation that compound 3 can be readily converted into compound 1 (see above). This is very likely the result of the gain in aromaticity in the process (*i.e.*, reflected in the thermodynamic stability of 1), which constitutes the main driving force of the entire transformation.

**Scheme 2:**
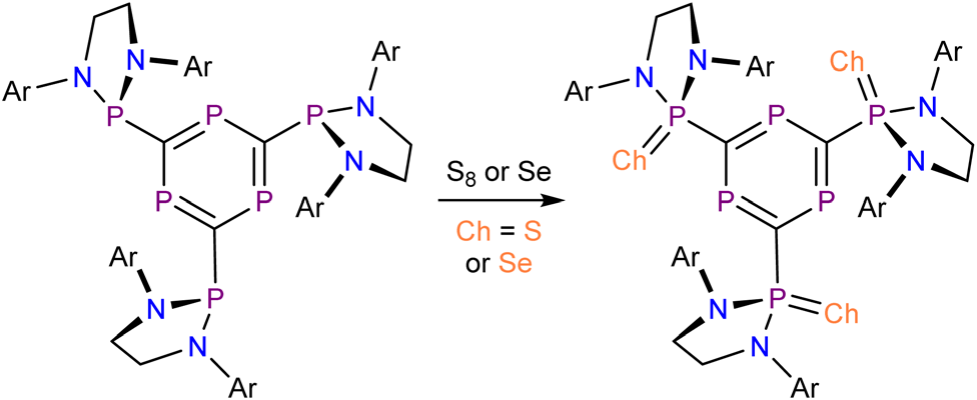
Synthesis of 4 and 5 by oxidation of 1 with either S_8_ or Se.

To further interrogate the “through-space” ^31^P–^31^P nuclear coupling observed for compound 1, we decided to chemically perturb the lone-pair/lone-pair interactions by chemical oxidation of the pendant phosphanyl groups. Reaction of 1 with either elemental sulfur or selenium cleanly affords a new product as evidenced by ^31^P NMR spectroscopy, {[(H_2_C)_2_(NDipp)_2_]PCh}_3_C_3_P_3_ (Ch = S (4), Se (5)). These reactions occur cleanly and quantitatively upon heating the reaction mixtures to 90 °C (Scheme 2).

**Fig. 5:**
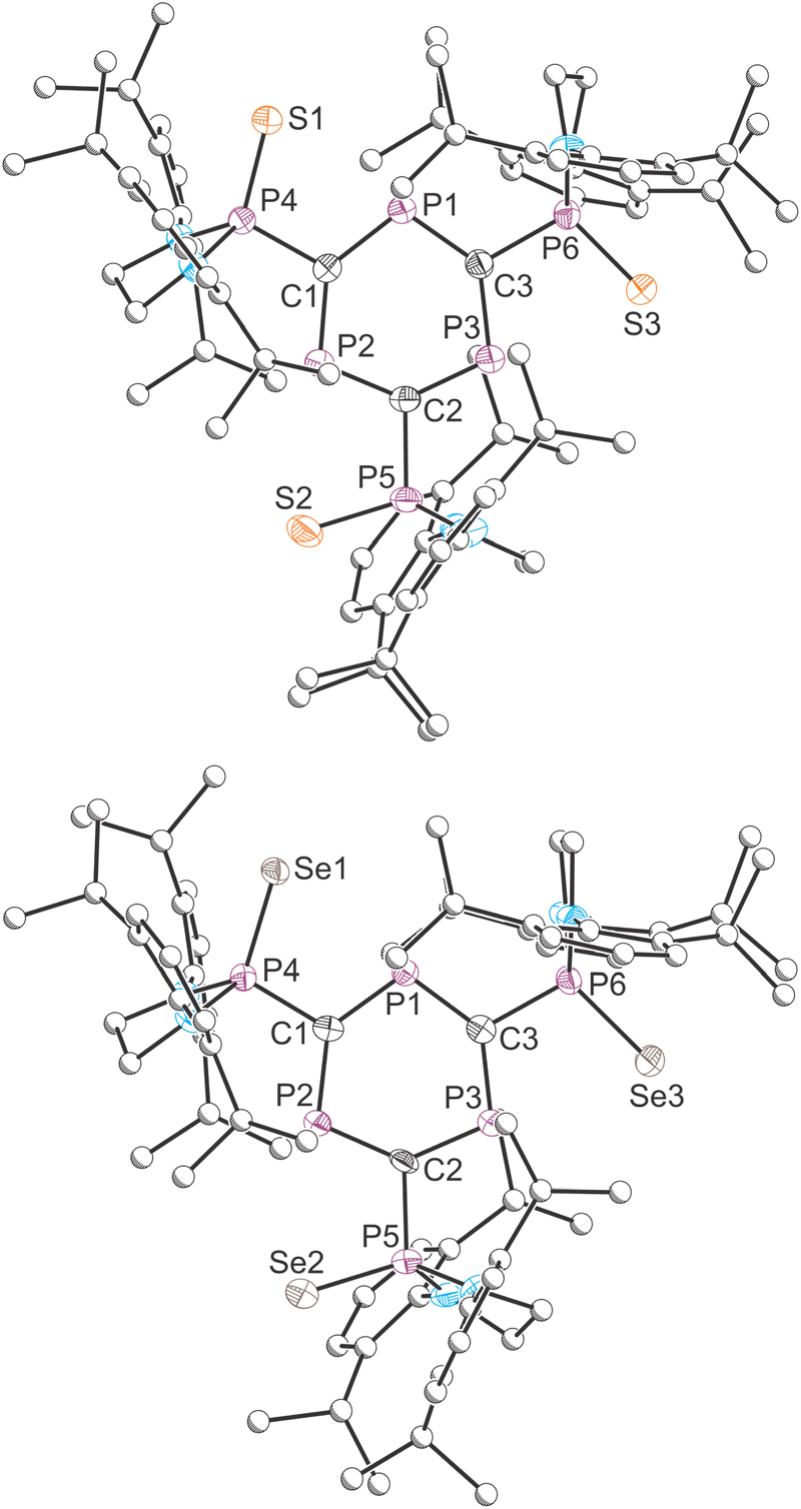
Molecular structures of 4 (top) and 5 (bottom) as determined by SXRD at 173(2) and 143(2) K, respectively. Carbon atoms of phosphanyl groups depicted with an arbitrary radius. Thermal ellipsoids pictured at 50% probability level. Hydrogen atoms are removed for clarity.

The molecular structures of compounds 4 and 5 were determined by X-ray diffraction analysis (Fig. 5). They confirm the oxidation of the pendant phosphanyl groups with *pseudo*-tetrahedral coordination about the *exo*-cyclic phosphorus atoms. Structurally, these compounds closely resemble 1 and other literature reported triphosphabenzenes, with average interring C–P distances of 1.735 and 1.732 Å for 4 and 5, respectively (*cf.* 1.731 Å for 1). The mean C–P bond distances to the pendant groups are also very similar at 1.831 and 1.829 Å for 4 and 5, respectively, as expected for C–P single bonds (1.86 Å).^23^ The P=Ch (Ch = S, Se) bonds exhibit average values of 1.933 Å for 4, and 2.095 Å for 5, which can be accounted for due to the difference in covalent radii between sulfur and selenium (Δ*r*_cov_ = 0.15 Å).^44^

The ^31^P{^1^H} NMR spectrum of 4 reveals two multiplet resonances corresponding to an AA′A′′XX′X′′ type system which were observed at 291.0 and 71.9 ppm. Compound 5 exhibits two similar multiplets at 284.9 and 70.7 ppm, with the added feature that the resonance corresponding to the pendant phosphanyl selenide (at 70.7 ppm) exhibits satellites due to coupling with the ^77^Se nuclei (^1^*J*(^31^P–^77^Se) = 803 Hz). The ^77^Se NMR spectrum of 5 displays a doublet of doublets at 21.9 ppm, arising from ^31^P–^77^Se coupling (^1^*J*(^31^P–^77^Se) = 803 Hz and ^3^*J*(^31^P–^77^Se) = 101 Hz), the observed splitting pattern arises due to the magnetic inequivalence of the phosphorus nuclei of the ring. The large ^1^*J*(^31^P–^77^Se) coupling constant is indicative of significant involvement of the phosphorus s-orbital in the P–Se bond.^45^ The involvement of the phosphanyl lone-pairs in the formation of phosphorus–chalcogen bonds disrupts any possibility of “through-space” coupling and the “through-bond” coupling constants extracted for the observed spectra exhibit magnitudes consistent with two-bond ^2^*J* coupling between phosphorus nuclei (101–168 Hz). The magnitude of these coupling constants is similar to those reported for 2- and 2,6-(phosphanylsulfide)-phosphinines (^2^*J*_P–P_ = 108.1 and 115 Hz, respectively).^46,47^

**Fig. 6:**
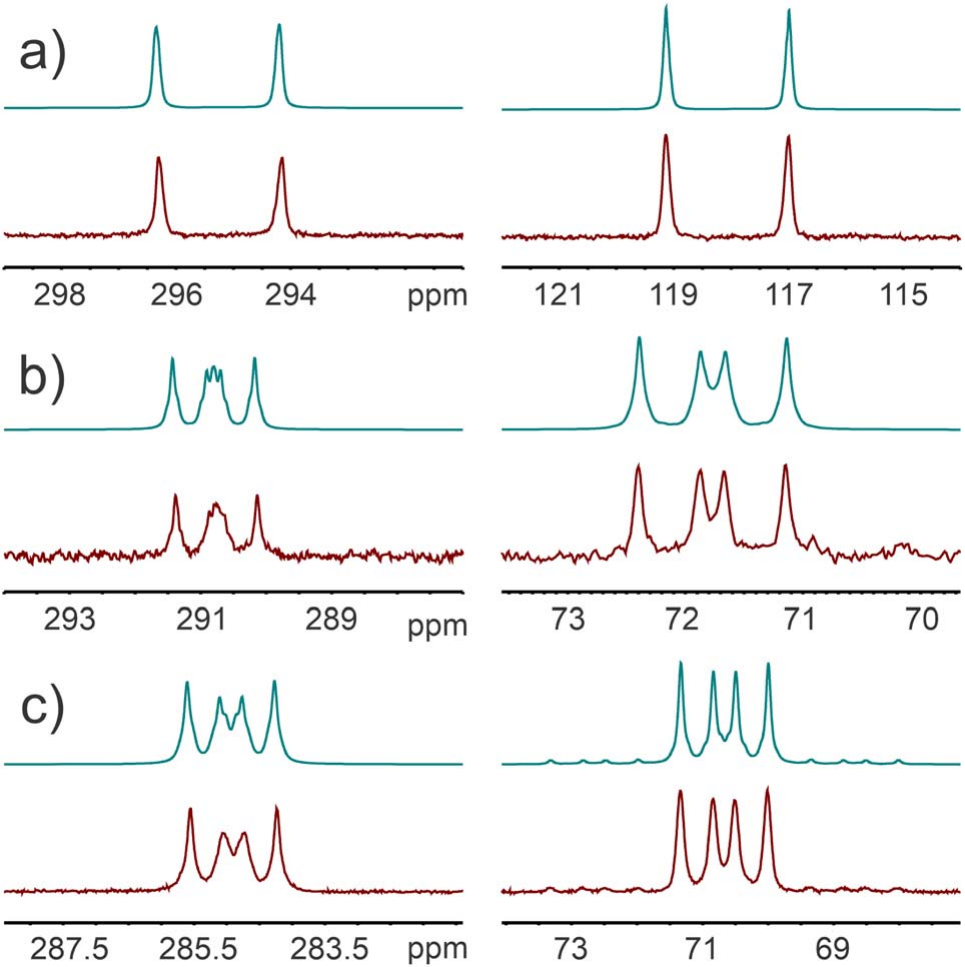
Simulated (top) *vs.* experimental (bottom) ^31^P NMR spectra for: (a) 1; (b) 4; and (c) 5.

To further understand the observed ^31^P NMR spectra of 1, 4 and 5, they were simulated using the gNMR software package (Fig. 6).^48^ From the molecular structures of these compounds, it is apparent that there is restricted rotation of the pendant phosphanyl groups. For any given phosphanyl group, this renders the two nearest phosphorus nuclei of the ring magnetically inequivalent. Thus, we can expect that the ^2^*J*_P–P_ values will be different for these interactions. The other two-bond ^31^P–^31^P coupling interaction that we must consider is between the phosphorus nuclei of the ring. Assuming that any ^4^*J*_P–P_ is ∼0 Hz, and an AA′A′′XX′X′′ spin system we can simulate the observed spectra. In 1, the large s-orbital character of the overlapping lone pair orbitals gives rise to one exceptionally large ^TS^*J* value (432 Hz). There is no observable coupling between the phosphanyl group and the phosphorus atom on its opposite side. The through-bond ^2^*J*_P–P_ coupling of the atoms within the ring was not detected experimentally (but could be approximated as 4 Hz based on the simulation). By contrast, in 4 and 5 (where the lone pair is replaced by sulfur or selenium), we see measurable ^2^*J*_P–P_ coupling between the phosphanyl group and the adjacent phosphorus ring nuclei (4: 147 and 106 Hz; 5: 168 and 101 Hz), and a larger coupling constant between the phosphorus nuclei of the ring (4: 18 Hz; 5: 20 Hz from simulations), which manifests itself as an increase in the linewidth of this resonance. A comparison of these coupling constants and the computed values for 1 (*vide infra*) is provided in the SI (Table S1).

**Fig. 7:**
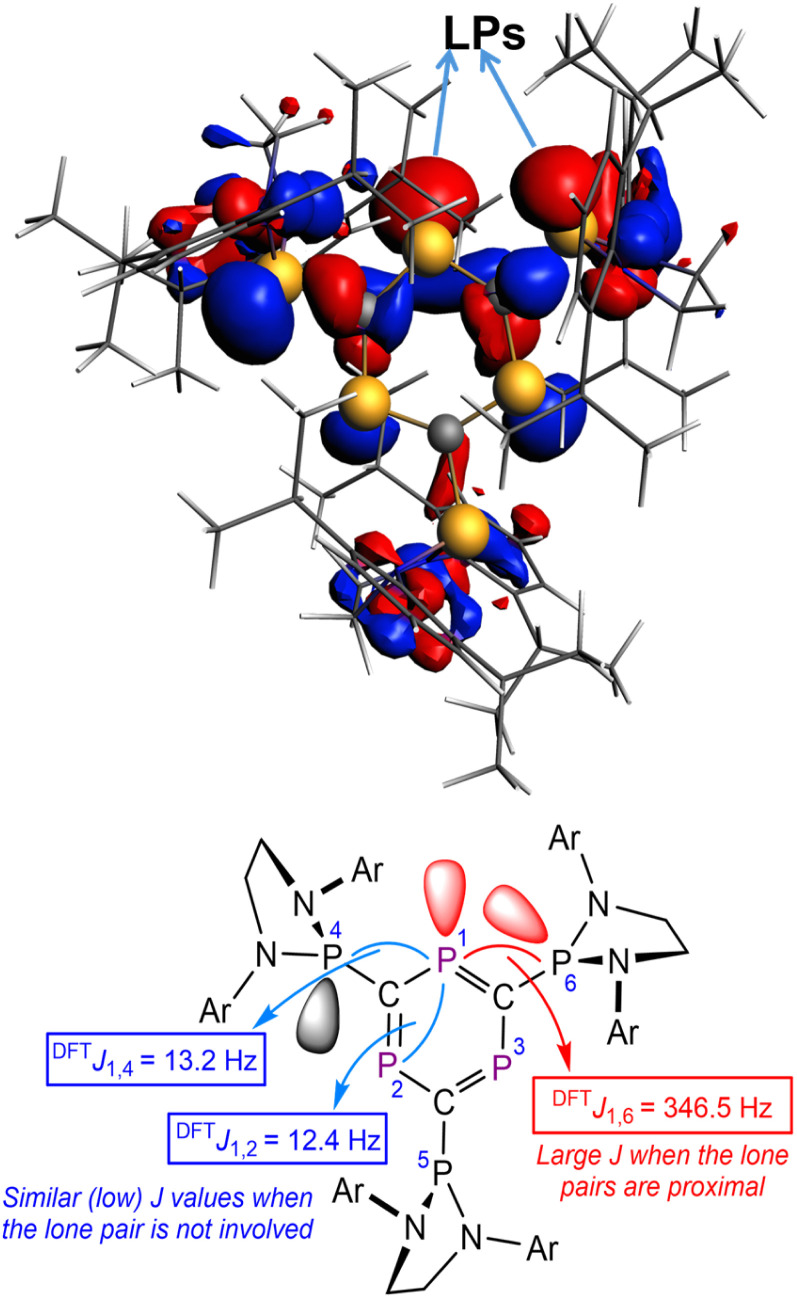
Visualization of the lone-pairs (top, isosurface value of 0.03 au) involved in the through-space coupling in 1, and schematic representation (bottom) of two-bond spin–spin coupling interactions (and their computed coupling constants). All data have been computed at the ωB97x-D3BJ/def2-TZVPP//ωB97x-D3BJ/def2-SVP level.

Additional DFT calculations were carried out to further support the large “through-space” ^31^P–^31^P nuclear coupling observed for compound 1. After an initial benchmark study (see ESI), we found that whereas the BP86-D3BJ functional provides relatively accurate chemical shifts, coupling constants were better reproduced by the ωB97x-D3BJ functional. Our calculations show that when the lone pairs of both the ring and pendant phosphorus atoms are in proximity (P1–P6, see Fig. 7 for atom numbering), a large ^31^P–^31^P coupling is observed (^DFT^*J*_P1–P6_ = 346.5 Hz). In sharp contrast, a much lower value of *ca.* 13 Hz was computed for the coupling between P1–P2 and P1–P4, where the lone pairs of both phosphorus atoms point away from one another, therefore indicating a standard (and weak) “through-bond” coupling. Interestingly, Natural Bond Orbital calculations indicate that, in agreement with our initial hypothesis, these lone pairs feature a significant *s*-character with hybridizations ranging from sp^0.72^ to sp^0.82^ (*i.e.*, 55–58% s-character).

**Fig. 8:**
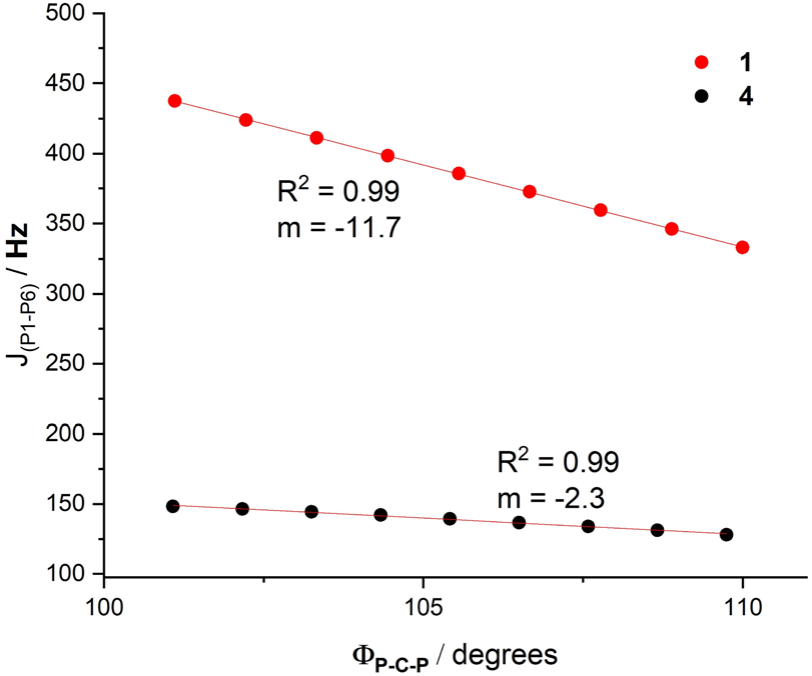
Computed variation of the coupling constants with the P–C–P angle in compounds 1 and 4. All data have been computed at the ωB97x-D3BJ/def2-TZVPP//ωB97x-D3BJ/def2-SVP level.

To further investigate the nature of the coupling, and following the previous work by Kaupp, Malkina and co-workers,^49,50^ we computed the variation of the ^31^P–^31^P coupling in species 1 and 4 at different *Φ*_P–C–P_ angles. As shown in Fig. 8, whereas the initially high coupling constant significantly decreases in 1 as the angle becomes larger (slope of −11.7 Hz per degree), it remains much lower and practically unaltered in species 4 (slope of −2.3 Hz per degree). This finding not only confirms that the “through-space” coupling is markedly inhibited in species 4 but also that it is strongly dependent on the overlap between the phosphorus lone-pairs in 1, which decreases as the P1–C–P6 torsion angle increases. This explains why compounds with comparable substitution patterns to 1, such as the phosphanyl-phosphinines reported by Mathey and Le Floch, exhibit significantly smaller coupling constants, as they are free to rotate the lone pairs away from one another. By contrast, for 1, the steric demands of the phosphanyl substituents prevent their free rotation and enforce the orbital overlap that gives rise to the unique through-space nuclear spin interaction observed.

## Conclusions

We have shown that cyaphide group transfer to a bis(aryl-amino)chlorophosphine gives rise to a transient phosphanyl-phosphaalkyne, which cyclotrimerizes over time to afford a 2,4,6-tri(phosphanyl)-1,3,5-triphospha-benzene. Due to the significant steric bulk of the pendant phosphanyl groups, the molecule is locked into a *pseudo-C*_3*h*_ geometry which forces the lone pairs of the phosphanyl groups and the triphosphabenzene phosphorus atoms into close proximity. This close contact is manifested in a remarkably large “through-space” spin–spin coupling between the phosphorus nuclei. The remarkably large value of this coupling constant, ^TS^*J* = 432 Hz, suggests both effective orbital overlap between the lone-pairs, and a significant degree of s-orbital participation in each of the molecular orbitals involved in this interaction. This hypothesis was probed through computational studies using density functional theory. The “through-space” interaction can be disrupted by oxidation of the pendant phosphanyl groups using either elemental sulfur or selenium. In the case of these compounds, engaging the phosphanyl lone-pair in a bond gives rise to the “through-bond” coupling interactions that would be expected for such systems.

## Experimental section

### General synthetic methods

All reactions and product manipulations were carried out using standard Schlenk-line techniques under an inert atmosphere of argon, or in a dinitrogen filled glovebox (MBraun UNIlab glovebox maintained at <0.1 ppm H_2_O and <0.1 ppm O_2_). [(H_2_C)_2_(NDipp)_2_]PCl (Dipp = 2,6-diisopropylphenyl)^20^ and [Mg(^Dipp^NacNac)(CP)]_2_ ({[Mg]CP}_2_)^21^ were synthesized according to previously reported synthetic procedures. Toluene (Fisher Chemical, HPLC grade), hexane (Fisher Chemical, HPLC grade) and pentane (Fisher Chemical, HPLC grade) were purified using a Pure Process Technology (PPT) solvent purification system (SPS). C_6_D_6_ (Aldrich, 99.5%) was distilled over sodium/benzophenone. All dry solvents were stored under argon in gas-tight ampoules over activated 3 Å molecular sieves.

### Analytical techniques

NMR spectra were acquired on a Bruker 500 MHz Avance Neo, a Varian 400 MHz Inova NMR spectrometer, or a Varian 600 MHz Inova NMR spectrometer. Chemical shifts (*δ*) are reported in parts per million (ppm). ^1^H and ^13^C NMR spectra are referenced to TMS using the most downfield protio-solvent resonance (^1^H NMR C_6_D_6_: *δ* = 7.16 ppm, ^13^C NMR C_6_D_6_: *δ* = 128.06 ppm). High-resolution mass spectra were recorded on a Thermo Q-Exactive Plus (APCI-Orbitrap, positive ion mode) instrument at the Mass Spectrometry Facility of the Department of Chemistry of Indiana University. IR spectra were acquired on a Thermo Scientific Nicolet Summit FTIR spectrometer with a diamond ATR stage. A solution/suspension of the samples in pentane was drop-casted onto the ATR crystal and dried by evaporation inside of a glovebox under a dinitrogen atmosphere prior to the collection of spectra. X-ray diffraction data were collected with a Bruker D8 Venture diffractometer equipped with a PhotonII detector and IμS sources, using either Mo K_α_ or Cu K_α_ irradiation and various experiment temperatures, collection strategies, and exposure times. Details are summarized in the CIF and below.

### Synthesis of {[(H_2_C)_2_(NDipp)_2_]P}_3_C_3_P_3_ (1)

A mixture of [(H_2_C)_2_(NDipp)_2_]PCl (98.8 mg, 0.22 mmol) and a small excess of [Mg(^Dipp^NacNac)(CP)]_2_ (118.8 mg, 0.12 mmol), were suspended in hexane (5 ml) at −78 °C. The mixture was stirred at −78 °C for 2 h and then allowed to reach room temperature. The resulting red suspension was vigorously stirred for 2 days before it was filtered. The red filtrate was concentrated by slow evaporation at room temperature to an approx. volume of 1 ml, affording crystals of 1. Yield: 25.9 mg (0.09 mmol, 42%). HRMS (*m*/*z*): [M + H]^+^ calc. for C_81_H_115_N_6_P_6_, 1357.76034; found 1357.7602. ^1^H NMR (500 MHz, C_6_D_6_, 298.K): *δ* (ppm) 7.18 (dd, ^3^*J*_H–H_ = 7.7 Hz, ^4^*J*_H–H_ = 1.7 Hz, 6H; *meta*-Dipp, partially overlapped with residual solvent resonance), 7.12 (t, ^3^*J*_H–H_ = 7.7 Hz, 6H; *para*-Dipp), 6.83 (dd, ^3^*J*_H–H_ = 7.7, ^4^*J*_H–H_ = 1.7 Hz, 6H; *meta*-Dipp), 4.13–4.01 (m, 12H; NC*H*_2_ and C*H*(CH_3_)_2_), 3.55 (sept, ^3^*J*_H–H_ = 6.8 Hz, 6H; C*H*(CH_3_)_2_), 3.31–3.22 (m, 6H; NC*H*_2_), 1.67 (d, ^3^*J*_H–H_ = 6.8 Hz, 18H; CH(C*H*_3_)_2_), 1.34 (d, ^3^*J*_H–H_ = 6.8 Hz, 18H; CH(C*H*_3_)_2_), 1.10 (d, ^3^*J*_H–H_ = 6.8 Hz, 18H; CH(C*H*_3_)_2_), −0.28 (d, ^3^*J*_H–H_ = 6.8 Hz, 18H; CH(C*H*_3_)_2_). ^13^C{^1^H} NMR (126 MHz, C_6_D_6_, 298 K): *δ* (ppm) 149.40 (d, *J*_C–P_ = 2.2 Hz; *ortho*-Dipp), 148.04 (s; *ortho*-Dipp), 139.11 (d, *J*_C–P_ = 14.7 Hz; *ipso*-Dipp), 127.01 (s; *para*-Dipp), 124.80 (s; *meta*-Dipp), 124.39 (s; *meta*-Dipp), 56.94 (d, *J*_C–P_ = 5.9 Hz; N*C*H_2_), 29.35 (d, *J*_C–P_ = 14.2 Hz; *C*H(CH_3_)_2_), 28.39 (br; *C*H(CH_3_)_2_), 25.71 (s; CH(*C*H_3_)_2_), 25.41 (s; CH(*C*H_3_)_2_), 25.02 (d, *J*_C–P_ = 4.6 Hz; CH(*C*H_3_)_2_), 24.83 (s; CH(*C*H_3_)_2_). ^31^P NMR (202 MHz, C_6_D_6_, 298 K): *δ*(ppm) 295.5 (d, ^TS^*J*_P–P_ = 432 Hz; C_3_*P*_3_), 118.5 (d, ^TS^*J*_P–P_ = 432 Hz; N_2_*P*C).

### Synthesis of 2

[Mg(^Dipp^NacNac)(CP)]_2_ (118.8 mg, 0.12 mmol) was suspended in toluene (5 ml) and cooled to −78 °C. To this suspension, a solution of [(H_2_C)_2_(NDipp)_2_]PCl (98.5 mg, 0.22 mmol) in toluene (15 ml) was added. The mixture was stirred at −78 °C for 2 h and then allowed to reach room temperature. The resulting red suspension was vigorously stirred for 2 days at room temperature. All volatiles were removed under vacuum to obtain a red solid that was washed with pentane (5 × 1.5 ml). The red solid was extracted with toluene (1 ml) and all the volatiles were removed *in vacuo*. Again, the resulting solid was washed with pentane (1 ml) to yield product 2 as a red solid. Yield: 9.8 mg (0.011 mmol, 10%). HRMS (*m*/*z*): [M + H]^+^ calc. for C_54_H_77_N_4_P_4_, 905.50932; found 905.5087. ^1^H NMR (500 MHz, C_6_D_6_, 298 K): *δ* (ppm) 7.26 (t, ^3^*J*_H–H_ = 7.7 Hz, 1H; *para*-Dipp), 7.23–7.05 (m, 8H; aromatic-Dipp), 6.82 (dd, ^3^*J*_H–H_ = 7.7 Hz, ^4^*J*_H–H_ = 1.7 Hz, 1H; *meta*-Dipp), 6.80 (dd, ^3^*J*_H–H_ = 7.7 Hz, ^4^*J*_H–H_ = 1.7 Hz, 1H; *meta*-Dipp), 6.73 (t, ^3^*J*_H–H_ = 7.7 Hz, 1H; *para*-Dipp) 4.70 (br, 1H; C*H*(CH_3_)_2_), 4.12–4.02 (m, 2H; NC*H*_2_), 4.02–3.94 (m, 1H; NC*H*_2_), 3.89 (s, br, 1H; C*H*(CH_3_)_2_), 3.84 (s, br, 1H; C*H*(CH_3_)_2_), 3.77 (sept, ^3^*J*_H–H_ = 6.7 Hz, 1H; C*H*(CH_3_)_2_), 3.63–3.50 (m, 3H; P_2_C*H*C, NC*H*_2_ and C*H*(CH_3_)_2_), 3.36 (sept, ^3^*J*_H–H_ = 6.6 Hz, 1H; C*H*(CH_3_)_2_), 3.32–3.25 (m, 1H; NC*H*_2_), 3.22–3.12 (m, 2H; C*H*(CH_3_)_2_ and NC*H*_2_), 3.00–2.91 (m, 1H; NC*H*_2_), 2.72–2.63 (m, 1H; NC*H*_2_), 1.75–1.69 (m, 6H; C(C*H*_3_)_2_ and CH(C*H*_3_)_2_), 1.55 (s, 3H; C(C*H*_3_)_2_), 1.49 (br, 6H; CH(C*H*_3_)_2_), 1.39 (br, 3H; CH(C*H*_3_)_2_), 1.32 (d, ^3^*J*_H–H_ = 6.7 Hz, 3H; CH(C*H*_3_)_2_), 1.25 (br, 9H; CH(C*H*_3_)_2_), 1.16–1.01 (m, 15H; CH(C*H*_3_)_2_), 0.67 (br, 3H; CH(C*H*_3_)_2_). ^13^C{^1^H} NMR (126 MHz, C_6_D_6_, 298 K): *δ* (ppm) 150.49 (m; aromatic-Dipp), 149.73 (m; aromatic-Dipp), 149.37 (d, *J*_C–P_ = 1.3 Hz; *ortho*-Dipp), 148.94 (d, *J*_C–P_ = 1.8 Hz; *ortho*-Dipp), 148.39 (m; aromatic-Dipp), 147.97 (m; aromatic-Dipp), 144.79 (d, *J*_C–P_ = 5.1 Hz; *ortho*-Dipp), 142.87 (d, *J*_C–P_ = 3.8 Hz; *ipso*-Dipp), 140.83 (s; aromatic-Dipp), 140.71 (s; aromatic-Dipp), 140.55 (d, *J*_*C*–P_ = 3.9 Hz; *ortho*-Dipp), 140.07 (s; aromatic-Dipp), 139.96 (s; aromatic-Dipp), 136.58 (d, *J*_C–P_ = 5.8 Hz; *ipso*-Dipp), 126.67 (s; aromatic-Dipp), 126.46 (s; aromatic-Dipp), 126.06 (s; *meta*-Dipp), 125.66 (s; *ortho*-Dipp), 125.19 (s; *meta*-Dipp), 124.74 (s; *para*-Dipp), (s; *meta*-Dipp), 123.13 (s; *meta*-Dipp), 65.95 (ddd, *J* = 62.5, 31.0, 14.9 Hz, P_2_*C*HC), 56.44 (d, *J*_C–P_ = 5.0 Hz; N*C*H_2_), 55.72 (d, *J*_C–P_ = 7.1 Hz; N*C*H_2_), 51.26 (d, *J*_C–P_ = 6.5 Hz; N*C*H_2_), 50.92 (d, *J*_C–P_ = 4.2 Hz; N*C*H_2_), 38.63 (d, *J*_C–P_ = 4.4 Hz; (CHP_2_)*C*(CH_3_)_2_), 33.94 (dd, *J*_*C*–P_ = 20.2, 6.9 Hz; (CHP_2_)C(*C*H_3_)_2_), 32.92 ((CHP_2_)C(*C*H_3_)_2_), 29.14 (s; *C*H(CH_3_)_2_), 29.04 (s; *C*H(CH_3_)_2_), 28.80 (s; *C*H(CH_3_)_2_), 28.56 (s; *C*H(CH_3_)_2_), 28.37 (s; CH(*C*H_3_)_2_), 26.86 (s; CH(*C*H_3_)_2_), 26.70 (s; CH(*C*H_3_)_2_), 26.57 (s; CH(*C*H_3_)_2_), 26.21(s; CH(*C*H_3_)_2_), 25.97 (s; CH(*C*H_3_)_2_), 25.75 (s; CH(*C*H_3_)_2_), 25.49 (s; CH(*C*H_3_)_2_), 25.32 (s; CH(*C*H_3_)_2_), 24.48 (s; CH(*C*H_3_)_2_), 24.12 (dd, *J*_C–P_ = 16.3, 3.0 Hz; CH(*C*H_3_)_2_), 22.89 (s; CH(*C*H_3_)_2_). ^31^P NMR (202 MHz, C_6_D_6_, 298 K): *δ* (ppm) 242.0 (d, *J*_P–P_ = 416 Hz; (CH)*P*(CP_2_)), 127.3 (dt, *J*_P–P_ = 560, 19 Hz; (R_2_N)_2_*P*=P(CH)), 107.0 (dd, *J*_P–P_ = 416, 19 Hz; (R_2_N)_2_*P*(CP_2_)), −40.6 (d, *J*_P–P_ = 560 Hz, P=*P*(CP_2_)).

### Synthesis of intermediate 3

In a glovebox, [(H_2_C)_2_(NDipp)_2_]PCl (43 mg; 0.10 mmol) was dissolved in hexane (2 ml) and added to solid [Mg(^Dipp^NacNac)(CP)]_2_ (50 mg; 0.05 mmol) at −35 °C. The suspension was stored at −35 °C. After one week the suspension was filtered to yield a red solution. Storage of this solution at −35 °C for two weeks resulted in the formation of a few dark red crystals of 3 suitable for single-crystal X-ray diffraction. Crystals of 3 were dissolved in C_6_D_6_ at room temperature and monitored by ^31^P NMR spectroscopy until complete conversion to 1 was observed (≈24 h). HRMS (*m*/*z*): [M + H]^+^ calc. for C_81_H_115_N_6_P_6_, 1357.76034; found 1357.7593. ^31^P NMR (202 MHz, C_6_D_6_, 298 K): *δ* (ppm) 338.6 (m), 303.3 (dt, *J*_P–P_ = 347, 41 Hz), 100.0 (m), 94.17 (m), 83.2 (dd, *J*_P–P_ = 138, 31 Hz), 55.5 (dd, *J*_P–P_ = 347, 56 Hz). All resonances in the spectrum showed multiple smaller couplings which could not be resolved. Resonances labelled as multiplets are broad and no coupling data could be resolved.

### Synthesis of {[(H_2_C)_2_(NDipp)_2_]P(S)}_3_C_3_P_3_ (4)

To a solution of 1 (5.8 mg; 4.27 μmol) in toluene (0.5 ml) in an NMR tube equipped with an air-tight J. Young valve, an excess of elemental sulfur (3 mg, 94 μmol) was added. The reaction mixture was heated to 90 °C for 36 h and monitored by ^31^P NMR spectroscopy. On completion of the reaction, the solvent was removed *in vacuo* and the remaining solid was washed with pentane (2 × 0.2 ml). Crystals of 4 suitable for X-ray diffraction were obtained from a concentrated hexane solution (0.2 ml) at −35 °C after two weeks. Yield: 4.0 mg (2.75 μmol, 64%). HRMS (*m*/*z*): [M + H]^+^ calc. for C_81_H_115_N_6_P_6_^32^S_3_, 1453.67656; found 1453.6779. ^1^H NMR (500 MHz, C_6_D_6_, 298 K): *δ* (ppm) 7.23 (dd, ^3^*J*_H–H_ = 7.7 Hz, ^4^*J*_H–H_ = 1.7 Hz, 6H; *meta*-Dipp), 7.17 (t, ^3^*J*_H–H_ = 7.7 Hz, 6H; *para*-Dipp), 6.91 (dd, ^3^*J*_H–H_ = 7.7 Hz, ^4^*J*_H–H_ = 1.7 Hz, 6H; *meta*-Dipp), 4.43 (sept, ^3^*J*_H–H_ = 6.7 Hz, 6H; C*H*(CH_3_)_2_), 4.04–3.92 (m, 6H; NC*H*_2_), 3.57–3.45 (m, 6H; NC*H*_2_), 3.43–3.30 (m, 6H; CH(C*H*_3_)_2_), 1.71 (d, ^3^*J*_H–H_ = 6.7 Hz, 18H; CH(C*H*_3_)_2_), 1.19 (d, ^3^*J*_H–H_ = 6.7 Hz, 18H; CH(C*H*_3_)_2_), 1.01 (d, ^3^*J*_H–H_ = 6.7 Hz, 18H; CH(C*H*_3_)_2_), 0.22 (d, ^3^*J*_H–H_ = 6.7 Hz, 18H; CH(C*H*_3_)_2_). ^13^C{^1^H} NMR (126 MHz, C_6_D_6_, 298 K): *δ* (ppm) 150.47 (s; *ortho*-Dipp), 149.69 (d, *J*_C–P_ = 2.7 Hz; *ortho*-Dipp), 135.90 (d, *J*_C–P_ = 4.6 Hz; *ipso*-Dipp), 128.4–127.1 (*para*-Dipp overlapped with residual solvent resonance), 124.60 (s; *meta*-Dipp), 124.35 (s; *meta*-Dipp), 53.51 (d, *J*_C–P_ = 8.3 Hz; N*C*H_2_), 29.76 (s; *C*H(CH_3_)_2_), 29.16 (m; *C*H(CH_3_)_2_), 26.79 (s; CH(*C*H_3_)_2_), 26.23 (s; CH(*C*H_3_)_2_), 23.95 (s; CH(*C*H_3_)_2_), 23.76 (s; CH(*C*H_3_)_2_). ^31^P NMR (202 MHz, C_6_D_6_, 298 K): *δ* (ppm) 291.0 (dd, ^2^*J*_P–P_ = 147; 106 Hz; C_3_*P*_3_), 71.9 (dd, ^2^*J*_P–P_ = 147, 106 Hz; *P*=S).

### Synthesis of {[(H_2_C)_2_(NDipp)_2_]P(Se)}_3_C_3_P_3_ (5)

To a solution of 1 (5.7 mg; 4.20 μmol) in toluene (0.5 ml) in an NMR tube equipped with an air-tight J. Young valve, an excess of grey selenium (5 mg, 64 μmol) was added. The reaction was heated to 90 °C for 1 week and monitored by ^31^P NMR spectroscopy. On completion of the reaction, the mixture was reduced to dryness under a dynamic vacuum, and the product was extracted with hexane (1 ml). The resulting solution was stored at −35 °C affording dark brown crystals after 1 month. Yield: 2.4 mg (1.5 μmol, 36% yield). HRMS(*m*/*z*): [M + H]^+^ calc. for C_81_H_115_N_6_P_6_^78^Se^80^Se_2_, 1595.51070; found 1595.5158. ^1^H NMR (500 MHz, C_6_D_6_, 298 K): *δ* (ppm) 7.23 (dd, ^3^*J*_H–H_ = 7.6 Hz, ^4^*J*_H–H_ = 1.8 Hz, 6H; *meta*-Dipp), 7.19 (t, ^3^*J*_H–H_ = 7.6 Hz, 6H; *para*-Dipp), 6.96 (dd, ^3^*J*_H–H_ = 7.6 Hz, ^4^*J*_H–H_ = 1.8 Hz, 6H; *meta*-Dipp), 4.35 (sept, ^3^*J*_H–H_ = 6.8 Hz, 6H; C*H*(CH_3_)_2_), 4.08–3.93 (m, 6H; NC*H*_2_), 3.55–3.42 (m, 6H; NC*H*_2_), 3.37–3.20 (m, 6H; CH(C*H*_3_)_2_), 1.71 (d, ^3^*J*_H–H_ = 6.8 Hz, 18H; CH(C*H*_3_)_2_), 1.18 (d, ^3^*J*_H–H_ = 6.8 Hz, 18H; CH(C*H*_3_)_2_), 0.99 (d, ^*3*^*J*_*H*–H_ = 6.8 Hz, 18H; CH(C*H*_3_)_2_), 0.37 (d, ^3^*J*_H–H_ = 6.8 Hz, 18H; CH(C*H*_3_)_2_). ^13^C{^1^H} NMR (126 MHz, C_6_D_6_, 298 K): *δ* (ppm) 187.19 (dd, *J*_C–P_ = 167.5, 95.1 Hz; (P*C*P)_3_), 150.72 (s; *ortho*-Dipp), 150.08 (d, *J*_C–P_ = 3.0 Hz; *ortho*-Dipp), 136.59 (d, *J*_C–P_ = 5.0 Hz; *ipso*-Dipp), 128.4–127.1 (*para*-Dipp overlapped with residual solvent resonance), 125.06 (s; *meta*-Dipp), 124.77 (s; *meta*-Dipp), 54.53 (d, *J*_C–P_ = 8.2 Hz; N*C*H_2_), 30.22 (s; *C*H(CH_3_)_2_), 29.63 (d, *J*_C–P_ = 8.9 Hz; *C*H(CH_3_)_2_), 27.23 (s; CH(*C*H_3_)_2_), 26.71 (s; CH(*C*H_3_)_2_), 24.40 (s; CH(*C*H_3_)_2_), 24.22 (s; CH(*C*H_3_)_2_). ^31^P NMR (202 MHz, C_6_D_6_, 298 K): *δ* (ppm) 284.9 (dd, ^2^*J*_P–P_ = 168, 101 Hz; C_3_*P*_3_), 70.7 (dd, ^2^*J*_P–P_ = 168, 101 Hz, satellites ^1^*J*_P–Se_ = 803 Hz; *P*=Se). ^77^Se NMR (95 MHz, C_6_D_6_, 298 K): *δ* 21.89 (dd, *J*_P–Se_ = 803, 103 Hz).

## Author contributions

Conceptualization: D. C. M., A. G.-R. and J. M. G.; experimental work: D. C. M. and A. G.-R.; X-ray crystallography: A. G.-R., A. L. and J. M. G.; computational modelling: D. G.-P. and I. F.; NMR simulations: N. H. R.; writing – original draft: D. C. M. and J. M. G.; writing & editing: all authors; supervision: J. M. G.; funding acquisition: J. M. G.

## Conflicts of interest

There are no conflicts to declare.

## Supplementary Material

SC-017-D5SC07729J-s001

SC-017-D5SC07729J-s002

## Data Availability

CCDC 2477493 (for 1·2hex), 2477494 (for 2), 2477495 (for 3·hex), 2477496 (for 4), and 2477497 (for 5·3hex) contain the supplementary crystallographic data for this paper.^51*a*–*e*^ The data supporting this article have been included as part of the supplementary information (SI). Supplementary information: experimental details, spectra, computational information, and crystallographic data. See DOI: https://doi.org/10.1039/d5sc07729j.

## References

[cit1] Barron A. R., Cowley A. H. (1987). Angew Chem. Int. Ed. Engl..

[cit2] Milczarek R., Rüsseler W., Binger P., Jonas K., Angermund K., Krüger C., Regitz M. (1987). Angew Chem. Int. Ed. Engl..

[cit3] Binger P., Leininger S., Stannek J., Gabor B., Mynott R., Bruckmann J., Kruger C., Leininger S. (1995). Angew. Chem., Int. Ed..

[cit4] Tabellion F., Nachbauer A., Leininger S., Peters C., Preuss F., Regitz M. (1998). Angew. Chem., Int. Ed..

[cit5] Tabellion F., Peters C., Fischbeck U., Regitz M., Preuss F. (2000). Chem. Eur. J..

[cit6] Peters C., Tabellion F., Nachbauer A., Fischbeck U., Preuss F., Regitz M. (2001). Z. Naturforsch. B.

[cit7] Gleiter R., Lange H., Binger P., Stannek J., Kruger C., Bruckmann J., Zenneck U., Kummer S. (1998). Eur. J. Inorg. Chem..

[cit8] Ionkin A. S., Marshall W. J., Fish B. M., Schiffhauer M. F., Davidson F., McEwen C. N. (2009). Organometallics.

[cit9] Arnold P. L., Cloke F. G. N., Hitchcock P. B., Nixon J. F. (1996). J. Am. Chem. Soc..

[cit10] Falconer R. L., Russell C. A. (2015). Coord. Chem. Rev..

[cit11] Longobardi L. E., Russell C. A., Green M., Townsend N. S., Wang K., Holmes A. J., Duckett S. B., McGrady J. E., Stephan D. W. (2014). J. Am. Chem. Soc..

[cit12] Goicoechea J. M., Grützmacher H. (2018). Angew. Chem., Int. Ed..

[cit13] Suter R., Mei Y., Baker M., Benkő Z., Li Z., Grützmacher H. (2017). Angew. Chem., Int. Ed..

[cit14] Abels A. S., Eiler F., Le Corre G., Jurt P., Wörle M., Verel R., Benkő Z., Grützmacher H. (2023). Dalton Trans..

[cit15] Görlich T., Coburger P., Yang E. S., Goicoechea J. M., Grützmacher H., Müller C. (2023). Angew. Chem., Int. Ed..

[cit16] Wilson D. W. N., Urwin S. J., Yang E. S., Goicoechea J. M. (2021). J. Am. Chem. Soc..

[cit17] Yang E. S., Wilson D. W. N., Goicoechea J. M. (2023). Angew. Chem., Int. Ed..

[cit18] Zhang Y., Tham F. S., Nixon J. F., Taylor C., Green J. C., Reed C. A. (2008). Angew. Chem., Int. Ed..

[cit19] Frison G., Sevin A., Avarvari N., Mathey F., Le Floch P. (1999). J. Org. Chem..

[cit20] Abrams M. B., Scott B. L., Baker R. T. (2000). Organometallics.

[cit21] Wannipurage D. C., Yang E. S., Chivington A. D., Fletcher J., Ray D., Yamamoto N., Pink M., Goicoechea J. M., Smith J. M. (2024). J. Am. Chem. Soc..

[cit22] Liu L., Ruiz D. A., Munz D., Bertrand G. (2016). Chem.

[cit23] Pyykkö P., Atsumi M. (2009). Chem. Eur. J..

[cit24] Pyykkö P., Atsumi M. (2009). Chem. Eur. J..

[cit25] Herges R., Geuenich D. (2001). J. Phys. Chem. A.

[cit26] Geuenich D., Hess K., Köhler F., Herges R. (2005). Chem. Rev..

[cit27] Chen Z., Wannere C. S., Corminboeuf C., Puchta R., Schleyer P. v. R. (2005). Chem. Rev..

[cit28] Schleyer P. v. R., Wu J. I., Cossío F. P., Fernández I. (2014). Chem. Soc. Rev..

[cit29] Townsend N. S., Green M., Russell C. A. (2012). Organometallics.

[cit30] Tate C. W., Hitchcock P. B., Lawless G. A., Benkö Z., Nyulászi L., Nixon J. F. (2010). C. R. Chim..

[cit31] Waschbüsch K., Le Floch P., Mathey F. (1996). Organometallics.

[cit32] Waschbüsch K., Le Floch P., Richard L., Mathey F. (1997). Chem. Ber..

[cit33] Roberts J. D., Lutz R. P. (1961). J. Am. Chem. Soc..

[cit34] Mallory F. B. (1973). J. Am. Chem. Soc..

[cit35] Mallory F. B., Luzik E. D., Mallory C. W., Carroll P. J. (1992). J. Org. Chem..

[cit36] Hierso J.-C. (2014). Chem. Rev..

[cit37] Hierso J.-C., Fihri A., Ivanov V. V., Hanquet B., Pirio N., Donnadieu B., Rebière B., Amardeil R., Meunier P. (2004). J. Am. Chem. Soc..

[cit38] Chalmers B. A., Arachchige K. S. A., Prentis J. K. D., Knight F. R., Kilian P., Slawin A. M. Z., Woollins J. D. (2014). Inorg. Chem..

[cit39] Taylor L. J., Surgenor B. A., Wawrzyniak P., Ray M. J., Cordes D. B., Slawin A. M. Z., Kilian P. (2016). Dalton Trans..

[cit40] Chalmers B. A., Nejman P. S., Llewellyn A. V., Felaar A. M., Griffiths B. L., Portman E. I., Gordon E.-J. L., Fan K. J. H., Woollins J. D., Bühl M., Malkina O. L., Cordes D. B., Slawin A. M. Z., Kilian P. (2018). Inorg. Chem..

[cit41] Malkina O. L., Hierso J.-C., Malkin V. G. (2022). J. Am. Chem. Soc..

[cit42] Bondi A. (1964). J. Phys. Chem..

[cit43] Barrett N., Diefenbach M., Mahon M. F., Krewald V., Webster R. L. (2022). Angew. Chem., Int. Ed..

[cit44] Cordero B., Gómez V., Platero-Prats A. E., Revés M., Echeverréa J., Cremades E., Barragán F., Alvarez S. (2008). Dalton Trans..

[cit45] Allen D. W., Taylor B. F. (1982). J. Chem. Soc., Dalton Trans..

[cit46] Dochnahl M., Doux M., Faillard E., Ricard L., Le Floch P. (2005). Eur. J. Inorg. Chem..

[cit47] Doux M., Bouet C., Mézailles N., Ricard L., Le Floch P. (2002). Organometallics.

[cit48] Budzelaar P. H. M. , gNMR, IvorySoft, 1995–2006

[cit49] Malkina O. L., Kristková A., Malkin E., Komorovský S., Malkin V. G. (2011). Phys. Chem. Chem. Phys..

[cit50] Kaupp M., Patrakov A., Reviakine R., Malkina O. L. (2005). Chem. Eur. J..

[cit51] (a) CCDC 2477493: Experimental Crystal Structure Determination, 2025, 10.5517/ccdc.csd.cc2p5142

