# Supplementary material for: Exceptionally large “through-space” nuclear spin coupling in a 2,4,6-tri(phosphanyl)–1,3,5-triphosphabenzene
Source: Chem Sci. 2025 Nov 4;17(1):544–54. doi: 10.1039/d5sc07729j (PMC12603949; doi:10.1039/d5sc07729j)
Supplement: SC-017-D5SC07729J-s001 [file SC-017-D5SC07729J-s001.pdf]

Supplementary Information (SI) for Chemical Science.
This journal is © The Royal Society of Chemistry 2025

# Supporting Information

## Contents

1. NMR spectra ....2
1.1. NMR spectra of compound **1** ....2
1.2. NMR spectra of compound **2** ....5
1.3. NMR spectra of compound **4** ....8
1.4. NMR spectra of compound **5** ....11
1.5. *In Situ* NMR experiments ....15
1.6. Coupling constants from simulated spectra ....16
2. High resolution mass data ....18
3. X-ray crystallographic studies ....21
4. Computational details ....36
4.1 General ....36
4.2 Nuclear magnetic resonance DFT simulations ....36
4.3 Cartesian coordinates ....38
5. References ....74

## 1. NMR spectra

1.1. NMR spectra of compound **1**

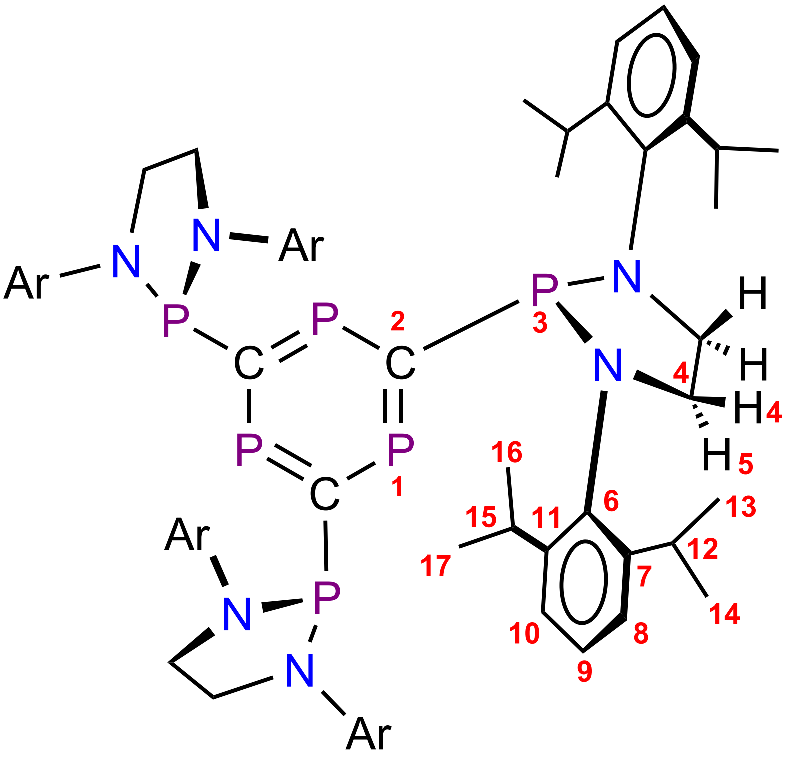

**Figure S1.** Compound **1** with the atom labelling used for the assignment of NMR spectra.

**$^{1}$H NMR (500 MHz, $C_6D_6$):** δ (ppm) 7.18 (dd, $^{3}J_{H–H}$ = 7.7 Hz, $^{4}J_{H–H}$ = 1.7 Hz, 6H; $H_{10}$, partially overlapped with $C_6D_6$ resonance), 7.12 (t, $^{3}J_{H–H}$ = 7.7 Hz, 6H; $H_9$), 6.83 (dd, $^{3}J_{H–H}$ = 7.7 Hz, $^{4}J_{H–H}$ = 1.7 Hz, 6H; $H_8$), 4.13–4.01 (m, 12H; $H_{4\text{ or }5,12}$), 3.55 (sept, $^{3}J_{H–H}$ = 6.8 Hz, 6H; $H_{15}$), 3.31–3.22 (m, 6H; $H_{4\text{ or }5}$), 1.67 (d, $^{3}J_{H–H}$ = 6.8 Hz, 18H; $H_{14}$), 1.34 (d, $^{3}J_{H–H}$ = 6.8 Hz, 18H; $H_{13}$), 1.10 (d, $^{3}J_{H–H}$ = 6.8 Hz, 18H, $H_{16}$), –0.28 (d, $^{3}J_{H–H}$ = 6.8 Hz, 18H; $H_{17}$).

**$^{13}$C{$^{1}$H} NMR (126 MHz, $C_6D_6$):** δ (ppm) 149.40 (d, $J_{C–P}$ = 2.2 Hz; $C_7$), 148.04 (s; $C_{11}$), 139.11 (d, $J_{C–P}$ = 14.7 Hz; $C_6$), 127.01 (s; $C_9$), 124.80 (s; $C_{10}$), 124.39 (s; $C_8$), 56.94 (d, $J_{C–P}$ = 5.9 Hz;

$C_{4,5}$), 29.35 (d, $J_{C–P}$ = 14.2 Hz; $C_{12}$), 28.39 (br; $C_{15}$), 25.71 (s; $C_{13}$), 25.41 ($C_{16}$), 25.02 (d, $J_{C–P}$ = 4.6 Hz; $C_{14}$), 24.83 (s; $C_{17}$).

**$^{31}$P NMR (202 MHz, $C_6D_6$):** δ (ppm) 295.48 (d, $^{TS}J_{P–P}$ = 432 Hz; $P_1$), 118.46 (d, $^{TS}J_{P–P}$ = 432 Hz; $P_3$).

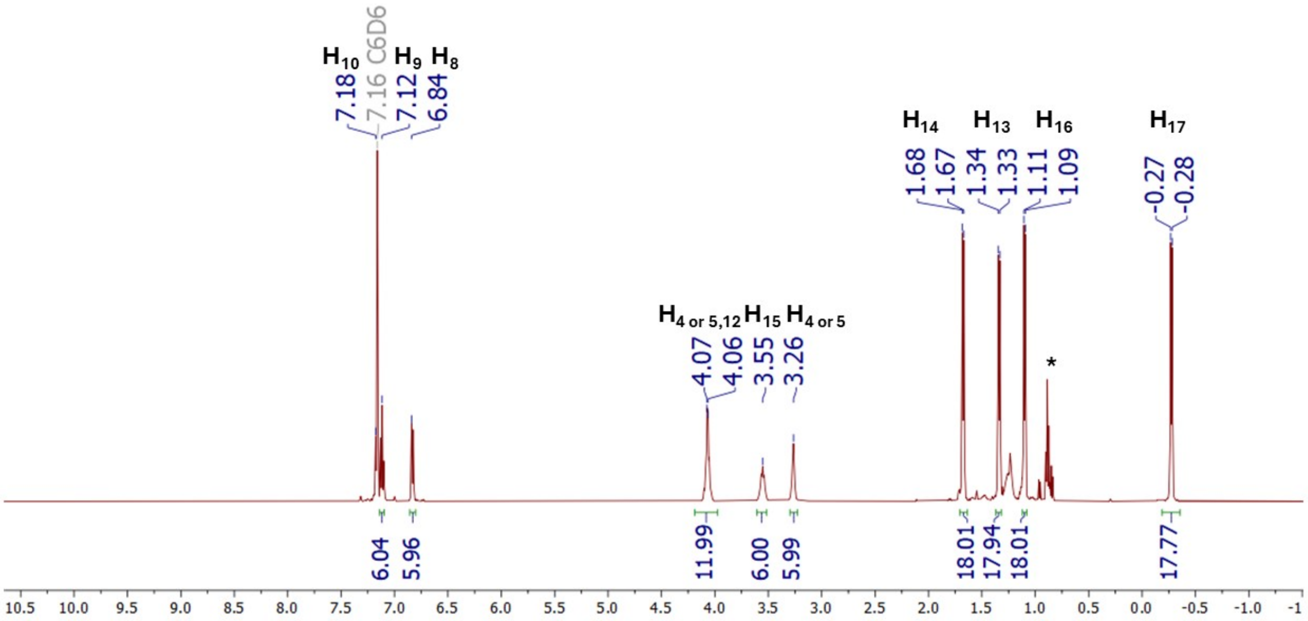


**Figure S2.** $^1$H NMR (298 K, $C_6D_6$, 500 MHz) spectrum of **1**. Note: Residual pentane resonance*.

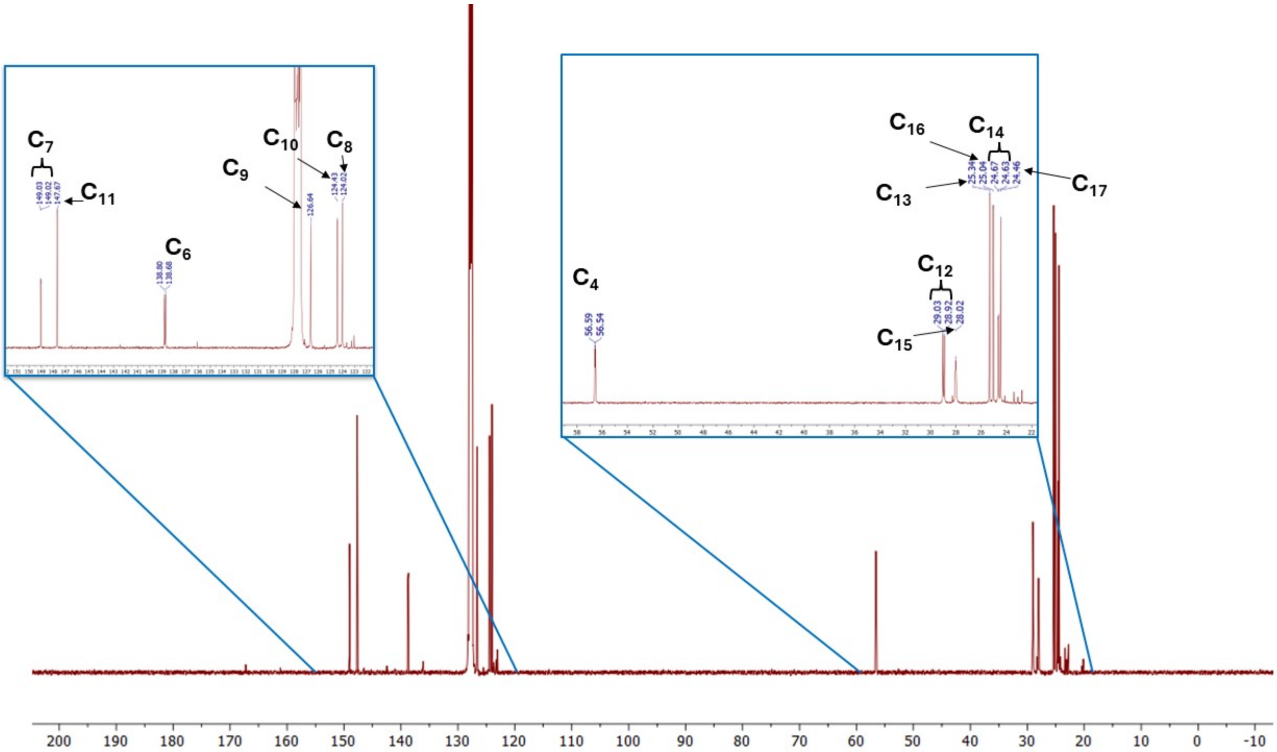


**Figure S3.** $^{13}C\{^{1}H\}$ NMR (298 K, $C_6D_6$, 126 MHz) spectrum of **1**.

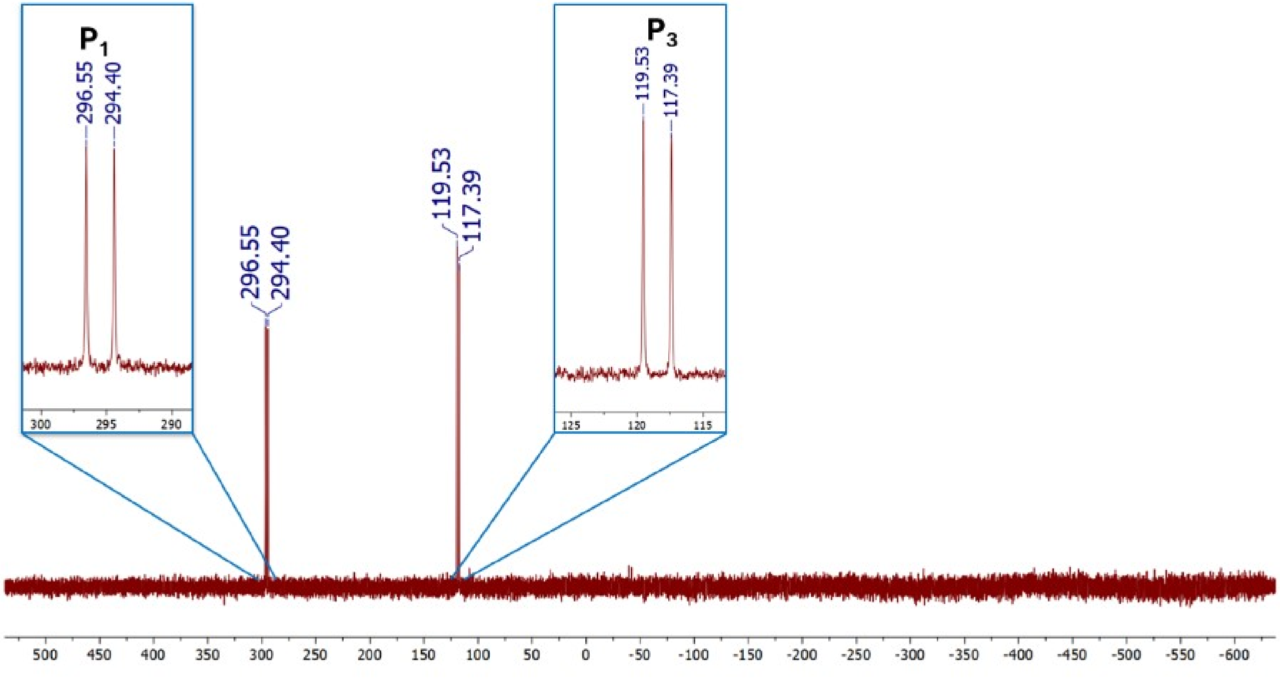


**Figure S4.** $^{31}P\{^{1}H\}$ NMR (298 K, $C_6D_6$, 202 MHz) spectrum of **1**.

1.2. NMR spectra of compound **2**

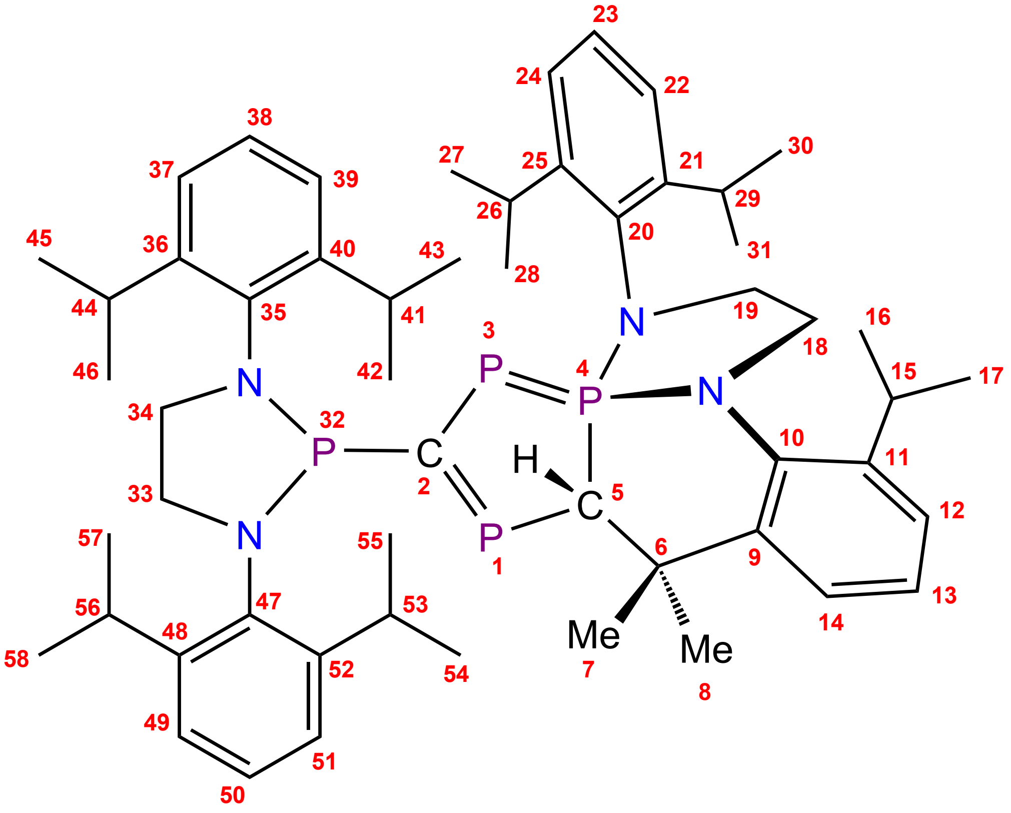


**Figure S5.** Compound **2** with the atom labelling used for the assignment of NMR spectra.

**$^1$H NMR (500 MHz, $C_6D_6$)** δ 7.26 (t, $^3J_{H–H}$ = 7.7 Hz, 1H, $H_{23}$), 7.23 – 7.05 (m, 8H, $H_{22,24,37,38, 39, 49, 50, 51}$), 6.82 (dd, $^3J_{H–H}$ = 7.7 Hz, $^4J_{H–H}$ = 1.7 Hz, 1H, $H_{14}$), 6.80 (dd, $^3J_{H–H}$ = 7.7 Hz, $^4J_{H–H}$ = 1.7 Hz, 1H, $H_{12}$), 6.73 (t, $^3J_{H–H}$ = 7.7 Hz, 1H, $H_{13}$) 4.70 (s, br, 1H, $H_{53}$), 4.12 – 4.02 (m, 2H, $H_{33,19}$), 4.02–3.94 (m, 1H, $H_{34}$), 3.89 (br, 1H, $H_{41}$), 3.84 (br, 1H, $H_{56}$), 3.77 (sept, $^3J_{H–H}$ = 6.7 Hz, 1H, $H_{26}$), 3.63–3.50 (m, 3H, $H_{5,18,44}$), 3.36 (sept, $^3J_{H–H}$ = 6.7 Hz, 1H, $H_{29}$), 3.32–3.25 (m, 1H, $H_{33}$), 3.22–3.12 (m, 2H, $H_{15,34}$), 3.00–2.91 (m, 1H, $H_{18}$), 2.72 – 2.63 (m, 1H, $H_{19}$), 1.75 – 1.69 (m, 6H, $H_{7,30}$), 1.55 (s, 3H, $H_8$), 1.49 (s, br, 6H, $H_{54,57}$), 1.39 (s, br, 3H, $H_{43}$), 1.32 (d, $^3J_{H–H}$ = 6.7 Hz, 3H, $H_{28}$), 1.25 (s, br, 9H, $H_{54,57,42}$), 1.16 – 1.01 (m, 15H, $H_{16,17,27,31,45}$), 0.67 (s, br, 3H, $H_{46}$).

**$^{13}C\{^{1}H\}$ NMR (126 MHz, $C_6D_6$)** δ 150.49 (m), 149.73 (m), 149.37 (d, $J_{C–P}$ = 1.3 Hz; $C_{21}$), 148.94 (d, $J_{C–P}$ = 1.8 Hz; $C_{25}$), 148.39 (m), 147.97 (m), 144.79 (d, $J_{C–P}$ = 5.1 Hz; $C_{11}$), 142.87 (d, $J_{C–P}$ = 3.8 Hz; $C_{10}$), 140.83, 140.71, 140.55 (d, $J_{C–P}$ = 3.9 Hz; $C_9$), 140.07, 139.96, 136.58 (d, $J_{C–P}$ = 5.8 Hz; $C_{20}$), 126.67, 126.46, 126.06 ($C_{12}$), 125.66 ($C_{13}$), 125.19 ($C_{22}$), 124.74 ($C_{23}$), 124.27 ($C_{24}$), 123.13 ($C_{14}$), 65.95 (ddd, $J_{C–P}$ = 62.5, 31.0, 14.9 Hz, $C_5$), 56.44 (d, $J_{C–P}$ = 5.0 Hz; $C_{33}$), 55.72 (d, $J_{C–P}$ = 7.1 Hz; $C_{34}$), 51.26 (d, $J_{C–P}$ = 6.5 Hz; $C_{18}$), 50.92 (d, $J_{C–P}$ = 4.2 Hz; $C_{19}$), 38.63 (d, $J_{C–P}$ = 4.4 Hz; $C_6$), 33.94 (dd, $J_{C–P}$ = 20.2, 6.9 Hz; $C_7$), 32.92 ($C_8$), 29.14 ($C_{29,56}$), 29.04 ($C_{29,56}$), 28.80 ($C_{15,41}$), 28.56 ($C_{26,44}$), 28.37 ($C_{53}$), 26.86, 26.70 ($C_{27,31}$), 26.57 ($C_{27,31}$), 26.21, 25.97, 25.75, 25.49 ($C_{16,17}$), 25.32 ($C_{16,17}$), 24.48, 24.12 (dd, $J_{C–P}$ = 16.3, 3.0 Hz; $C_{30}$), 22.89 ($C_{28}$).

**$^{31}P$ NMR (202 MHz, $C_6D_6$)** δ 242.01 (d, $J$ = 415.8 Hz, $P_1$), 127.26 (dt, $J$ = 559.7, 18.6 Hz, $P_4$), 107.04 (dd, $J$ = 415.8, 18.6 Hz, $P_{32}$), -40.63 (d, $J$ = 559.7 Hz; $P_3$).

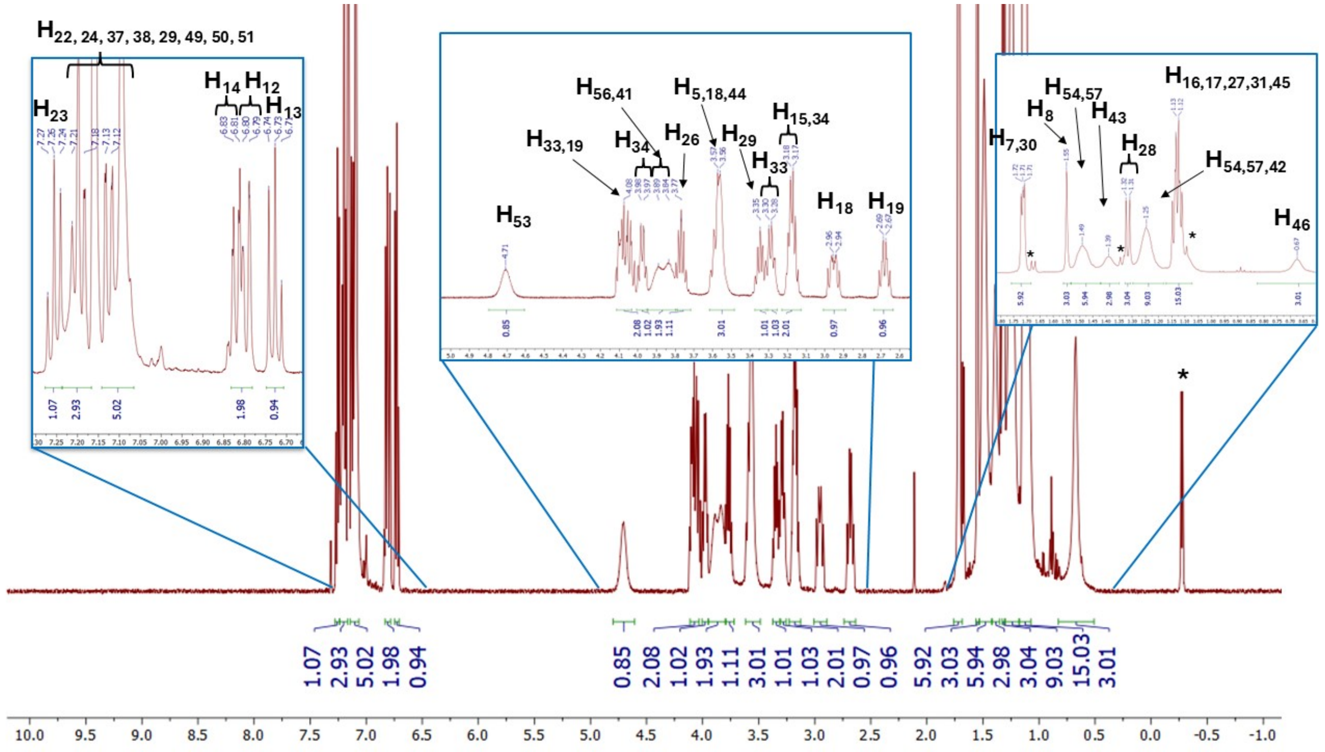


**Figure S6.** $^{1}H$ NMR (298 K, $C_6D_6$, 500 MHz) spectrum of **2**. *Residual peaks of **1.**

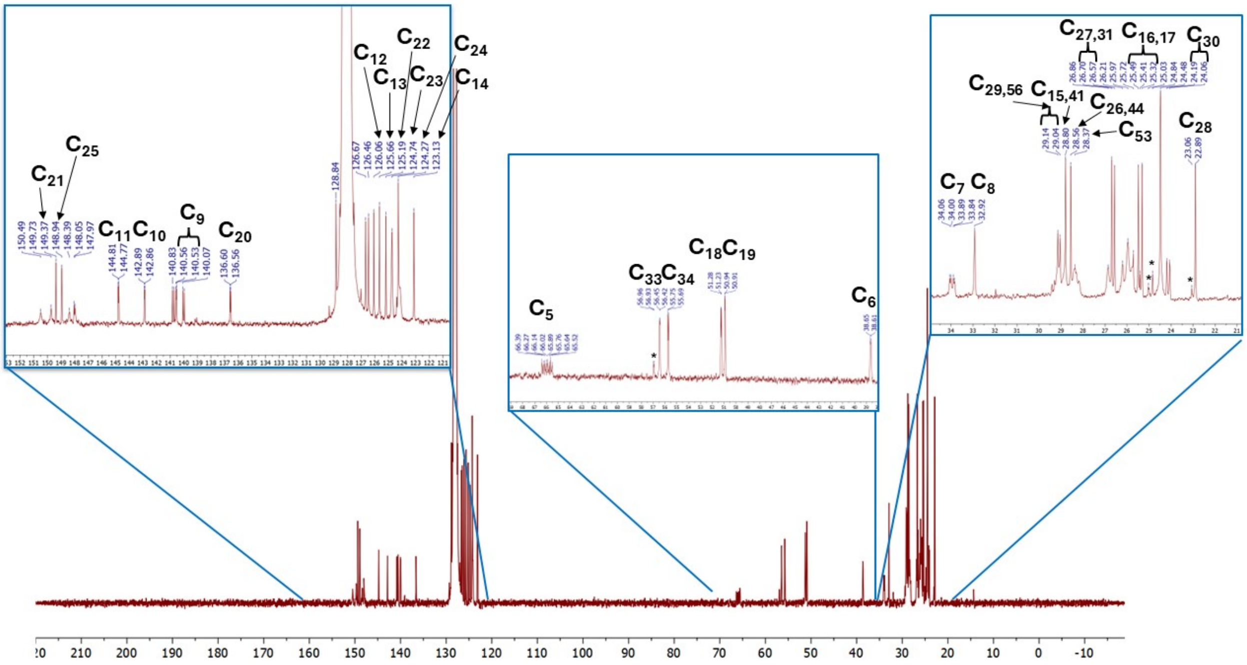


**Figure S7.** $^{13}C\{^1H\}$ NMR (298 K, $C_6D_6$, 500 MHz) spectrum of **2**. *Residual peaks of **1**.

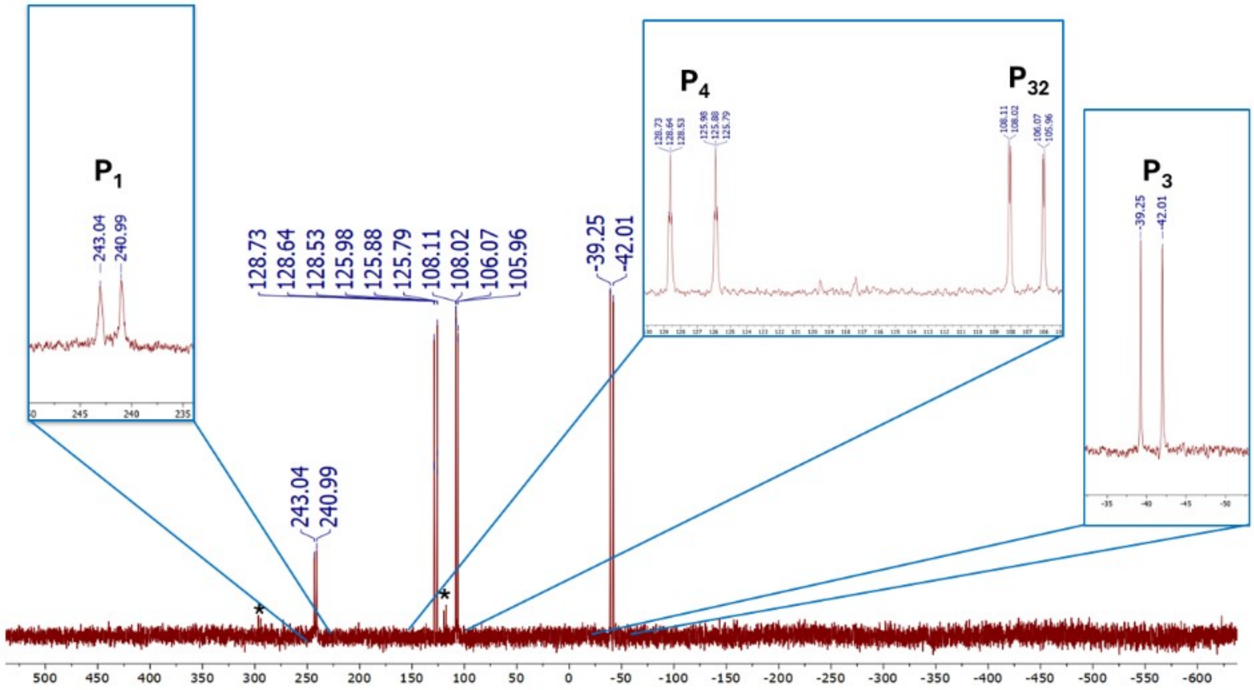


**Figure S8.** $^{31}P\{^1H\}$ NMR (298 K, $C_6D_6$, 202 MHz) spectrum of **2.** *Residual peaks of **1**.

1.3. NMR spectra of compound **4**

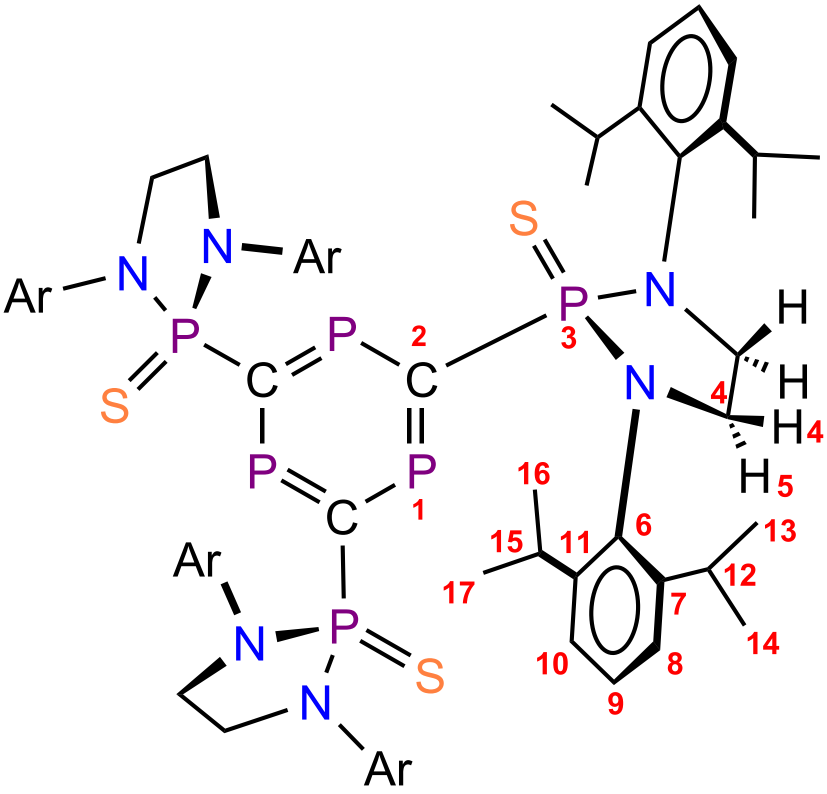


**Figure S9.** Compound **4** with the atom labelling used for the assignment of NMR spectra.

**$^1$H NMR (500 MHz, $C_6D_6$):** δ (ppm) 7.23 (dd, $^3J_{H–H}$ = 7.7 Hz, $^4J_{H–H}$ = 1.7 Hz, 6H; $H_{10}$), 7.17 (t, $^3J_{H–H}$ = 7.7 Hz, 6H; $H_9$), 6.91 (dd, $^3J_{H–H}$ = 7.7 Hz, $^4J_{H–H}$ = 1.7 Hz, 6H; $H_8$), 4.43 (sept, $^3J_{H–H}$ = 6.7 Hz, 6H; $H_{12}$), 4.04–3.92 (m, 6H; $H_{4\text{ or }5}$), 3.57–3.45 (m, 6H; $H_{4\text{ or }5}$), 3.43–3.30 (m, 6H; $H_{15}$), 1.71 (d, $^3J_{H–H}$ = 6.7 Hz, 18H; $H_{14}$), 1.19 (d, $^3J_{H–H}$ = 6.7 Hz, 18H; $H_{13}$), 1.01 (d, $^3J_{H–H}$ = 6.7 Hz, 18H; $H_{16}$), 0.22 (d, $^3J_{H–H}$ = 6.7 Hz, 18H; $H_{17}$).

**$^{13}$C{$^1$H} NMR (126 MHz, $C_6D_6$):** δ (ppm) 150.47 (s; $C_{11}$), 149.69 (d, $J_{C–P}$ = 2.7 Hz; $C_7$), 135.90 (d, $J_{C–P}$ = 4.6 Hz; $C_6$), 128.4–127.1 ($C_9$ overlapped with $C_6D_6$ resonance), 124.60 (s; $C_{10}$), 124.35

(s; $C_8$), 53.51 (d, $J_{C–P}$ = 8.3 Hz; $C_{4,5}$), 29.76 (s; $C_{12}$), 29.16 (m; $C_{15}$), 26.79 (s; $C_{13}$), 26.23 (s; $C_{16}$), 23.95 (s; $C_{14}$), 23.76 (s; $C_{17}$).

**$^{31}$P NMR (202 MHz, $C_6D_6$):** δ(ppm) 291.04 (dd, $^2J_{P–P}$ = 147, 106 Hz; $P_1$), 71.87 (dd, $^2J_{P–P}$ = 147, 106 Hz, $P_3$).

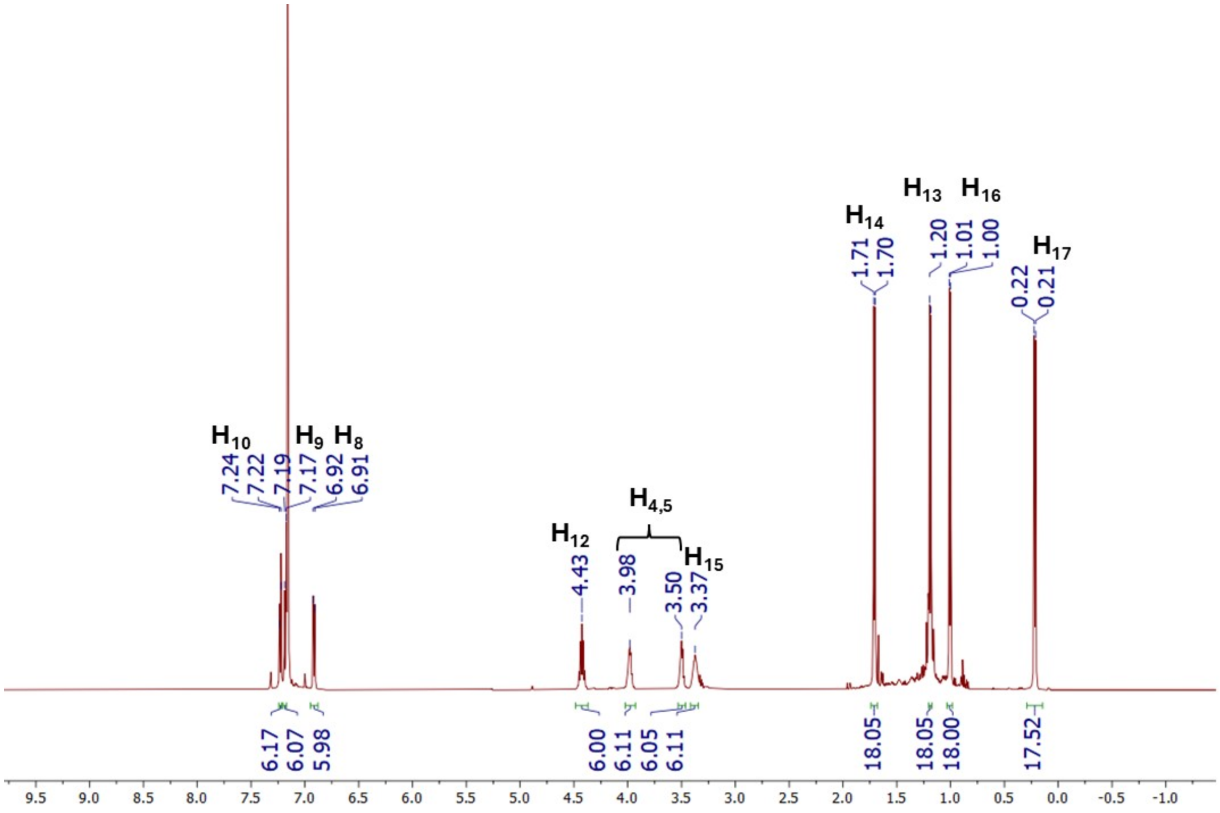


**Figure S10.** $^1$H NMR (298 K, $C_6D_6$, 500 MHz) spectrum of **4**.

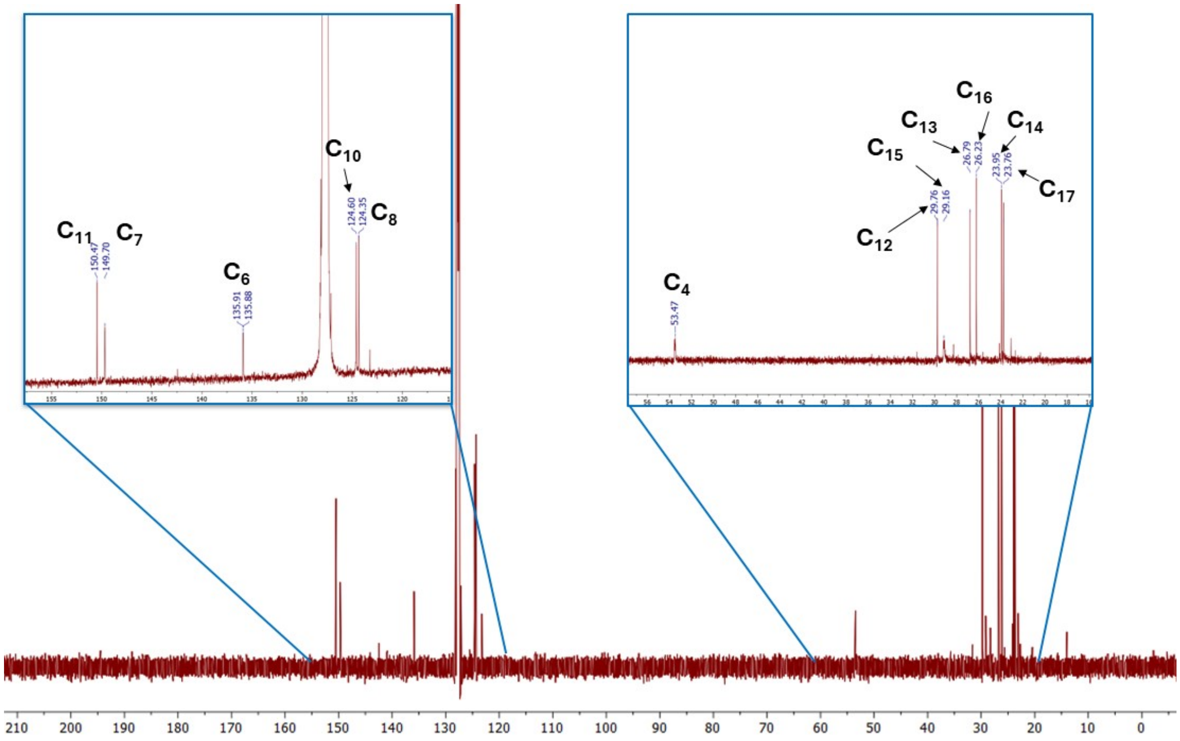


**Figure S11.** $^{13}C\{^{1}H\}$ NMR (298 K, $C_6D_6$, 126 MHz) spectrum of **4**.

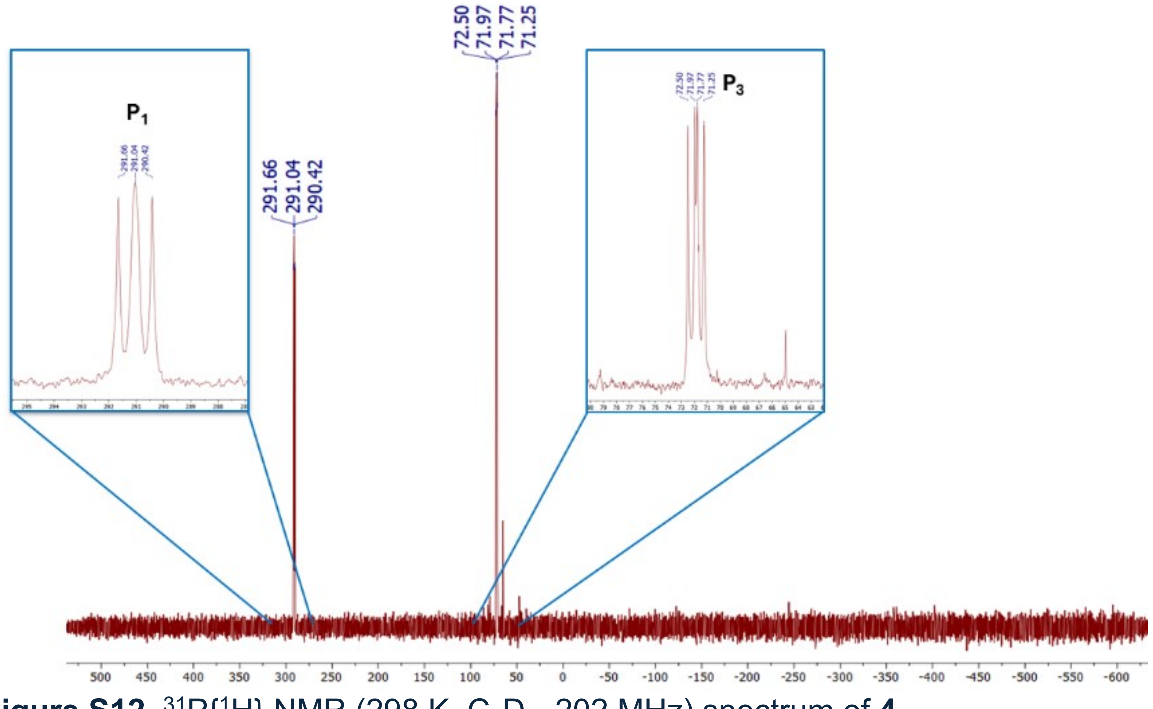


**Figure S12.** $^{31}P\{^{1}H\}$ NMR (298 K, $C_6D_6$, 202 MHz) spectrum of **4**.

1.4. NMR spectra of compound **5**

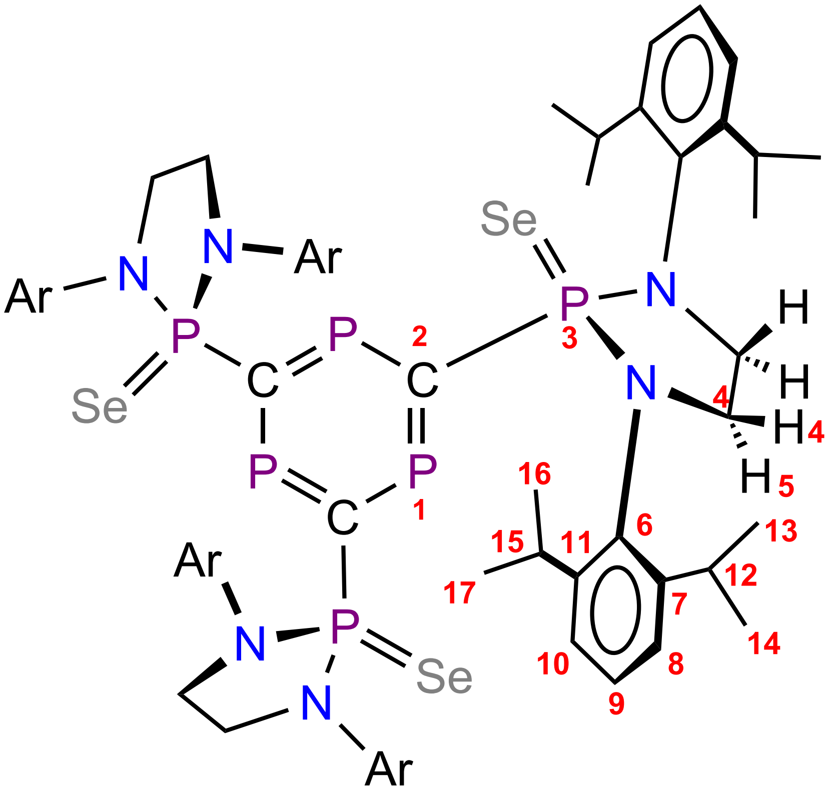


**Figure S13.** Compound **5** with the atom labelling used for the assignment of NMR spectra.

**$^1$H NMR (500 MHz, $C_6D_6$):** δ (ppm) 7.23 (dd, $^3J_{H–H}$ = 7.6 Hz, $^4J_{H–H}$ = 1.8 Hz, 6H; $H_{10}$), 7.19 (t, $^3J_{H–H}$ = 7.6 Hz, 6H; $H_9$), 6.96 (dd, $^3J_{H–H}$ = 7.6 Hz, $^4J_{H–H}$ = 1.8 Hz, 6H; $H_8$), 4.35 (sept, $^3J_{H–H}$ = 6.8 Hz, 6H; $H_{12}$), 4.08–3.93 (m, 6H; $H_{4\text{ or }5}$), 3.55–3.42 (m, 6H; $H_{4\text{ or }5}$), 3.37–3.20 (m, 6H; $H_{15}$), 1.71 (d, $^3J_{H–H}$ = 6.8 Hz, 18H; $H_{14}$), 1.18 (d, $^3J_{H–H}$ = 6.8 Hz, 18H; $H_{13}$), 0.99 (d, $^3J_{H–H}$ = 6.8 Hz, 18H; $H_{16}$), 0.37 (d, $^3J_{H–H}$ = 6.8 Hz, 18H; $H_{17}$).

**$^{13}$C{$^1$H} NMR (126 MHz, $C_6D_6$):** δ (ppm) 187.19 (dd, $J_{C–P}$ = 167.5, 95.1 Hz; $C_2$), 150.72 (s; $C_{11}$), 150.08 (d, $J_{C–P}$ = 3.0 Hz; $C_7$), 136.59 (d, $J_{C–P}$ = 5.0 Hz; $C_6$), 128.4–127.1 ($C_9$ overlapped with

$C_6D_6$ resonance), 125.06 (s; $C_{10}$), 124.77 (s; $C_8$), 54.53 (d, $J_{C–P}$ = 8.2 Hz; $C_{4,5}$), 30.22 (s; $C_{12}$), 29.63 (d, $J_{C–P}$ = 8.9 Hz; $C_{15}$), 27.23 (s; $C_{13}$), 26.71 (s; $C_{16}$), 24.40 (s; $C_{14}$), 24.22 (s; $C_{17}$).

**$^{31}$P NMR (202 MHz, $C_6D_6$):** δ (ppm) 284.90 (dd, $^2J_{P–P}$ = 168, 101 Hz; $P_1$), 70.68 (dd, $^2J_{P–P}$ = 168, 101 Hz, satellites $^1J_{P–Se}$ = 803 Hz; $P_2$).

**$^{77}$Se NMR (95 MHz, $C_6D_6$):** δ 21.89 (dd, $J$ = 803, 103 Hz).

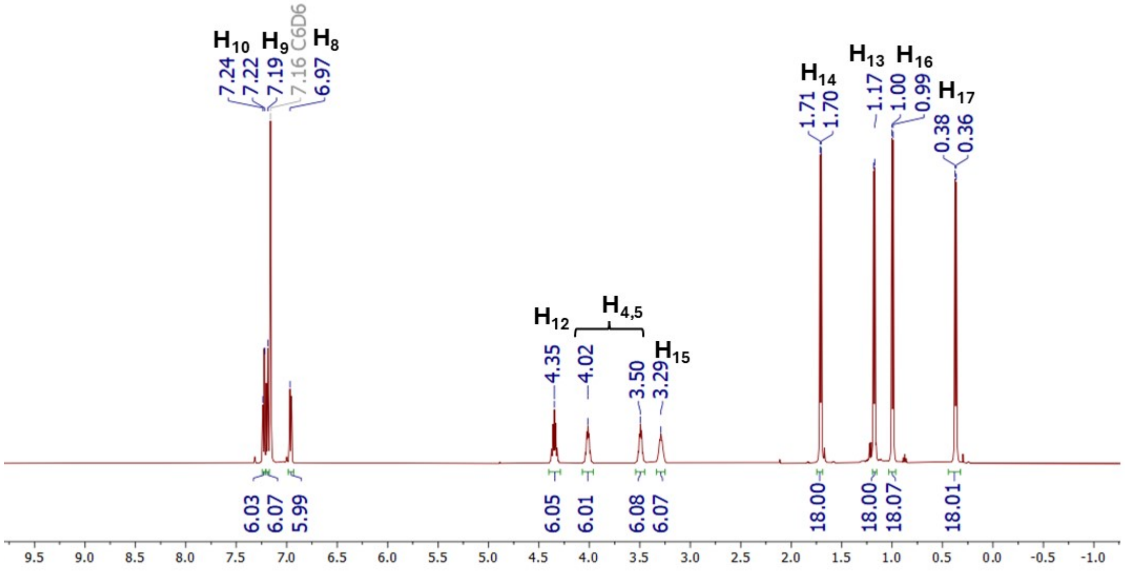


**Figure S14.** $^1$H NMR (298 K, $C_6D_6$, 500 MHz) spectrum of **5**.

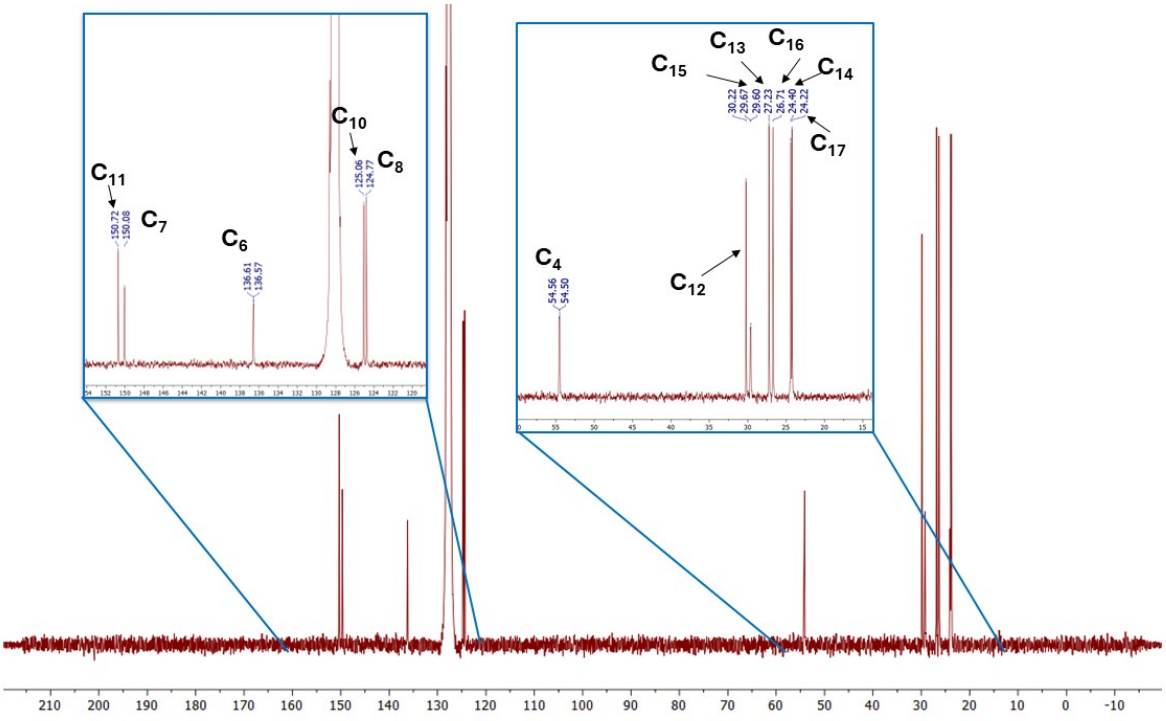


**Figure S15.** $^{13}C\{^1H\}$ NMR (298 K, $C_6D_6$, 500 MHz) spectrum of **5**.

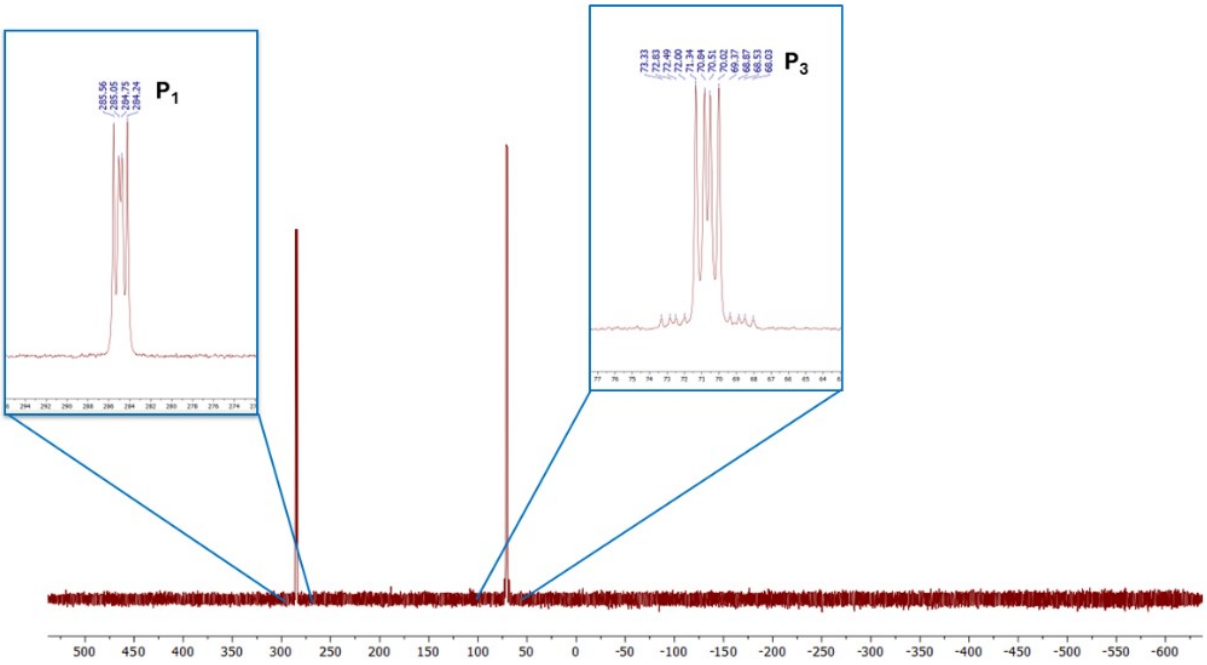


**Figure S16.** $^{31}P\{^1H\}$ NMR (298 K, $C_6D_6$, 202 MHz) spectrum of **5**.

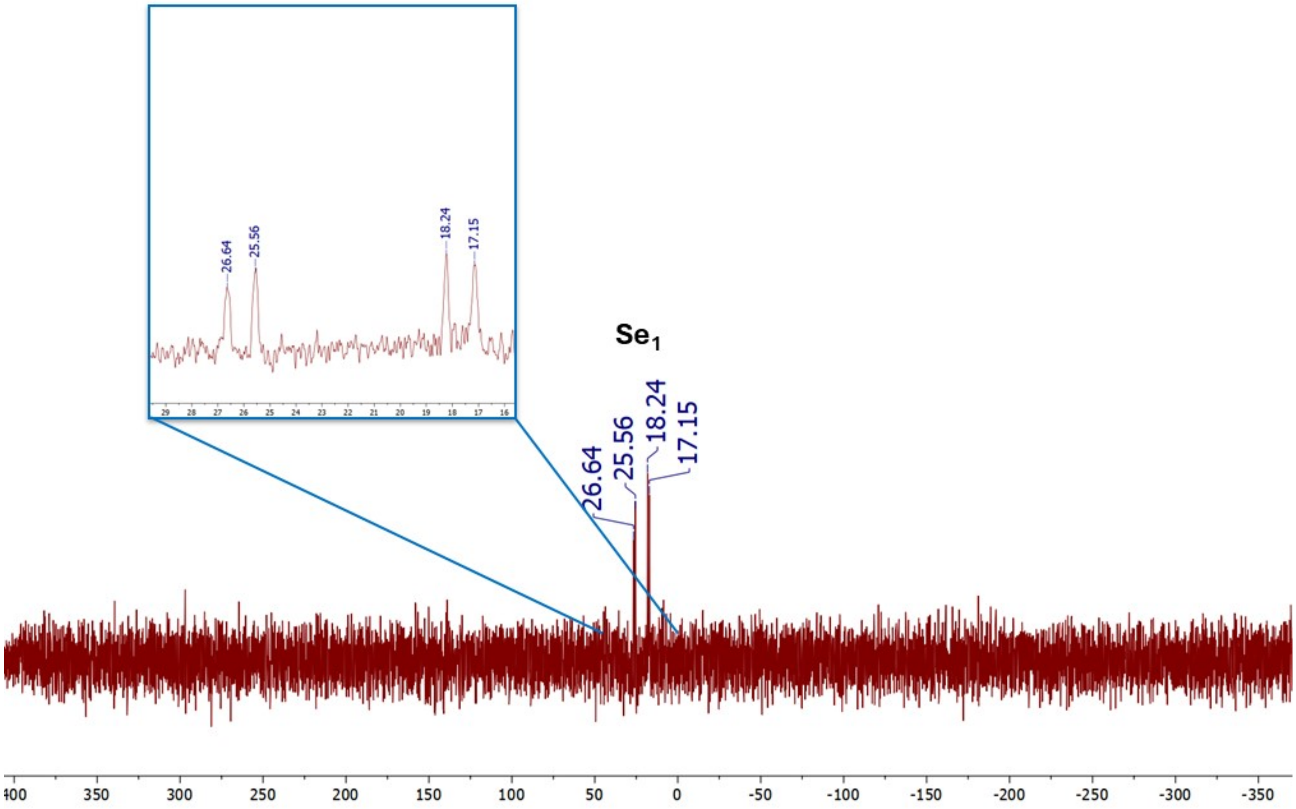


**Figure S17.** $^{77}Se\{^1H\}$ NMR (298 K, $C_6D_6$, 95 MHz) spectrum of **5**.

1.5. *In Situ* NMR experiments

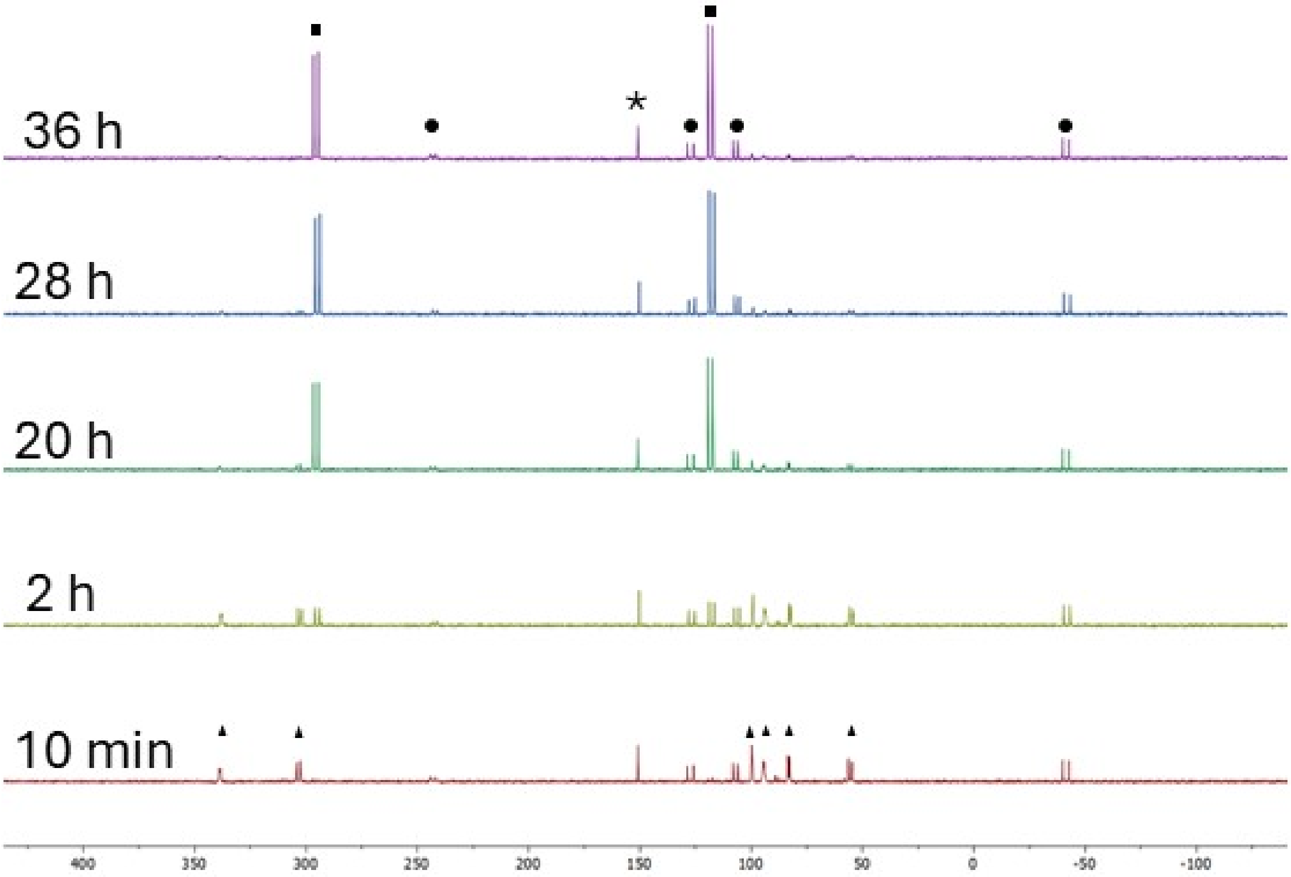


**Figure S18.** $^{31}P$ NMR (298 K, $C_6D_6$, 202 MHz) stacked spectra of the evolution overtime of the reaction of $[(H_2C)_2(NDipp)_2]PCl$ with half of an equivalent of $\{[Mg]CP\}_2$. Note: Intermediate **3** (▲); product **1** (■); product **2** (●)**.** *Residual starting material $(H_2C)_2(NDipp)_2]PCl$.

**Figure S19.** $^{31}P$ NMR (298 K, $C_6D_6$, 202 MHz) stacked spectra of the evolution overtime of compound **3** (▲) in benzene solution to yield product **1** (■). *Residual starting material $(H_2C)_2(NDipp)_2]PCl$.

1.6. Coupling constants from simulated spectra

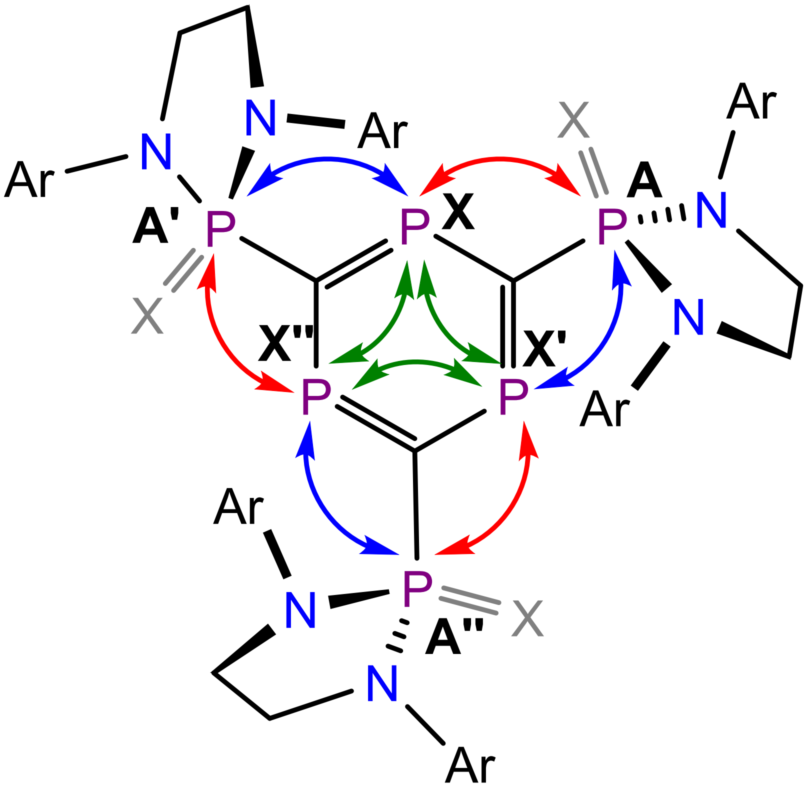


| $^2J(^{31}P–^{31}P)$ | **1** (X = L.P.) | **4** (X= S) | **5** (X = Se) |
|---|---|---|---|
| $^2J_{A–X}$ | 432 *(346.5)* | 147 | 168 |
| $^2J_{A–X'}$ | 0 *(13.2)* | 106 | 101 |
| $^2J_{X–X'}$ | 4 *(12.4)* | 18 | 20 |

**Table S1.** Pattern of $^2J(^{31}P–^{31}P)$ couplings (Hz) in **1**, **4** and **5**. Values in parentheses for **1** are the computed coupling constants as determined by DFT (ωB97x-D3BJ/def2-TZVPP//ωB97x-D3BJ/def2-SVP level).

## 2. High resolution mass data

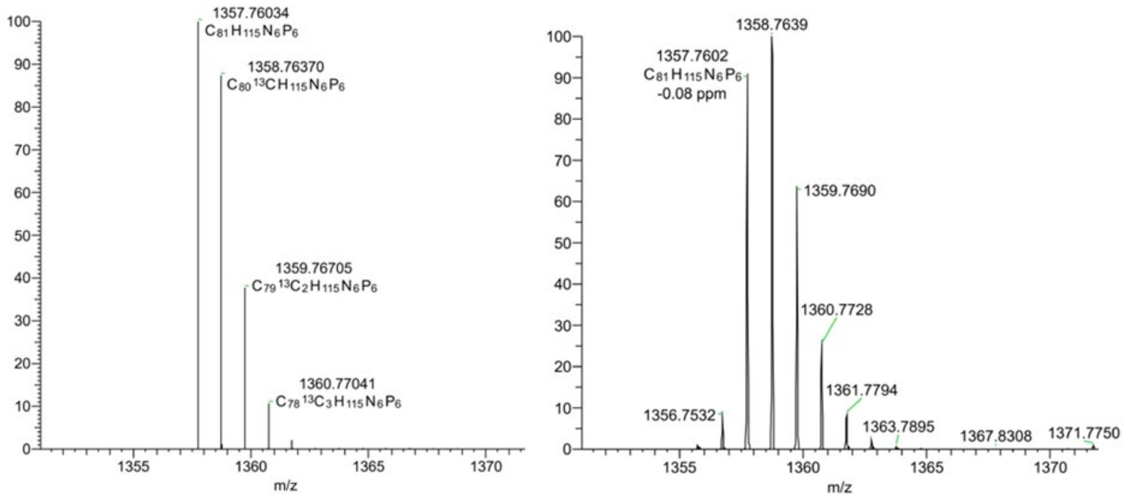


**Figure S20.** Right: HR-MS (APCI-Orbitrap; positive-ion mode) of **1** showing the $[M+H]^+$ peak at *m*/*z* 1357.7602 (calcd 1357.76034; –0.08 ppm error); Left: simulation.

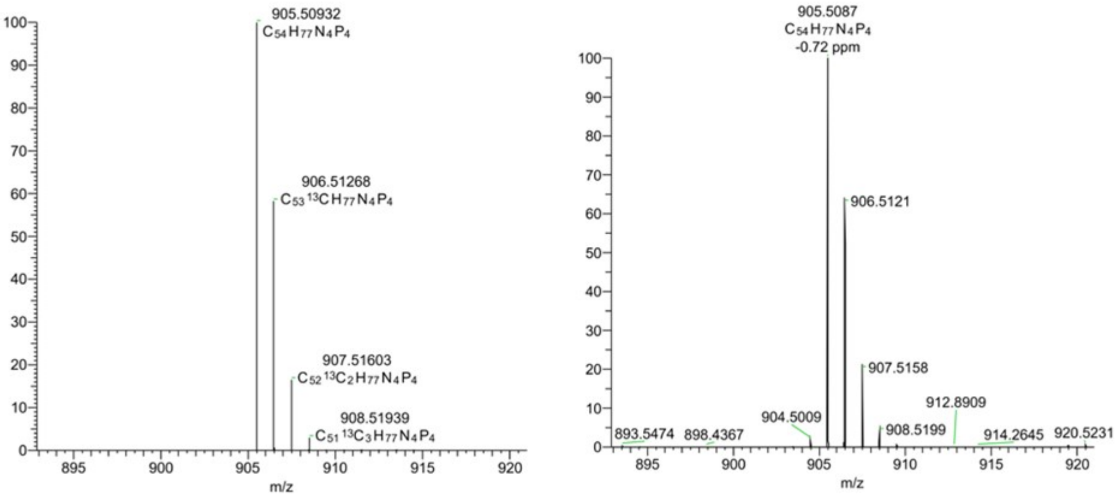


**Figure S21.** Right: HR-MS (APCI-Orbitrap; positive-ion mode) of **2** showing the $[M+H]^+$ peak at *m*/*z* 905.5087 (calcd 905.50932; –0.72 ppm error); Left: simulation.

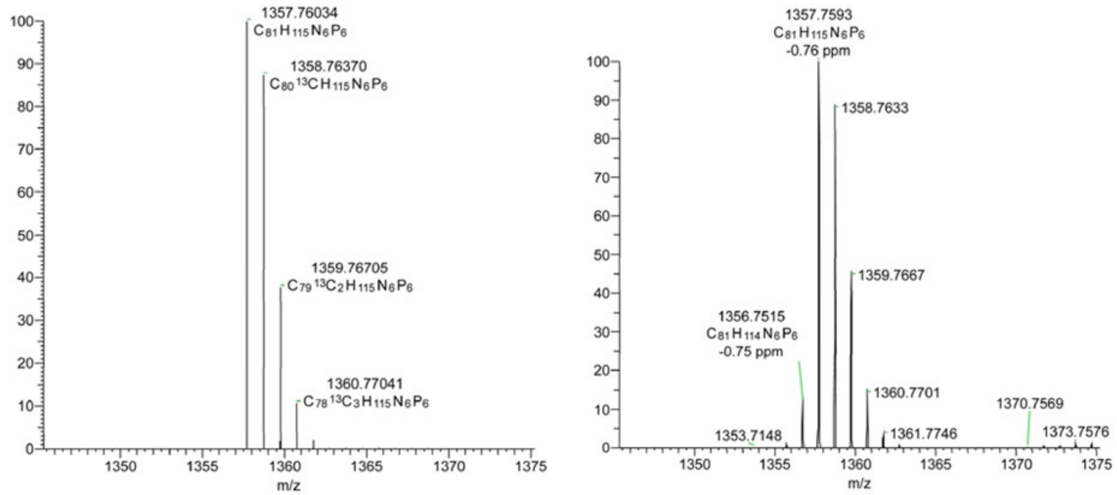


**Figure S22.** Right: HR-MS (APCI-Orbitrap; positive-ion mode of **3** showing the $[M+H]^+$ peak at *m*/*z* 1357.7593 (calcd 1357.76034; –0.76 ppm error); Left: simulation.

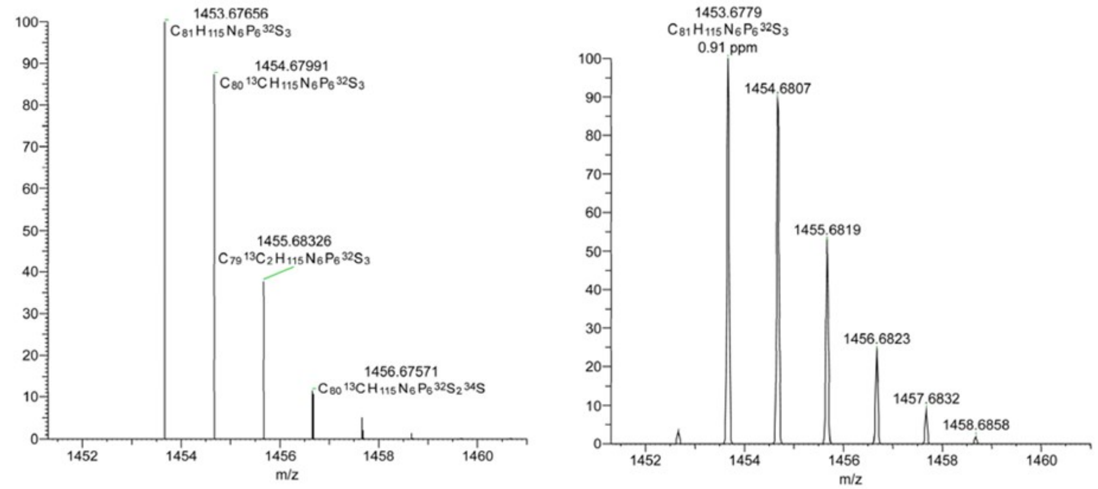


**Figure S23.** Right: HR-MS (APCI-Orbitrap; positive-ion mode) of **4** showing the $[M+H]^+$ peak at *m*/*z* 1453.6779 (calcd 1453.67656; 0.91 ppm error); Left: simulation.

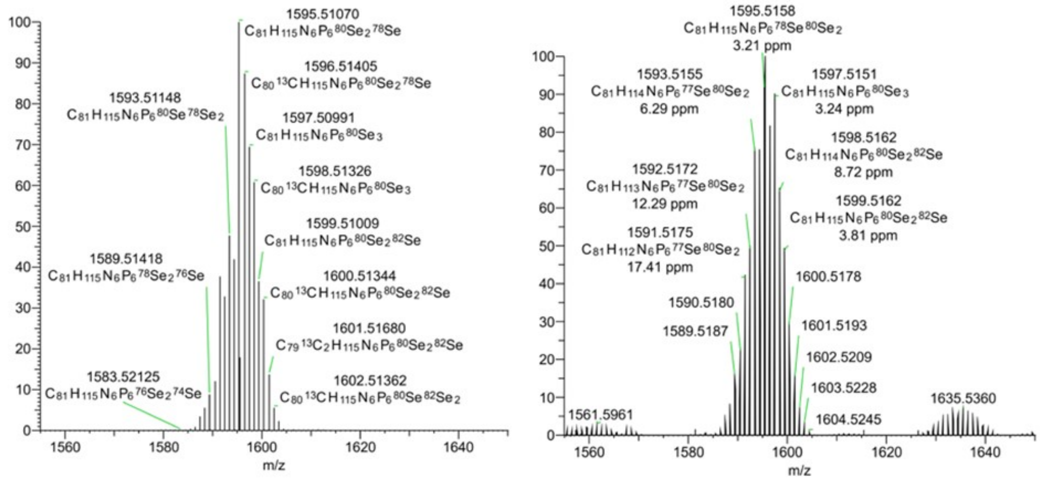


**Figure S24.** Right: HR-MS (APCI-Orbitrap; positive-ion mode) of **5** showing the $[M+H]^+$ peak at *m*/*z* 1453.6779 (calcd 1453.67656; 0.91 ppm error); Left: simulation.

## 3. X-ray crystallographic studies

**Table S2.** Crystal data and structure refinement for **1**·2hex (CCDC 2477493).

| | | |
|---|---|---|
| Empirical formula | $C_{93}H_{142}N_6P_6$ | |
| Formula weight | 1529.94 | |
| Crystal color, shape, size | red needle, 0.020 × 0.092 × 0.367 $mm^3$ | |
| Temperature | 173(2) K | |
| Wavelength | 1.54178 Å (Cu Kα) | |
| Crystal system, space group | Monoclinic, *C2/c* | |
| Unit cell dimensions | *a* = 48.6560(10) Å | α = 90° |
| | *b* = 15.8779(3) Å | β = 93.165(2)° |
| | *c* = 48.7057(9) Å | γ = 90° |
| Volume | 37570.4(13) $Å^3$ | |
| *Z* | 16 | |
| Density (calculated) | 1.082 $mg/m^3$ | |
| Absorption coefficient | 1.396 $mm^{-1}$ | |
| *F*(000) | 13312 | |
| ***Data collection*** | | |
| Diffractometer | Venture D8, Bruker | |
| Source | Iμ3.0, Incoatec | |
| Detector | Photon III | |
| Theta range for data collection | 1.814 to 68.38° | |
| Index ranges | $-58 \le h \le 58$, $-19 \le k \le 19$, $-58 \le l \le 58$ | |
| Reflections collected | 326494 | |
| Independent reflections | 34464 [$R_{int}$ = 0.1767] | |
| Observed Reflections [I > 2σ(I)] | 25179 | |
| Completeness to theta = 67.679° | 100% | |
| ***Solution and Refinement*** | | |
| Absorption correction | Multi-scan | |
| Max. and min. transmission | 0.753 and 0.449 | |
| Solution | Intrinsic methods | |
| Refinement method | Full-matrix least-squares on $F^2$ | |
| Weighting scheme | $w = [\sigma^2 Fo^2 + AP^2 + BP]^{-1}$, with | |
| | $P = (F_o^2 + 2 F_c^2)/3$, A = 0.0627, B = 25.89 | |
| Data / restraints / parameters | 34464 / 0 / 1675 | |
| Goodness-of-fit on $F^2$ | 1.019 | |
| Final *R* indices [$I$>2σ(I)] | $R_1$ = 0.0553, $wR_2$ = 0.1314 | |
| *R* indices (all data) | $R_1$ = 0.0805, $wR_2$ = 0.1463 | |
| Largest diff. peak and hole | 0.75 and –0.36 e·$Å^{-3}$ | |

Goodness-of-fit = $[\Sigma[w(F_o^2 - F_c^2)^2]/N_{observns} - N_{params})]^{1/2}$, all data. $R_1 = \Sigma(|F_o| - |F_c|) / \Sigma |F_o|$. $wR_2 = [\Sigma[w(F_o^2 - F_c^2)^2] / \Sigma [w(F_o^2)^2]]^{1/2}$.

## Crystal structure data for 1·2hex

A clear red, needle-shaped specimen of **1**·2hex ($C_{93}H_{142}N_6P_6$; IUMSC 24117), approximate dimensions 0.020 × 0.092 × 0.367 $mm^3$, was placed on a Kapton mount with inert oil for crystal structure determination. The X-ray intensity data were measured at 173(2) K on a Bruker D8 Venture Kappa diffractometer equipped with a microfocus sealed tube (λ = 1.54178 Å) and a multilayer mirror monochromator.

## Data collection

The data collection was carried out at 173 K using Cu Kα radiation using 1° ω and φ scans with a frame time of 4, 7 and 20 seconds and a detector distance of 4.00 cm. Overall, 2628 frames were collected with a total exposure time of 10.87 hours. The frames were integrated with the SAINT V8.41 package using a narrow-frame algorithm.[1] The integration of the data using a monoclinic unit cell yielded 326494 reflections to a maximum θ angle of 68.51° (0.83 Å resolution), of which 34464 were independent (average redundancy 9.47, completeness = 100.0%, $R_{int}$ = 17.67%, $R_{sig}$ = 7.75%) and 25179 (73.1%) were greater than 2σ($F^2$). The final cell constants of $a$ = 48.6560(10) Å, $b$ = 15.8779(3) Å, $c$ = 48.7057(9) Å, α = 90°, β = 93.165(2)°, γ = 90°, volume = 37570.4(13) Å$^3$, are based upon the refinement of the XYZ-centroids of 9906 reflections above 20 σ($I$) with 3.08° < 2θ < 68.31°. Data were corrected for absorption effects using the Multi-Scan method in SADABS 2016/2. The calculated minimum and maximum transmission coefficients (based on crystal size) are 0.449 and 0.753.[2] Additional crystal and refinement information can be found in the tables.

**Structure solution and refinement**

The space group *C*2/*c* (15) was determined based on intensity statistics and systematic absences. The structure was solved by SHELXT 2018/2 and refined with full-matrix least squares / difference Fourier cycles using SHELXL-2019/1; $Z$ = 16 for the formula unit $C_{93}H_{142}N_6P_6$.[3,4] Non-hydrogen atoms were refined with anisotropic displacement parameters. The hydrogen atoms were placed in ideal positions and refined as riding atoms with relative isotropic displacement parameters. Remaining electron density indicated that additional partial solvent (hexane) was present in the structure. However, solvent models with strong sets of restraints and constraints did not converge to a chemically sensible structure. Therefore, the structure was investigated for solvent accessible areas.[5] Four voids were found in the unit cell (~8050 Å$^3$) to contain 1662 electrons. For comparison, hexane occupies ca. 163 Å$^3$ with 50 electrons.[6] Based on these values, we estimate that there are two molecules of hexane per formula unit. The contribution of the unidentified solvent to the structure factors was assessed by back-Fourier transformation[5] and the data were corrected accordingly. The refinement using the modified dataset improved the overall structure and *R* values. The final anisotropic full-matrix least-squares refinement on $F^2$ with 1675 variables against 34464 data points and converged at $R_1$ = 5.53%, for the observed data and w$R_2$ = 14.64% for all data. The goodness-of-fit on $F^2$ was 1.020. The largest peak in the final difference electron density synthesis was 0.753 $e^-$/Å$^3$ and the deepest hole was −0.36 $e^-$/Å$^3$ with an RMS deviation of 0.05 $e^-$/Å$^3$. On the basis of the final model, the calculated density was 1.082 g/cm$^3$ and *F*(000), 13312 $e^-$.

**Table S3.** Crystal data and structure refinement for **2** (CCDC 2477494).

| | | |
|---|---|---|
| Empirical formula | $C_{54}H_{76}N_4P_4$ | |
| Formula weight | 905.06 | |
| Crystal color, shape, size | red block, 0.296 × 0.490 × 0.579 $mm^3$ | |
| Temperature | 173(2) K | |
| Wavelength | 0.71073 Å (Mo Kα) | |
| Crystal system, space group | Triclinic, *P*–1 | |
| Unit cell dimensions | *a* = 13.5678(6) Å | α = 79.661(2)° |
| | *b* = 13.7534(7) Å | β = 86.029(2)° |
| | *c* = 15.5834(7) Å | γ = 65.260(2)° |
| Volume | 2598.1(2) $Å^3$ | |
| *Z* | 2 | |
| Density (calculated) | 1.157 $mg/m^3$ | |
| Absorption coefficient | 0.184 $mm^{-1}$ | |
| *F*(000) | 976 | |
| ***Data collection*** | | |
| Diffractometer | Venture D8, Bruker | |
| Source | Iμ3.0, Incoatec | |
| Detector | Photon III | |
| Theta range for data collection | 2.066 to 28.312° | |
| Index ranges | –18 ≤ h ≤ 18, –18 ≤ k ≤ 18, –20 ≤ l ≤ 20 | |
| Reflections collected | 150181 | |
| Independent reflections | 12922 [$R_{int}$ = 0.0914] | |
| Observed Reflections [I > 2σ(I)] | 11188 | |
| Completeness to theta = 25.242° | 99.9% | |
| ***Solution and Refinement*** | | |
| Absorption correction | Multi-scan | |
| Max. and min. transmission | 0.948 and 0.901 | |
| Solution | Intrinsic methods | |
| Refinement method | Full-matrix least-squares on $F^2$ | |
| Weighting scheme | $w = [\sigma^2F_o^2 + AP^2 + BP]^{-1}$, with | |
| | $P = (F_o^2 + 2 F_c^2)/3$, A = 0.0709, B = 0.90 | |
| Data / restraints / parameters | 12922 / 0 / 563 | |
| Goodness-of-fit on $F^2$ | 1.104 | |
| Final *R* indices [*I*>2σ(I)] | $R_1$ = 0.0518, $wR_2$ = 0.1260 | |
| *R* indices (all data) | $R_1$ = 0.0597, $wR_2$ = 0.1364 | |
| Largest diff. peak and hole | 0.89 and –0.37 e·$Å^{-3}$ | |

Goodness-of-fit = $[\Sigma[w(F_o^2 - F_c^2)^2]/N_{observns} - N_{params})]^{1/2}$, all data. $R_1 = \Sigma(|F_o| - |F_c|) / \Sigma |F_o|$. $wR_2 = [\Sigma[w(F_o^2 - F_c^2)^2] / \Sigma [w(F_o^2)^2]]^{1/2}$.

**Crystal structure data for 2**

A red, block-shaped specimen of **2** ($C_{54}H_{76}N_4P_4$; IUMSC 24138), approximate dimensions 0.296 × 0.490 × 0.579 $mm^3$, was placed on a Kapton mount with inert oil for crystal structure determination. The X-ray intensity data were measured at 173(2) K on a Bruker D8 Venture Kappa diffractometer equipped with a microfocus sealed tube (λ = 0.71073 Å) and a multilayer mirror monochromator.

**Data collection**

The data collection was carried out at 173 K using Cu Kα radiation using 1° ω and φ scans with a frame time of 1 and 2 seconds and a detector distance of 4.00 cm. Overall, 2005 frames were collected with a total exposure time of 0.98 hours. The frames were integrated with the SAINT V8.40B package using a narrow-frame algorithm.[1] The integration of the data using a triclinic unit cell yielded 150181 reflections to a maximum θ angle of 28.31° (0.75 Å resolution), of which 12922 were independent (average redundancy 11.62, completeness = 99.9%, $R_{int}$ = 9.14%, $R_{sig}$ = 4.76%) and 11188 (86.6%) were greater than $2\sigma(F^2)$. The final cell constants of $a$ = 13.5678(6) Å, $b$ = 13.7534(7) Å, $c$ = 15.5834(7) Å, α = 79.661(2)°, β = 86.029(2)°, γ = 65.260(2)°, volume = 2598.1(2) $Å^3$, are based upon the refinement of the XYZ-centroids of 9985 reflections above 20 σ(I) with 2.38° < 2θ < 28.22°. Data were corrected for absorption effects using the Multi-Scan method in SADABS 2016/2. The calculated minimum and maximum transmission coefficients (based on crystal size) are 0.901 and 0.948.[2] Additional crystal and refinement information can be found in the tables.

## Structure solution and refinement

The space group $P$–1 (2) was determined based on intensity statistics and systematic absences. The structure was solved by SHELXT 2018/2 and refined with full-matrix least squares / difference Fourier cycles using SHELXL-2019/1; $Z = 2$ for the formula unit $C_{54}H_{76}N_4P_4$.[3,4] Non-hydrogen atoms were refined with anisotropic displacement parameters. The hydrogen atoms were placed in ideal positions and refined as riding atoms with relative isotropic displacement parameters. The final anisotropic full-matrix least-squares refinement on $F^2$ with 563 variables against 12922 data points and converged at $R_1$ = 5.18%, for the observed data and w$R_2$ = 13.64% for all data. The goodness-of-fit on $F^2$ was 1.10. The largest peak in the final difference electron density synthesis was 0.89 $e^-/Å^3$ and the deepest hole was −0.37 $e^-/Å^3$ with an RMS deviation of 0.105 $e^-/Å^3$. On the basis of the final model, the calculated density was 1.16 g/cm$^3$ and $F$(000), 976 $e^-$.

**Table S4.** Crystal data and structure refinement for **3**·hex (CCDC 2477495).

| | | |
|---|---|---|
| Empirical formula | $C_{87}H_{128}N_6P_6$ | |
| Formula weight | 1443.77 | |
| Crystal color, shape, size | red block, 0.150 × 0.230 × 0.270 mm$^3$ | |
| Temperature | 173(2) K | |
| Wavelength | 1.54178 Å (Cu Kα) | |
| Crystal system, space group | Monoclinic, $P2_1/c$ | |
| Unit cell dimensions | $a$ = 12.8201(5) Å | α = 90° |
| | $b$ = 21.8399(8) Å | β = 92.626(2)° |
| | $c$ = 30.2910(10) Å | γ = 90° |
| Volume | 8472.3(5) Å$^3$ | |
| $Z$ | 4 | |
| Density (calculated) | 1.132 mg/m$^3$ | |
| Absorption coefficient | 1.521 mm$^{-1}$ | |
| $F$(000) | 3128 | |
| ***Data collection*** | | |
| Diffractometer | Venture D8, Bruker | |
| Source | Iμ3.0, Incoatec | |
| Detector | Photon III | |
| Theta range for data collection | 2.495 to 68.41° | |
| Index ranges | $-15 \le h \le 15$, $0 \le k \le 26$, $0 \le l \le 36$ | |
| Reflections collected | 487346 | |
| Independent reflections | 15559 [$R_{int}$ = 0.1876] | |
| Observed Reflections [I > 2σ(I)] | 12878 | |
| Completeness to theta = 67.679° | 100% | |
| ***Solution and Refinement*** | | |
| Absorption correction | Multi-scan | |
| Max. and min. transmission | 0.753 and 0.386 | |
| Solution | Intrinsic methods | |
| Refinement method | Full-matrix least-squares on $F^2$ | |
| Weighting scheme | $w = [\sigma^2 F_o^2 + AP^2 + BP]^{-1}$, with | |
| | $P = (F_o^2 + 2 F_c^2)/3$, A = 0.1198, B = 4.28 | |
| Data / restraints / parameters | 241826 / 129 / 947 | |
| Goodness-of-fit on $F^2$ | 1.094 | |
| Final $R$ indices [$I$>2σ(I)] | $R_1$ = 0.0763, $wR_2$ = 0.2004 | |
| $R$ indices (all data) | $R_1$ = 0.0893, $wR_2$ = 0.2102 | |
| Largest diff. peak and hole | 0.52 and –0.48 e·Å$^{-3}$ | |

Goodness-of-fit = $[\Sigma[w(F_o^2 - F_c^2)^2]/N_{observns} - N_{params})]^{1/2}$, all data. $R_1 = \Sigma(|F_o| - |F_c|) / \Sigma |F_o|$. $wR_2 = [\Sigma[w(F_o^2 - F_c^2)^2] / \Sigma [w(F_o^2)^2]]^{1/2}$.

**Crystal structure data for 3·hex**

A red, block-shaped specimen of **3**·hex ($C_{87}H_{128}N_6P_6$; IUMSC 24299), approximate dimensions 0.150 × 0.230 × 0.270 $mm^3$, was placed on a Kapton mount with inert oil for crystal structure determination. The X-ray intensity data were measured on a Bruker D8 Venture Kappa diffractometer equipped with a microfocus sealed tube (λ = 1.54178 Å) and a multilayer mirror monochromator.

**Data collection**

The data collection was performed at 173 K using 1° ω and φ scans, frame times of 1 and 10 seconds, and a detector distance of 5.0 cm. Overall, 2873 frames were collected with a total exposure time of 4.93 hours. The frames were integrated with the SAINT V8.40B package using a narrow-frame algorithm.[1] The integration of the data using a monoclinic unit cell yielded 487346 reflections to a maximum θ angle of 68.41° (0.83 Å resolution), of which 15559 were independent (average redundancy 15.54, completeness = 100.0%, $R_{int}$ = 18.76%, $R_{sig}$ = 7.81%) and 12878 (82.8%) were greater than 2σ($F^2$). The final cell constants of a = 12.8201(5) Å, b = 21.8399(8) Å, c = 30.2910(10) Å, α = 90°, β = 92.626(2)°, γ = 90°, volume = 8472.3(5) $Å^3$, are based upon the refinement of the XYZ-centroids of 9884 reflections above 20 σ(*I*) with 2.49° < 2θ < 68.31°. Data were corrected for absorption effects using the Multi-Scan method in TWINABS Bruker. The calculated minimum and maximum transmission coefficients (based on crystal size) are 0.386 and 0.753.[2] Additional crystal and refinement information can be found in the tables.

**Structure solution and refinement**

The space group $P2_1/c$ (14) was determined based on intensity statistics and systematic absences. The structure was solved by XT, VERSION 2018/2 and refined with full-matrix least squares / difference Fourier cycles using SHELXL-2019/1; $Z$ = 4 for the formula unit $C_{87}H_{128}N_6P_6$.[3,4] Non-hydrogen atoms were refined with anisotropic displacement parameters. The hydrogen atoms were placed in ideal positions and refined as riding atoms with relative isotropic displacement parameters. The final anisotropic full-matrix least-squares refinement on $F^2$ with 947 variables against 241826 data points and 129 restraints converged at $R_1$ = 7.63%, for the observed data and $wR_2$ = 21.02% for all data. The goodness-of-fit on $F^2$ was 1.09. The largest peak in the final difference electron density synthesis was 0.52 $e^-/Å^3$ and the deepest hole was −0.48 $e^-/Å^3$ with an RMS deviation of 0.078 $e^-/Å^3$. On the basis of the final model, the calculated density was 1.14 $g/cm^3$ and $F(000)$, 3128 $e^-$.

**Table S5.** Crystal data and structure refinement for **4** (CCDC 2477496).

| | | |
|---|---|---|
| Empirical formula | $C_{81}H_{114}N_6P_6S_3$ | |
| Formula weight | 1453.78 | |
| Crystal color, shape, size | red block, 0.142 × 0.253 × 0.343 mm$^3$ | |
| Temperature | 173(2) K | |
| Wavelength | 1.54178 Å (Cu Kα) | |
| Crystal system, space group | Orthorhombic, *Pbca* | |
| Unit cell dimensions | *a* = 24.4687(9) Å | α = 90° |
| | *b* = 16.2353(6) Å | β = 90° |
| | *c* = 41.6041(16) Å | γ = 90° |
| Volume | 16527.5(11) Å$^3$ | |
| *Z* | 8 | |
| Density (calculated) | 1.169 mg/m$^3$ | |
| Absorption coefficient | 2.255 mm$^{-1}$ | |
| *F*(000) | 6240 | |
| ***Data collection*** | | |
| Diffractometer | Venture D8, Bruker | |
| Source | Iμ3.0, Incoatec | |
| Detector | Photon III | |
| Theta range for data collection | 2.125 to 68.79° | |
| Index ranges | –29 ≤ h ≤ 29, –19 ≤ k ≤ 19, –50 ≤ l ≤ 50 | |
| Reflections collected | 322526 | |
| Independent reflections | 15196 [$R_{int}$ = 0.1452] | |
| Observed Reflections [I > 2σ(I)] | 13520 | |
| Completeness to theta = 67.679° | 100% | |
| ***Solution and Refinement*** | | |
| Absorption correction | Multi-scan | |
| Max. and min. transmission | 0.753 and 0.490 | |
| Solution | Intrinsic methods | |
| Refinement method | Full-matrix least-squares on $F^2$ | |
| Weighting scheme | $w = [\sigma^2F_o^2 + AP^2 + BP]^{-1}$, with | |
| | $P = (F_o^2 + 2 F_c^2)/3$, A = 0.0771, B = 17.91 | |
| Data / restraints / parameters | 15196 / 0 / 865 | |
| Goodness-of-fit on $F^2$ | 1.090 | |
| Final *R* indices [I>2σ(I)] | $R_1$ = 0.0604, $wR_2$ = 0.1540 | |
| *R* indices (all data) | $R_1$ = 0.0672, $wR_2$ = 0.1610 | |
| Largest diff. peak and hole | 1.30 and –0.46 e·Å$^{-3}$ | |

Goodness-of-fit = $[\Sigma[w(F_o^2 - F_c^2)^2]/N_{observns} - N_{params})]^{1/2}$, all data. $R_1 = \Sigma(|F_o| - |F_c|) / \Sigma |F_o|$. $wR_2 = [\Sigma[w(F_o^2 - F_c^2)^2] / \Sigma [w(F_o^2)^2]]^{1/2}$.

## Crystal structure data for 4

A red, block-shaped specimen of **4** ($C_{81}H_{114}N_6P_6S_3$; IUMSC 24347), approximate dimensions 0.142 × 0.253 × 0.343 $mm^3$, was placed on a Kapton mount with inert oil for crystal structure determination. The X-ray intensity data were measured on a Bruker D8 Venture Kappa diffractometer equipped with a microfocus sealed tube (λ = 1.54178 Å) and a multilayer mirror monochromator.

## Data collection

The data collection was performed at 173 K using 1° ω and φ scans, frame times of 0.70, 1 and 30 seconds, and a detector distance of 4.0 cm. Overall, 3168 frames were collected with a total exposure time of 15.98 hours. The frames were integrated with the SAINT V8.40B package using a narrow-frame algorithm.[1] The integration of the data using an orthorhombic unit cell yielded 322526 reflections to a maximum θ angle of 68.79° (0.83 Å resolution), of which 15196 were independent (average redundancy 21.22, completeness = 100.0%, $R_{int}$ = 14.52%, $R_{sig}$ = 4.64%) and 13520 (89.0%) were greater than $2\sigma(F^2)$. The final cell constants of $a$ = 24.4687(9) Å, $b$ = 16.2353(6) Å, $c$ = 41.6041(16) Å, α = 90°, β = 90°, γ = 90°, volume = 16527.5(11) $Å^3$, are based upon the refinement of the XYZ-centroids of 9788 reflections above 20 σ(I) with 4.19° < 2θ < 68.27°. Data were corrected for absorption effects using the Multi-Scan method in SADABS 2016/2. The calculated minimum and maximum transmission coefficients (based on crystal size) are 0.490 and 0.753.[2] Additional crystal and refinement information can be found in the tables.

**Structure solution and refinement**

The space group P*bca* (61) was determined based on intensity statistics and systematic absences. The structure was solved by SHELXT 2018/2 and refined with full-matrix least squares / difference Fourier cycles using SHELXL-2019/2; $Z$ = 8 for the formula unit $C_{81}H_{114}N_6P_6S_3$.[3,4] Non-hydrogen atoms were refined with anisotropic displacement parameters. The hydrogen atoms were placed in ideal positions and refined as riding atoms with relative isotropic displacement parameters. The final anisotropic full-matrix least-squares refinement on $F^2$ with 865 variables against 15196 data points and converged at $R_1$ = 6.04%, for the observed data and $wR_2$ = 16.10% for all data. The goodness-of-fit on $F^2$ was 1.09. The largest peak in the final difference electron density synthesis was 1.03 $e^-/Å^3$ and the deepest hole was −0.46 $e^-/Å^3$ with an RMS deviation of 0.090 $e^-/Å^3$. On the basis of the final model, the calculated density was 1.17 $g/cm^3$ and $F(000)$, 6240 $e^-$.

**Table S6.** Crystal data and structure refinement for **5**·3hex (CCDC 2477497).

| | | |
|---|---|---|
| Empirical formula | $C_{99}H_{156}N_6P_6Se_3$ | |
| Formula weight | 1852.99 | |
| Crystal color, shape, size | reddish-brown needle, 0.111 × 0.116 × 0.206 $mm^3$ | |
| Temperature | 143(2) K | |
| Wavelength | 1.54178 Å (Cu Kα) | |
| Crystal system, space group | Orthorhombic, *Pbca* | |
| Unit cell dimensions | *a* = 30.3164(11) Å | α = 90° |
| | *b* = 13.0284(5) Å | β = 90° |
| | *c* = 49.9982(17) Å | γ = 90° |
| Volume | 19748.0(12) $Å^3$ | |
| *Z* | 8 | |
| Density (calculated) | 1.246 $mg/m^3$ | |
| Absorption coefficient | 2.626 $mm^{-1}$ | |
| F(000) | 7872 | |
| ***Data collection*** | | |
| Diffractometer | Venture D8, Bruker | |
| Source | Iμ3.0, Incoatec | |
| Detector | Photon III | |
| Theta range for data collection | 2.29 to 68.465° | |
| Index ranges | –33 ≤ h ≤ 36, –15 ≤ k ≤ 15, –60 ≤ l ≤ 60 | |
| Reflections collected | 190972 | |
| Independent reflections | 18028 [$R_{int}$ = 0.1579] | |
| Observed Reflections [I > 2σ(I)] | 13569 | |
| Completeness to theta = 67.679° | 99.5% | |
| ***Solution and Refinement*** | | |
| Absorption correction | Multi-scan | |
| Max. and min. transmission | 0.753 and 0.490 | |
| Solution | Intrinsic methods | |
| Refinement method | Full-matrix least-squares on $F^2$ | |
| Weighting scheme | $w = [\sigma^2F_o^2 + AP^2 + BP]^{-1}$, with | |
| | $P = (F_o^2 + 2 F_c^2)/3$, A = 0.0147, B = 153.16 | |
| Data / restraints / parameters | 18028 / 762 / 865 | |
| Goodness-of-fit on $F^2$ | 1.091 | |
| Final *R* indices [I>2σ(I)] | $R_1$ = 0.0934, $wR_2$ = 0.1863 | |
| *R* indices (all data) | $R_1$ = 0.1180, $wR_2$ = 0.1964 | |
| Largest diff. peak and hole | 1.15 and –1.99 e·$Å^{-3}$ | |

Goodness-of-fit = $[\Sigma[w(F_o^2 - F_c^2)^2]/N_{observns} - N_{params})]^{1/2}$, all data. R1 = $\Sigma(|F_o| - |F_c|) / \Sigma |F_o|$. wR2 = $[\Sigma[w(F_o^2 - F_c^2)^2] / \Sigma [w(F_o^2)^2]]^{1/2}$.

**Crystal structure data for 5·3hex**

A reddish-brown, needle-shaped specimen of **5**·3hex ($C_{99}H_{156}N_6P_6Se_3$; IUMSC 24352), approximate dimensions 0.111 × 0.116 × 0.260 $mm^3$, was placed on a Kapton mount with inert oil for crystal structure determination. The X-ray intensity data were measured on a Bruker D8 Venture Kappa diffractometer equipped with a microfocus sealed tube (λ = 1.54178 Å) and a multilayer mirror monochromator.

**Data collection**

The data collection was performed at 173 K using 1° ω and φ scans, frame times of 1, 9, 10, 25 and 70 seconds, and a detector distance of 4.3 cm. Overall, 2188 frames were collected with a total exposure time of 22.29 hours. The frames were integrated with the SAINT V8.41 package using a narrow-frame algorithm.[1] The integration of the data using an orthorhombic unit cell yielded 190972 reflections to a maximum θ angle of 68.47° (0.83 Å resolution), of which 18028 were independent (average redundancy 10.59, completeness = 99.5%, $R_{int}$ = 15.79%, $R_{sig}$ = 6.96%) and 13569 (75.3%) were greater than $2\sigma(F^2)$. The final cell constants of a = 30.3164(11) Å, b = 13.0284(5) Å, c = 49.9982(17) Å, α = 90°, β = 90°, γ = 90°, volume = 19748.0(12) $Å^3$, are based upon the refinement of the XYZ-centroids of 9885 reflections above 20 $\sigma(I)$ with 2.29° < 2θ < 67.48°. Data were corrected for absorption effects using the Multi-Scan method in SADABS 2016/2. The calculated minimum and maximum transmission coefficients (based on crystal size) are 0.548 and 0.759.[2] Additional crystal and refinement information can be found in the tables.

**Structure solution and refinement**

The space group *Pbca* (61) was determined based on intensity statistics and systematic absences. The structure was solved by SHELXT 2018/2 and refined with full-matrix least squares / difference Fourier cycles using SHELXL-2019/2; $Z$ = 8 for the formula unit $C_{99}H_{156}N_6P_6Se_3$.[3,4] Non-hydrogen atoms were refined with anisotropic displacement parameters. The hydrogen atoms were placed in ideal positions and refined as riding atoms with relative isotropic displacement parameters. Remaining electron density indicated that additional partial solvent (hexane) was present in the structure. However, solvent models with strong sets of restraints and constraints did not converge to a chemically sensible structure. Therefore, the structure was investigated for solvent accessible areas.[5] Four voids were found in the unit cell (~4420 $Å^3$) to contain 1052 electrons. For comparison, hexane occupies ca. 163 $Å^3$ with 50 electrons.[6] Based on these values, we estimate that there are three molecules of hexane per formula unit. The contribution of the unidentified solvent to the structure factors was assessed by back-Fourier transformation[5] and the data were corrected accordingly. The refinement using the modified dataset improved the overall structure and R values. The final anisotropic full-matrix least-squares refinement on $F^2$ with 865 variables against 18194 data points and 762 restraints converged at $R_1$ = 11.80%, for the observed data and $wR_2$ = 19.64% for all data. The goodness-of-fit on $F^2$ was 1.093. The largest peak in the final difference electron density synthesis was 1.15 $e^-/Å^3$ and the deepest hole was −1.99 $e^-/Å^3$ with an RMS deviation of 0.098 $e^-/Å^3$. On the basis of the final model, the calculated density was 1.25 $g/cm^3$ and $F(000)$, 7872 $e^-$.

## 4. Computational details

### 4.1 General

Geometry optimizations of the molecules used in the main manuscript were performed without symmetry constraints using the Orca 6.0.1 program[7] at the ωB97x-D3BJ[8]/def2-SVP[9] level using the Resolution of Identity approximation (RIJCOSX).[10] Reactants and products were characterized by frequency calculations and have positive definite Hessian matrices whereas transition states show only one negative eigenvalue in their diagonalized force constant matrices. Energy refinements were carried out by means of single-point calculations at the M062X[11]/def2-TZVPP[9] level on the ωB97x-D3BJ/def2-SVP geometries. Solvent effects (solvent = hexane) were also considered during both the geometry optimizations and the single-point calculations by means of the conductor-like polarizable continuum (CPCM).[12] This level is denoted CPCM(hexane)-M062X/def2-TZVPP//CPCM(hexane)-ωB97x-D3BJ/def2-SVP. The computed thermochemistry data were computed at 298.15 K and corrected to a standard concentration of 1 M.

### 4.2 Nuclear magnetic resonance DFT simulations

Chemical shifts and $^{31}P$–$^{31}P$ coupling constants were obtained with the Orca 6.0.1 program at the (gas-phase)-ωB97x-D3BJ/def2-TZVPP//(gas-phase)-ωB97x-D3BJ/def2-SVP level of theory. As mentioned in the main manuscript, a benchmark study of these values for **1** was performed with several functionals to compare with the experimental results. These results are given as an average of the corresponding *quasi*-equivalent phosphorus atoms, and the chemical shifts are referenced to phosphoric acid. Data is presented in Table S6 and indicates that most functionals correctly describe the chemical shift of the outer phosphanyl phosphorus, while there

are larger differences when assessing the phosphabenzene phosphorus shifts, with BP86-D3BJ giving the best overall results.

Regarding the coupling constants, the computed through-space $J_{1,6}$ is underestimated in all cases, while both the through-bond $J_{1,2}$ and $J_{1,4}$ appear to be overestimated. After comparison of all functionals, it is found that the values obtained at the ωB97x-D3BJ level concur reasonably well with the experimental coupling constants.

**Table S7.** Density functional benchmark of the computed chemical shifts and coupling constants. Computed chemical shifts were referenced to phosphoric acid. Geometries were obtained at the ωB97X-D3BJ/def2-SVP level of theory, while the NMR calculations were obtained with a single point calculation at the DFT/def2-TZVPP level of theory.

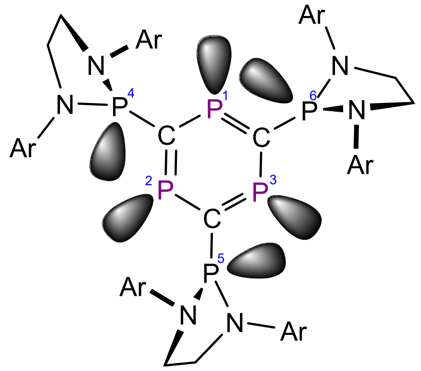


| | $P_1$ | $P_6$ | $J_{1,6}$ | $J_{1,2}$ | $J_{1,4}$ |
|---|---|---|---|---|---|
| Experimental | 295.5 | 118.5 | 432.0 | ~0 | ~0 |
| ωB97X-D3BJ | 364.5 | 116.5 | 346.5 | 12.4 | 13.2 |
| BP86-D3BJ | 304.5 | 119.6 | 357.8 | 40.7 | 23.0 |
| B3LYP-D3BJ | 339.4 | 126.4 | 348.1 | 25.0 | 19.2 |
| PBE-D3BJ | 301.9 | 115.4 | 356.1 | 43.3 | 23.5 |
| PBE0-D3BJ | 327.6 | 114.7 | 337.6 | 20.6 | 18.0 |
| TPSS | 264.5 | 91.3 | 324.1 | 27.1 | 22.0 |
| PW91 | 305.0 | 118.1 | 357.0 | 42.1 | 23.3 |
| M06-2X | 450.5 | 156.3 | 347.1 | 49.0 | 22.9 |

### 4.3 Cartesian coordinates

Cartesian coordinates (in Å) and total energies (in hartrees) of all the stationary points discussed in the text.

**ORCA (Single points CPCM(hexane)-M062X/def2-TZVPP // Geometries CPCM(hexane)-ωB97x-D3BJ/def2-SVP**
**INT0**

$N_{imag}$ = 0
$SP_{M062X}$ = -1529.398960
$G_{M062X}$ = -1529.071840

| | | | |
|---|---|---|---|
| P | 0.189344000000 | 3.315511000000 | -1.511946000000 |
| P | -0.013854000000 | 0.055873000000 | -0.726893000000 |
| N | -1.240056000000 | -0.012612000000 | 0.458157000000 |
| N | 1.184880000000 | -0.121011000000 | 0.489121000000 |
| C | 0.083957000000 | 1.843540000000 | -1.056600000000 |
| C | -0.752404000000 | 0.456427000000 | 1.752115000000 |
| H | -1.400321000000 | 0.079449000000 | 2.557283000000 |
| H | -0.731838000000 | 1.560514000000 | 1.807634000000 |
| C | 0.672280000000 | -0.095726000000 | 1.857952000000 |
| H | 0.667776000000 | -1.117532000000 | 2.282813000000 |
| H | 1.291356000000 | 0.539900000000 | 2.511056000000 |
| C | -2.604259000000 | -0.320482000000 | 0.196570000000 |
| C | -3.597631000000 | 0.670194000000 | 0.323988000000 |
| C | -4.931419000000 | 0.321541000000 | 0.080695000000 |
| H | -5.706236000000 | 1.088356000000 | 0.176751000000 |
| C | -5.276828000000 | -0.973663000000 | -0.298646000000 |
| H | -6.322546000000 | -1.228209000000 | -0.493797000000 |
| C | -4.284530000000 | -1.943937000000 | -0.428536000000 |
| H | -4.551670000000 | -2.964853000000 | -0.717883000000 |
| C | -2.943434000000 | -1.638034000000 | -0.174918000000 |
| C | 2.554461000000 | -0.393235000000 | 0.216946000000 |
| C | 3.525850000000 | 0.592030000000 | 0.486601000000 |
| C | 4.869584000000 | 0.304882000000 | 0.217785000000 |
| H | 5.627617000000 | 1.067819000000 | 0.420561000000 |
| C | 5.246449000000 | -0.926195000000 | -0.314030000000 |
| H | 6.299662000000 | -1.133420000000 | -0.524799000000 |
| C | 4.278051000000 | -1.894548000000 | -0.575206000000 |
| H | 4.571756000000 | -2.866194000000 | -0.983968000000 |
| C | 2.927483000000 | -1.647982000000 | -0.307943000000 |
| C | 1.889578000000 | -2.709950000000 | -0.570298000000 |
| H | 1.197835000000 | -2.804243000000 | 0.279706000000 |
| H | 1.280418000000 | -2.462453000000 | -1.454572000000 |
| H | 2.365600000000 | -3.685120000000 | -0.747335000000 |
| C | 3.139839000000 | 1.931364000000 | 1.064612000000 |
| H | 3.131387000000 | 1.897525000000 | 2.167454000000 |
| H | 3.861788000000 | 2.705852000000 | 0.766562000000 |
| H | 2.138419000000 | 2.233584000000 | 0.730498000000 |
| C | -3.241916000000 | 2.086257000000 | 0.705792000000 |
| H | -2.322138000000 | 2.411600000000 | 0.199993000000 |

| | | | |
|---|---|---|---|
| H | -4.055010000000 | 2.775185000000 | 0.435013000000 |
| H | -3.074463000000 | 2.180405000000 | 1.791460000000 |
| C | -1.882106000000 | -2.702961000000 | -0.282656000000 |
| H | -1.242716000000 | -2.543528000000 | -1.165643000000 |
| H | -1.221778000000 | -2.686176000000 | 0.597477000000 |
| H | -2.336681000000 | -3.700431000000 | -0.367654000000 |

**TS1**

$\boldsymbol{N_{imag}}$ = 0, -137.9 $cm^{-1}$
$\boldsymbol{SP_{M062X}}$ = -3058.799640
$\boldsymbol{G_{M062X}}$ = -3058.121000

| | | | |
|---|---|---|---|
| P | 0.285889000000 | -0.785580000000 | -2.044968000000 |
| P | -0.084753000000 | 1.362336000000 | 0.983306000000 |
| P | 2.286653000000 | 0.594213000000 | -0.136941000000 |
| P | -2.519654000000 | -0.368474000000 | -0.469830000000 |
| N | 3.150556000000 | 1.970424000000 | -0.616650000000 |
| N | 3.687491000000 | -0.137412000000 | 0.475609000000 |
| N | -3.741632000000 | -0.156293000000 | 0.725091000000 |
| N | -2.358018000000 | -2.016796000000 | 0.029398000000 |
| C | 1.665553000000 | -0.277736000000 | -1.525024000000 |
| C | -1.059308000000 | 0.394370000000 | 0.223857000000 |
| C | 4.581663000000 | 1.909068000000 | -0.323182000000 |
| H | 4.819653000000 | 2.472362000000 | 0.597479000000 |
| H | 5.167443000000 | 2.339943000000 | -1.151571000000 |
| C | 4.890534000000 | 0.421446000000 | -0.144300000000 |
| H | 5.100499000000 | -0.039343000000 | -1.125761000000 |
| H | 5.758418000000 | 0.259675000000 | 0.512490000000 |
| C | 2.489361000000 | 3.221134000000 | -0.818403000000 |
| C | 2.179562000000 | 3.603618000000 | -2.137328000000 |
| C | 1.506316000000 | 4.810876000000 | -2.344558000000 |
| H | 1.256870000000 | 5.116294000000 | -3.365027000000 |
| C | 1.148846000000 | 5.620027000000 | -1.265737000000 |
| H | 0.614184000000 | 6.558208000000 | -1.440190000000 |
| C | 1.470381000000 | 5.234361000000 | 0.033044000000 |
| H | 1.187524000000 | 5.869209000000 | 0.878155000000 |
| C | 2.149494000000 | 4.034176000000 | 0.278523000000 |
| C | 3.678470000000 | -1.383819000000 | 1.168934000000 |
| C | 4.000703000000 | -2.584114000000 | 0.507733000000 |
| C | 3.999529000000 | -3.774289000000 | 1.246279000000 |
| H | 4.248095000000 | -4.712199000000 | 0.740425000000 |
| C | 3.664260000000 | -3.780239000000 | 2.598308000000 |
| H | 3.659119000000 | -4.720265000000 | 3.157296000000 |
| C | 3.322003000000 | -2.587573000000 | 3.233817000000 |
| H | 3.050142000000 | -2.589538000000 | 4.293447000000 |
| C | 3.327937000000 | -1.377178000000 | 2.533684000000 |
| C | -3.967881000000 | -1.300370000000 | 1.599861000000 |
| H | -4.881548000000 | -1.848940000000 | 1.298202000000 |
| H | -4.091371000000 | -0.989460000000 | 2.650733000000 |
| C | -2.726136000000 | -2.174450000000 | 1.433079000000 |
| H | -1.936879000000 | -1.821830000000 | 2.121190000000 |
| H | -2.932815000000 | -3.233072000000 | 1.652261000000 |
| C | -4.645112000000 | 0.940497000000 | 0.714781000000 |
| C | -5.607347000000 | 1.053209000000 | -0.309767000000 |
| C | -6.479747000000 | 2.147072000000 | -0.299260000000 |
| H | -7.227444000000 | 2.240771000000 | -1.092886000000 |

| | | | |
|---|---|---|---|
| C | -6.412941000000 | 3.104681000000 | 0.711877000000 |
| H | -7.102240000000 | 3.954195000000 | 0.708028000000 |
| C | -5.467889000000 | 2.976655000000 | 1.727981000000 |
| H | -5.411877000000 | 3.729055000000 | 2.520930000000 |
| C | -4.572760000000 | 1.900752000000 | 1.743891000000 |
| C | -1.548561000000 | -2.940461000000 | -0.680878000000 |
| C | -2.020793000000 | -3.405774000000 | -1.927756000000 |
| C | -1.213338000000 | -4.259842000000 | -2.684116000000 |
| H | -1.575274000000 | -4.612786000000 | -3.654586000000 |
| C | 0.034103000000 | -4.668678000000 | -2.214490000000 |
| H | 0.657174000000 | -5.335530000000 | -2.817368000000 |
| C | 0.484105000000 | -4.219926000000 | -0.975459000000 |
| H | 1.469549000000 | -4.520146000000 | -0.606263000000 |
| C | -0.287027000000 | -3.347987000000 | -0.196621000000 |
| C | -3.367021000000 | -2.969948000000 | -2.445236000000 |
| H | -3.339308000000 | -1.920823000000 | -2.782727000000 |
| H | -3.681224000000 | -3.594220000000 | -3.294230000000 |
| H | -4.127347000000 | -3.026853000000 | -1.652209000000 |
| C | 0.278850000000 | -2.819650000000 | 1.096804000000 |
| H | 1.351560000000 | -3.038345000000 | 1.159646000000 |
| H | 0.142838000000 | -1.730898000000 | 1.164386000000 |
| H | -0.206941000000 | -3.275112000000 | 1.974330000000 |
| C | 4.321030000000 | -2.611699000000 | -0.966561000000 |
| H | 4.177667000000 | -3.624336000000 | -1.370129000000 |
| H | 3.671835000000 | -1.922213000000 | -1.525158000000 |
| H | 5.368192000000 | -2.323608000000 | -1.155838000000 |
| C | 2.957888000000 | -0.088084000000 | 3.222197000000 |
| H | 3.705451000000 | 0.694426000000 | 3.023312000000 |
| H | 1.988309000000 | 0.292408000000 | 2.859657000000 |
| H | 2.876024000000 | -0.234483000000 | 4.308555000000 |
| C | -3.554598000000 | 1.768972000000 | 2.849406000000 |
| H | -3.945133000000 | 1.148570000000 | 3.674242000000 |
| H | -2.637134000000 | 1.291979000000 | 2.478659000000 |
| H | -3.303042000000 | 2.754025000000 | 3.269495000000 |
| C | -5.696889000000 | 0.012298000000 | -1.398208000000 |
| H | -6.654346000000 | 0.088867000000 | -1.933782000000 |
| H | -4.883466000000 | 0.134631000000 | -2.131405000000 |
| H | -5.602409000000 | -1.003218000000 | -0.985690000000 |
| C | 2.514946000000 | 3.653157000000 | 1.692359000000 |
| H | 3.495199000000 | 4.077025000000 | 1.968103000000 |
| H | 1.771861000000 | 4.041568000000 | 2.403568000000 |
| H | 2.578560000000 | 2.565214000000 | 1.824219000000 |
| C | 2.574953000000 | 2.721484000000 | -3.295177000000 |
| H | 2.069299000000 | 1.745907000000 | -3.240107000000 |
| H | 2.318550000000 | 3.196592000000 | -4.252993000000 |
| H | 3.657590000000 | 2.519637000000 | -3.284632000000 |

**TS1A**

$\boldsymbol{N_{imag}}$ **=** 0, 336,9 cm$^{-1}$
$\boldsymbol{SP_{M062X}}$ **=** -3058.774000
$\boldsymbol{G_{M062X}}$ **=** -3058.095570

| | | | |
|---|---|---|---|
| P | -1.394038000000 | -0.634244000000 | 2.682016000000 |
| P | 0.838025000000 | 0.607503000000 | 2.089174000000 |
| P | -1.441136000000 | 1.214162000000 | -0.128822000000 |

| | | | |
|---|---|---|---|
| P | 2.138061000000 | -2.002686000000 | 0.716374000000 |
| N | -1.166901000000 | 2.860128000000 | 0.257068000000 |
| N | -3.125768000000 | 1.516584000000 | -0.231979000000 |
| N | 3.307446000000 | -1.975764000000 | -0.537917000000 |
| N | 1.107433000000 | -3.001143000000 | -0.255886000000 |
| C | -1.280710000000 | 0.394500000000 | 1.495178000000 |
| C | 1.577316000000 | -0.482244000000 | 1.139720000000 |
| C | -2.345729000000 | 3.470954000000 | 0.868023000000 |
| H | -2.299095000000 | 4.564786000000 | 0.762456000000 |
| H | -2.430780000000 | 3.227575000000 | 1.941762000000 |
| C | -3.536268000000 | 2.873702000000 | 0.117186000000 |
| H | -3.765070000000 | 3.455607000000 | -0.795918000000 |
| H | -4.438424000000 | 2.869072000000 | 0.751337000000 |
| C | 0.110816000000 | 3.481566000000 | 0.200726000000 |
| C | 0.710759000000 | 4.014223000000 | 1.362078000000 |
| C | 1.981231000000 | 4.590639000000 | 1.259646000000 |
| H | 2.452203000000 | 4.991451000000 | 2.162325000000 |
| C | 2.660257000000 | 4.635518000000 | 0.044216000000 |
| H | 3.659594000000 | 5.075892000000 | -0.012768000000 |
| C | 2.053565000000 | 4.119236000000 | -1.097345000000 |
| H | 2.569674000000 | 4.168194000000 | -2.061115000000 |
| C | 0.775521000000 | 3.552240000000 | -1.042548000000 |
| C | -4.045165000000 | 0.613667000000 | -0.835899000000 |
| C | -4.965982000000 | -0.079878000000 | -0.027835000000 |
| C | -5.852558000000 | -0.977613000000 | -0.635051000000 |
| H | -6.564104000000 | -1.528059000000 | -0.011903000000 |
| C | -5.823302000000 | -1.189857000000 | -2.011336000000 |
| H | -6.512115000000 | -1.904676000000 | -2.470544000000 |
| C | -4.917726000000 | -0.484902000000 | -2.804814000000 |
| H | -4.904038000000 | -0.638565000000 | -3.887989000000 |
| C | -4.030512000000 | 0.433164000000 | -2.233905000000 |
| C | 3.209858000000 | -3.173281000000 | -1.353011000000 |
| H | 3.681395000000 | -4.055370000000 | -0.875586000000 |
| H | 3.701603000000 | -3.007089000000 | -2.323523000000 |
| C | 1.701401000000 | -3.399176000000 | -1.527749000000 |
| H | 1.345827000000 | -2.790845000000 | -2.374088000000 |
| H | 1.475499000000 | -4.456367000000 | -1.744319000000 |
| C | 4.380228000000 | -1.048297000000 | -0.587618000000 |
| C | 5.626144000000 | -1.361558000000 | -0.016068000000 |
| C | 6.649227000000 | -0.407945000000 | -0.071938000000 |
| H | 7.621761000000 | -0.639250000000 | 0.373323000000 |
| C | 6.433441000000 | 0.832091000000 | -0.671941000000 |
| H | 7.239048000000 | 1.571722000000 | -0.700961000000 |
| C | 5.191878000000 | 1.132309000000 | -1.232553000000 |
| H | 5.022477000000 | 2.105930000000 | -1.702331000000 |
| C | 4.151812000000 | 0.197567000000 | -1.203887000000 |
| C | -0.269983000000 | -3.107306000000 | 0.065070000000 |
| C | -0.637883000000 | -3.739762000000 | 1.272756000000 |
| C | -1.990111000000 | -3.782972000000 | 1.634757000000 |
| H | -2.270395000000 | -4.268299000000 | 2.574741000000 |
| C | -2.972744000000 | -3.237689000000 | 0.810913000000 |
| H | -4.026697000000 | -3.280290000000 | 1.100556000000 |
| C | -2.602596000000 | -2.630432000000 | -0.385898000000 |
| H | -3.365271000000 | -2.188476000000 | -1.031127000000 |
| C | -1.259502000000 | -2.540370000000 | -0.767653000000 |
| C | -5.010208000000 | 0.138437000000 | 1.463370000000 |
| H | -5.729495000000 | 0.934509000000 | 1.721062000000 |

| | | | |
|---|---|---|---|
| H | -5.330217000000 | -0.776600000000 | 1.983432000000 |
| H | -4.027183000000 | 0.439787000000 | 1.847074000000 |
| C | -3.084729000000 | 1.229461000000 | -3.098136000000 |
| H | -3.184972000000 | 2.307645000000 | -2.899946000000 |
| H | -2.034921000000 | 0.964501000000 | -2.900969000000 |
| H | -3.287578000000 | 1.052086000000 | -4.163956000000 |
| C | 0.129306000000 | 3.041725000000 | -2.305040000000 |
| H | 0.197958000000 | 1.944273000000 | -2.377270000000 |
| H | -0.939389000000 | 3.300192000000 | -2.330124000000 |
| H | 0.620889000000 | 3.469238000000 | -3.190992000000 |
| C | 0.033635000000 | 3.956800000000 | 2.709908000000 |
| H | -0.730311000000 | 4.744197000000 | 2.815680000000 |
| H | -0.457949000000 | 2.987526000000 | 2.868218000000 |
| H | 0.771802000000 | 4.098428000000 | 3.512117000000 |
| C | 2.811262000000 | 0.502835000000 | -1.821336000000 |
| H | 2.494982000000 | -0.307379000000 | -2.496184000000 |
| H | 2.035911000000 | 0.599455000000 | -1.043898000000 |
| H | 2.844268000000 | 1.445090000000 | -2.385898000000 |
| C | 5.839609000000 | -2.697936000000 | 0.650278000000 |
| H | 5.940852000000 | -3.503997000000 | -0.095225000000 |
| H | 6.752747000000 | -2.691176000000 | 1.262775000000 |
| H | 4.984975000000 | -2.955826000000 | 1.294897000000 |
| C | 0.400477000000 | -4.364672000000 | 2.170210000000 |
| H | 0.950018000000 | -3.599548000000 | 2.743504000000 |
| H | -0.069888000000 | -5.054968000000 | 2.885653000000 |
| H | 1.149538000000 | -4.913066000000 | 1.579456000000 |
| C | -0.912911000000 | -1.809111000000 | -2.040291000000 |
| H | -1.769469000000 | -1.205836000000 | -2.366424000000 |
| H | -0.053662000000 | -1.138974000000 | -1.891081000000 |
| H | -0.667269000000 | -2.506032000000 | -2.857651000000 |

**INT1**

$N_{imag}$ **=** 0
$SP_{M062X}$ **=** -3058.828302
$G_{M062X}$ **=** -3058.146082

| | | | |
|---|---|---|---|
| P | 0.516989000000 | -0.644708000000 | -2.032707000000 |
| P | 0.196285000000 | 0.899629000000 | 0.678928000000 |
| P | 2.247907000000 | 0.489411000000 | -0.137035000000 |
| P | -2.309234000000 | -0.181147000000 | -0.748011000000 |
| N | 3.327453000000 | 1.767534000000 | -0.153478000000 |
| N | 3.043930000000 | -0.288995000000 | 1.122921000000 |
| N | -2.936270000000 | 0.032942000000 | 0.854836000000 |
| N | -2.476503000000 | -1.883140000000 | -0.592867000000 |
| C | 2.085560000000 | -0.318310000000 | -1.656683000000 |
| C | -0.506020000000 | 0.038726000000 | -0.552247000000 |
| C | 4.257536000000 | 1.757894000000 | 0.979782000000 |
| H | 3.886166000000 | 2.391328000000 | 1.805816000000 |
| H | 5.231127000000 | 2.156033000000 | 0.658207000000 |
| C | 4.357842000000 | 0.290433000000 | 1.411642000000 |
| H | 5.149845000000 | -0.231042000000 | 0.845685000000 |
| H | 4.578950000000 | 0.193361000000 | 2.484189000000 |
| C | 3.442156000000 | 2.710961000000 | -1.221003000000 |
| C | 4.391274000000 | 2.476016000000 | -2.234171000000 |
| C | 4.506963000000 | 3.413639000000 | -3.264687000000 |

| | | | |
|---|---|---|---|
| H | 5.236270000000 | 3.243069000000 | -4.062069000000 |
| C | 3.699134000000 | 4.550784000000 | -3.288908000000 |
| H | 3.800320000000 | 5.274250000000 | -4.103142000000 |
| C | 2.763381000000 | 4.766173000000 | -2.279962000000 |
| H | 2.131437000000 | 5.659120000000 | -2.299633000000 |
| C | 2.620746000000 | 3.850478000000 | -1.230107000000 |
| C | 2.369699000000 | -1.231120000000 | 1.958400000000 |
| C | 2.283093000000 | -2.571359000000 | 1.539947000000 |
| C | 1.520549000000 | -3.459545000000 | 2.306524000000 |
| H | 1.437035000000 | -4.502182000000 | 1.986820000000 |
| C | 0.873805000000 | -3.030181000000 | 3.464457000000 |
| H | 0.272263000000 | -3.733846000000 | 4.047096000000 |
| C | 1.007391000000 | -1.709853000000 | 3.890585000000 |
| H | 0.514907000000 | -1.377397000000 | 4.809185000000 |
| C | 1.763789000000 | -0.792456000000 | 3.152408000000 |
| C | -3.325100000000 | -1.221712000000 | 1.500943000000 |
| H | -4.406719000000 | -1.414064000000 | 1.362627000000 |
| H | -3.120331000000 | -1.196750000000 | 2.581331000000 |
| C | -2.493808000000 | -2.295253000000 | 0.807618000000 |
| H | -1.480992000000 | -2.328182000000 | 1.251307000000 |
| H | -2.948608000000 | -3.292654000000 | 0.898550000000 |
| C | -3.385285000000 | 1.278164000000 | 1.374515000000 |
| C | -4.334596000000 | 2.054936000000 | 0.671992000000 |
| C | -4.711522000000 | 3.299740000000 | 1.188827000000 |
| H | -5.441372000000 | 3.901308000000 | 0.638284000000 |
| C | -4.195626000000 | 3.765488000000 | 2.395266000000 |
| H | -4.502478000000 | 4.739387000000 | 2.787301000000 |
| C | -3.300543000000 | 2.971756000000 | 3.107410000000 |
| H | -2.902436000000 | 3.322596000000 | 4.064785000000 |
| C | -2.884627000000 | 1.727995000000 | 2.618742000000 |
| C | -2.474301000000 | -2.800352000000 | -1.676553000000 |
| C | -3.508948000000 | -2.738451000000 | -2.634250000000 |
| C | -3.489102000000 | -3.633266000000 | -3.708952000000 |
| H | -4.288752000000 | -3.584330000000 | -4.454401000000 |
| C | -2.483693000000 | -4.590888000000 | -3.827862000000 |
| H | -2.483124000000 | -5.285998000000 | -4.672286000000 |
| C | -1.481793000000 | -4.659847000000 | -2.862860000000 |
| H | -0.686794000000 | -5.406527000000 | -2.952129000000 |
| C | -1.456198000000 | -3.769717000000 | -1.783518000000 |
| C | -4.634557000000 | -1.745831000000 | -2.494404000000 |
| H | -4.301440000000 | -0.721170000000 | -2.723660000000 |
| H | -5.461714000000 | -1.997145000000 | -3.173843000000 |
| H | -5.015316000000 | -1.734619000000 | -1.461441000000 |
| C | -0.347100000000 | -3.856053000000 | -0.765322000000 |
| H | 0.525977000000 | -4.373872000000 | -1.186845000000 |
| H | -0.026655000000 | -2.859832000000 | -0.431042000000 |
| H | -0.665573000000 | -4.409168000000 | 0.133500000000 |
| C | 3.020740000000 | -3.040272000000 | 0.310812000000 |
| H | 2.892678000000 | -4.123182000000 | 0.171952000000 |
| H | 2.672034000000 | -2.525153000000 | -0.596069000000 |
| H | 4.097393000000 | -2.826300000000 | 0.403431000000 |
| C | 1.943072000000 | 0.619047000000 | 3.653726000000 |
| H | 1.891516000000 | 1.355258000000 | 2.839837000000 |
| H | 1.170019000000 | 0.870272000000 | 4.392917000000 |
| H | 2.924501000000 | 0.737167000000 | 4.142508000000 |
| C | -1.939340000000 | 0.899751000000 | 3.455715000000 |
| H | -2.497361000000 | 0.287988000000 | 4.185431000000 |

| | | | |
|---|---|---|---|
| H | -1.331366000000 | 0.223515000000 | 2.842144000000 |
| H | -1.261416000000 | 1.552439000000 | 4.025499000000 |
| C | -4.980074000000 | 1.562464000000 | -0.600410000000 |
| H | -5.933049000000 | 2.084744000000 | -0.769698000000 |
| H | -4.338135000000 | 1.730745000000 | -1.479134000000 |
| H | -5.178219000000 | 0.482216000000 | -0.551923000000 |
| C | 1.589804000000 | 4.076940000000 | -0.152164000000 |
| H | 1.340252000000 | 5.144590000000 | -0.068157000000 |
| H | 0.656264000000 | 3.537682000000 | -0.384223000000 |
| H | 1.937277000000 | 3.717566000000 | 0.827138000000 |
| C | 5.242322000000 | 1.231795000000 | -2.212073000000 |
| H | 4.599400000000 | 0.338481000000 | -2.175133000000 |
| H | 5.875681000000 | 1.178913000000 | -3.109039000000 |
| H | 5.902912000000 | 1.208990000000 | -1.330184000000 |

**INT1A**

$N_{imag}$ **=** 0
$SP_{M062X}$ **=** -3058.803891
$G_{M062X}$ **=** -3058.123003

| | | | |
|---|---|---|---|
| P | -1.546998000000 | -0.864059000000 | 2.422883000000 |
| P | 0.455695000000 | 0.112037000000 | 2.573896000000 |
| P | -1.275119000000 | 1.281533000000 | -0.030364000000 |
| P | 1.774611000000 | -1.734076000000 | 0.320275000000 |
| N | -0.972182000000 | 2.931064000000 | 0.333839000000 |
| N | -2.967954000000 | 1.594661000000 | -0.059968000000 |
| N | 3.126604000000 | -1.935912000000 | -0.631593000000 |
| N | 0.949163000000 | -3.000567000000 | -0.400329000000 |
| C | -1.030119000000 | 0.437755000000 | 1.548172000000 |
| C | 1.540387000000 | -0.734975000000 | 1.478500000000 |
| C | -2.105339000000 | 3.527144000000 | 1.034244000000 |
| H | -2.066304000000 | 4.624237000000 | 0.957282000000 |
| H | -2.127032000000 | 3.252996000000 | 2.104422000000 |
| C | -3.340279000000 | 2.950581000000 | 0.335849000000 |
| H | -3.608365000000 | 3.554168000000 | -0.552889000000 |
| H | -4.208900000000 | 2.946997000000 | 1.016106000000 |
| C | 0.326652000000 | 3.503133000000 | 0.255398000000 |
| C | 1.025221000000 | 3.912334000000 | 1.410992000000 |
| C | 2.317787000000 | 4.431258000000 | 1.268122000000 |
| H | 2.864527000000 | 4.735988000000 | 2.165573000000 |
| C | 2.920597000000 | 4.540137000000 | 0.017620000000 |
| H | 3.937370000000 | 4.933128000000 | -0.071073000000 |
| C | 2.215072000000 | 4.151993000000 | -1.119016000000 |
| H | 2.670508000000 | 4.256270000000 | -2.108809000000 |
| C | 0.914728000000 | 3.646684000000 | -1.021045000000 |
| C | -3.919276000000 | 0.735977000000 | -0.675297000000 |
| C | -4.861846000000 | 0.058429000000 | 0.123121000000 |
| C | -5.791933000000 | -0.786015000000 | -0.497074000000 |
| H | -6.520147000000 | -1.320050000000 | 0.121404000000 |
| C | -5.791155000000 | -0.960795000000 | -1.878272000000 |
| H | -6.518070000000 | -1.629852000000 | -2.347896000000 |
| C | -4.864968000000 | -0.272798000000 | -2.663333000000 |
| H | -4.873151000000 | -0.392728000000 | -3.751023000000 |
| C | -3.932331000000 | 0.589929000000 | -2.079221000000 |
| C | 3.123548000000 | -3.201276000000 | -1.361642000000 |

| | | | |
|---|---|---|---|
| H | 3.645016000000 | -3.990967000000 | -0.792668000000 |
| H | 3.631517000000 | -3.076901000000 | -2.328868000000 |
| C | 1.641718000000 | -3.567565000000 | -1.554683000000 |
| H | 1.259307000000 | -3.142990000000 | -2.498137000000 |
| H | 1.503313000000 | -4.659048000000 | -1.583705000000 |
| C | 4.176109000000 | -0.972958000000 | -0.692897000000 |
| C | 5.407937000000 | -1.242879000000 | -0.074220000000 |
| C | 6.415715000000 | -0.275542000000 | -0.150912000000 |
| H | 7.379946000000 | -0.470173000000 | 0.327996000000 |
| C | 6.193156000000 | 0.934471000000 | -0.806698000000 |
| H | 6.987072000000 | 1.685635000000 | -0.849919000000 |
| C | 4.957508000000 | 1.193619000000 | -1.397979000000 |
| H | 4.779044000000 | 2.147623000000 | -1.902128000000 |
| C | 3.931205000000 | 0.244331000000 | -1.354377000000 |
| C | -0.427470000000 | -3.236715000000 | -0.098298000000 |
| C | -0.739359000000 | -3.974025000000 | 1.057566000000 |
| C | -2.086589000000 | -4.172267000000 | 1.376574000000 |
| H | -2.343825000000 | -4.740136000000 | 2.275414000000 |
| C | -3.093825000000 | -3.637814000000 | 0.574448000000 |
| H | -4.143967000000 | -3.784502000000 | 0.843090000000 |
| C | -2.767433000000 | -2.896788000000 | -0.559382000000 |
| H | -3.557223000000 | -2.451443000000 | -1.169719000000 |
| C | -1.430725000000 | -2.685040000000 | -0.913863000000 |
| C | -4.896760000000 | 0.243103000000 | 1.619085000000 |
| H | -5.684726000000 | 0.961249000000 | 1.903217000000 |
| H | -5.117932000000 | -0.708675000000 | 2.125305000000 |
| H | -3.938894000000 | 0.625310000000 | 1.992751000000 |
| C | -2.966761000000 | 1.368136000000 | -2.937055000000 |
| H | -2.966614000000 | 2.433267000000 | -2.661945000000 |
| H | -1.934686000000 | 1.007507000000 | -2.808759000000 |
| H | -3.231436000000 | 1.280719000000 | -4.000666000000 |
| C | 0.152205000000 | 3.280219000000 | -2.268014000000 |
| H | 0.084624000000 | 2.187952000000 | -2.392211000000 |
| H | -0.879457000000 | 3.658275000000 | -2.217710000000 |
| H | 0.643771000000 | 3.694863000000 | -3.160195000000 |
| C | 0.421370000000 | 3.795897000000 | 2.788918000000 |
| H | -0.325276000000 | 4.585954000000 | 2.970795000000 |
| H | -0.071665000000 | 2.824260000000 | 2.924809000000 |
| H | 1.201922000000 | 3.889697000000 | 3.557231000000 |
| C | 2.589579000000 | 0.525393000000 | -1.981657000000 |
| H | 2.233360000000 | -0.335291000000 | -2.568784000000 |
| H | 1.832541000000 | 0.733852000000 | -1.207032000000 |
| H | 2.642340000000 | 1.404390000000 | -2.638554000000 |
| C | 5.622925000000 | -2.533620000000 | 0.675268000000 |
| H | 5.781472000000 | -3.379660000000 | -0.013670000000 |
| H | 6.505622000000 | -2.464120000000 | 1.326633000000 |
| H | 4.746379000000 | -2.772099000000 | 1.297496000000 |
| C | 0.360245000000 | -4.501626000000 | 1.943896000000 |
| H | 0.885154000000 | -3.671177000000 | 2.444773000000 |
| H | -0.046580000000 | -5.166289000000 | 2.719192000000 |
| H | 1.112214000000 | -5.055860000000 | 1.361273000000 |
| C | -1.089481000000 | -1.866665000000 | -2.132736000000 |
| H | -1.964940000000 | -1.289788000000 | -2.456465000000 |
| H | -0.269548000000 | -1.162403000000 | -1.925777000000 |
| H | -0.783132000000 | -2.509384000000 | -2.974116000000 |

**INTA**

$N_{imag}$ **=** 0
$SP_{M062X}$ **=** -3058.824270
$G_{M062X}$ **=** -3058.144372

| | | | |
|---|---|---|---|
| P | -0.074758000000 | -0.318266000000 | 1.376877000000 |
| P | 0.065247000000 | -0.318942000000 | -1.285079000000 |
| P | -2.944816000000 | -0.164862000000 | -0.498050000000 |
| P | 2.937753000000 | -0.118531000000 | 0.536367000000 |
| N | -3.373184000000 | 1.224391000000 | 0.432356000000 |
| N | -3.605287000000 | -1.183435000000 | 0.724947000000 |
| N | 3.191977000000 | 1.287076000000 | -0.451045000000 |
| N | 3.645266000000 | -1.097407000000 | -0.676559000000 |
| C | -1.172908000000 | -0.297339000000 | -0.162979000000 |
| C | 1.162295000000 | -0.315100000000 | 0.254364000000 |
| C | -3.493934000000 | 0.913269000000 | 1.853592000000 |
| H | -4.133350000000 | 1.660130000000 | 2.349014000000 |
| H | -2.520669000000 | 0.902286000000 | 2.376455000000 |
| C | -4.116556000000 | -0.485007000000 | 1.900816000000 |
| H | -5.221062000000 | -0.421865000000 | 1.876837000000 |
| H | -3.832557000000 | -1.009686000000 | 2.829417000000 |
| C | -3.303509000000 | 2.546726000000 | -0.083491000000 |
| C | -2.332038000000 | 3.464202000000 | 0.371280000000 |
| C | -2.306857000000 | 4.752919000000 | -0.176534000000 |
| H | -1.550250000000 | 5.460652000000 | 0.176227000000 |
| C | -3.208586000000 | 5.133126000000 | -1.166753000000 |
| H | -3.169912000000 | 6.140945000000 | -1.589855000000 |
| C | -4.162990000000 | 4.220511000000 | -1.612236000000 |
| H | -4.883953000000 | 4.514777000000 | -2.381031000000 |
| C | -4.232327000000 | 2.930462000000 | -1.076930000000 |
| C | -4.015652000000 | -2.523530000000 | 0.461205000000 |
| C | -3.203293000000 | -3.582740000000 | 0.909122000000 |
| C | -3.615641000000 | -4.899081000000 | 0.672917000000 |
| H | -2.990055000000 | -5.726334000000 | 1.021749000000 |
| C | -4.806687000000 | -5.161511000000 | -0.001938000000 |
| H | -5.118816000000 | -6.194679000000 | -0.180348000000 |
| C | -5.597963000000 | -4.106175000000 | -0.452833000000 |
| C | -5.216507000000 | -2.778295000000 | -0.229346000000 |
| C | 3.874622000000 | 1.023708000000 | -1.714227000000 |
| H | 4.954694000000 | 1.256992000000 | -1.637391000000 |
| H | 3.458331000000 | 1.641823000000 | -2.527443000000 |
| C | 3.666843000000 | -0.467343000000 | -1.991937000000 |
| H | 2.724366000000 | -0.622521000000 | -2.551900000000 |
| H | 4.488266000000 | -0.885562000000 | -2.593796000000 |
| C | 3.161679000000 | 2.611087000000 | 0.075763000000 |
| C | 4.152596000000 | 3.045524000000 | 0.978899000000 |
| C | 4.088003000000 | 4.351680000000 | 1.478988000000 |
| H | 4.854599000000 | 4.690858000000 | 2.182370000000 |
| C | 3.073566000000 | 5.220181000000 | 1.081976000000 |
| H | 3.039347000000 | 6.240084000000 | 1.475923000000 |
| C | 2.106974000000 | 4.787695000000 | 0.175368000000 |
| H | 1.311290000000 | 5.467763000000 | -0.144156000000 |
| C | 2.133329000000 | 3.484551000000 | -0.334097000000 |
| C | 4.115579000000 | -2.424384000000 | -0.457993000000 |
| C | 5.410876000000 | -2.608358000000 | 0.062449000000 |
| C | 5.880369000000 | -3.910624000000 | 0.265582000000 |

| | | | |
|---|---|---|---|
| H | 6.886294000000 | -4.059896000000 | 0.669485000000 |
| C | 5.083670000000 | -5.010715000000 | -0.047882000000 |
| H | 5.463090000000 | -6.024477000000 | 0.110057000000 |
| C | 3.802208000000 | -4.818080000000 | -0.561559000000 |
| H | 3.174085000000 | -5.680935000000 | -0.802964000000 |
| C | 3.298872000000 | -3.528369000000 | -0.767927000000 |
| C | -6.059615000000 | -1.640243000000 | -0.747136000000 |
| H | -7.009247000000 | -2.012060000000 | -1.157762000000 |
| H | -6.287145000000 | -0.909082000000 | 0.042495000000 |
| H | -5.530468000000 | -1.096604000000 | -1.545536000000 |
| C | -1.893717000000 | -3.301107000000 | 1.602455000000 |
| H | -2.004080000000 | -2.542823000000 | 2.391252000000 |
| H | -1.479990000000 | -4.215944000000 | 2.050663000000 |
| H | -1.154035000000 | -2.914361000000 | 0.881350000000 |
| C | -5.297261000000 | 1.973546000000 | -1.547698000000 |
| H | -4.873273000000 | 1.198167000000 | -2.205817000000 |
| H | -5.755769000000 | 1.450869000000 | -0.695650000000 |
| H | -6.080124000000 | 2.506105000000 | -2.106744000000 |
| C | -1.311320000000 | 3.090868000000 | 1.415649000000 |
| H | -0.472192000000 | 3.800495000000 | 1.413391000000 |
| H | -1.742841000000 | 3.080100000000 | 2.429092000000 |
| H | -0.901568000000 | 2.088616000000 | 1.226651000000 |
| C | 1.055944000000 | 3.021269000000 | -1.280525000000 |
| H | 1.468500000000 | 2.534228000000 | -2.175904000000 |
| H | 0.399755000000 | 2.289603000000 | -0.786173000000 |
| H | 0.423137000000 | 3.861654000000 | -1.599607000000 |
| C | 5.264766000000 | 2.121763000000 | 1.408068000000 |
| H | 6.066032000000 | 2.683036000000 | 1.909909000000 |
| H | 4.893357000000 | 1.355159000000 | 2.107074000000 |
| H | 5.696375000000 | 1.589199000000 | 0.548600000000 |
| C | 1.894375000000 | -3.329304000000 | -1.282157000000 |
| H | 1.437113000000 | -4.290270000000 | -1.557686000000 |
| H | 1.263490000000 | -2.863259000000 | -0.507710000000 |
| H | 1.869137000000 | -2.674153000000 | -2.165844000000 |
| C | 6.266187000000 | -1.414112000000 | 0.400566000000 |
| H | 5.834515000000 | -0.851375000000 | 1.243125000000 |
| H | 7.283277000000 | -1.724881000000 | 0.679719000000 |
| H | 6.330744000000 | -0.718162000000 | -0.449694000000 |
| H | -6.529910000000 | -4.310808000000 | -0.988535000000 |

**TS$_{C-H}$**

***N*$_{imag}$ =** 0, 1267.4 cm$^{-1}$
***SP*$_{M062X}$ =** -3058.779256
***G*$_{M062X}$ =** -3058.101254

| | | | |
|---|---|---|---|
| P | -0.326430000000 | -0.134615000000 | 2.148944000000 |
| P | -0.287365000000 | 0.390821000000 | -0.937833000000 |
| P | -2.187010000000 | 0.647986000000 | 0.090941000000 |
| P | 2.260358000000 | -0.394104000000 | 0.649668000000 |
| N | -2.651230000000 | 2.280285000000 | 0.186847000000 |
| N | -3.490722000000 | 0.255597000000 | -0.886198000000 |
| N | 2.853365000000 | -0.082875000000 | -0.940996000000 |
| N | 2.071679000000 | -2.081896000000 | 0.273555000000 |
| C | -1.976403000000 | -0.023123000000 | 1.703142000000 |
| C | 0.482011000000 | 0.094776000000 | 0.551414000000 |

| | | | |
|---|---|---|---|
| C | -3.612216000000 | 2.636616000000 | -0.857700000000 |
| H | -3.116299000000 | 2.937757000000 | -1.797025000000 |
| H | -4.234156000000 | 3.472795000000 | -0.506395000000 |
| C | -4.435512000000 | 1.361735000000 | -1.069194000000 |
| H | -5.262132000000 | 1.299435000000 | -0.339202000000 |
| H | -4.864316000000 | 1.318844000000 | -2.080238000000 |
| C | -2.031348000000 | 3.166403000000 | 1.095478000000 |
| C | -2.272448000000 | 2.924399000000 | 2.471319000000 |
| C | -1.501333000000 | 3.635773000000 | 3.414380000000 |
| H | -1.639251000000 | 3.430310000000 | 4.479596000000 |
| C | -0.609457000000 | 4.613686000000 | 2.995411000000 |
| H | -0.032735000000 | 5.176690000000 | 3.734991000000 |
| C | -0.435088000000 | 4.882784000000 | 1.631792000000 |
| H | 0.278400000000 | 5.648781000000 | 1.314302000000 |
| C | -1.119658000000 | 4.145197000000 | 0.662650000000 |
| C | -3.223934000000 | 1.901671000000 | 2.846917000000 |
| H | -3.376449000000 | 1.747351000000 | 3.922188000000 |
| H | -4.138427000000 | 1.841015000000 | 2.246319000000 |
| C | -3.539202000000 | -0.962831000000 | -1.632589000000 |
| C | -3.982448000000 | -2.136379000000 | -0.996167000000 |
| C | -3.971829000000 | -3.332078000000 | -1.722875000000 |
| H | -4.308878000000 | -4.253004000000 | -1.238111000000 |
| C | -3.541875000000 | -3.358160000000 | -3.048128000000 |
| H | -3.535184000000 | -4.301390000000 | -3.601900000000 |
| C | -3.126034000000 | -2.182647000000 | -3.671136000000 |
| H | -2.793065000000 | -2.203160000000 | -4.713023000000 |
| C | -3.119841000000 | -0.967766000000 | -2.977169000000 |
| C | 2.850288000000 | -1.263086000000 | -1.813147000000 |
| H | 3.872252000000 | -1.677464000000 | -1.894836000000 |
| H | 2.509091000000 | -1.004389000000 | -2.829952000000 |
| C | 1.903494000000 | -2.271102000000 | -1.163507000000 |
| H | 0.873406000000 | -2.070363000000 | -1.506961000000 |
| H | 2.159502000000 | -3.306469000000 | -1.436327000000 |
| C | 3.880092000000 | 0.890901000000 | -1.180955000000 |
| C | 5.161150000000 | 0.745027000000 | -0.611552000000 |
| C | 6.143491000000 | 1.702963000000 | -0.893037000000 |
| H | 7.136622000000 | 1.590774000000 | -0.447400000000 |
| C | 5.874975000000 | 2.778353000000 | -1.734617000000 |
| H | 6.653090000000 | 3.516615000000 | -1.949396000000 |
| C | 4.611983000000 | 2.903838000000 | -2.310079000000 |
| H | 4.396572000000 | 3.742286000000 | -2.979404000000 |
| C | 3.604370000000 | 1.970302000000 | -2.044940000000 |
| C | 1.618482000000 | -3.035740000000 | 1.234135000000 |
| C | 2.504011000000 | -3.389792000000 | 2.277994000000 |
| C | 2.093757000000 | -4.308556000000 | 3.248278000000 |
| H | 2.786551000000 | -4.571544000000 | 4.053272000000 |
| C | 0.834637000000 | -4.899336000000 | 3.189356000000 |
| H | 0.524852000000 | -5.621213000000 | 3.950280000000 |
| C | -0.026420000000 | -4.558531000000 | 2.151815000000 |
| H | -1.022494000000 | -5.008687000000 | 2.101924000000 |
| C | 0.334104000000 | -3.623696000000 | 1.172018000000 |
| H | -2.741906000000 | 0.582932000000 | 2.478360000000 |
| C | 3.891604000000 | -2.808840000000 | 2.348518000000 |
| H | 3.865015000000 | -1.727304000000 | 2.552212000000 |
| H | 4.473645000000 | -3.293802000000 | 3.145212000000 |
| H | 4.419312000000 | -2.944635000000 | 1.393369000000 |
| C | -0.689489000000 | -3.288159000000 | 0.112436000000 |

| | | | |
|---|---|---|---|
| H | -1.681746000000 | -3.639668000000 | 0.426518000000 |
| H | -0.756954000000 | -2.208160000000 | -0.063346000000 |
| H | -0.460812000000 | -3.768538000000 | -0.852446000000 |
| C | -2.690263000000 | 0.301245000000 | -3.670607000000 |
| H | -2.077023000000 | 0.935833000000 | -3.016007000000 |
| H | -2.104304000000 | 0.072229000000 | -4.571859000000 |
| H | -3.564002000000 | 0.896622000000 | -3.984326000000 |
| C | -4.454647000000 | -2.106682000000 | 0.435231000000 |
| H | -4.940111000000 | -3.056093000000 | 0.702917000000 |
| H | -3.615354000000 | -1.933498000000 | 1.127251000000 |
| H | -5.171205000000 | -1.286779000000 | 0.597655000000 |
| C | -0.855012000000 | 4.347906000000 | -0.807693000000 |
| H | -1.698979000000 | 4.851803000000 | -1.306727000000 |
| H | 0.044331000000 | 4.960452000000 | -0.962784000000 |
| H | -0.704563000000 | 3.377663000000 | -1.306610000000 |
| C | 2.242023000000 | 2.139464000000 | -2.665921000000 |
| H | 1.896259000000 | 1.216212000000 | -3.153067000000 |
| H | 1.492352000000 | 2.383065000000 | -1.899068000000 |
| H | 2.253219000000 | 2.947814000000 | -3.411672000000 |
| C | 5.490478000000 | -0.406487000000 | 0.303236000000 |
| H | 6.575004000000 | -0.587193000000 | 0.325727000000 |
| H | 5.161339000000 | -0.194279000000 | 1.332323000000 |
| H | 4.988537000000 | -1.331400000000 | -0.009089000000 |

**TS2**

***N*<sub></sub>** 

$\boldsymbol{N_{imag}}$ **=** 0, -436.9 cm$^{-1}$
$\boldsymbol{SP_{M062X}}$ **=** -4588.212265
$\boldsymbol{G_{M062X}}$ **=** -4587.174066

| | | | |
|---|---|---|---|
| P | 0.025867000000 | -1.942613000000 | 2.315973000000 |
| P | -0.606238000000 | -1.738415000000 | -0.308288000000 |
| P | -2.381438000000 | 0.743217000000 | -0.133030000000 |
| P | 2.508387000000 | -0.286047000000 | 0.756710000000 |
| P | 0.398639000000 | 1.120417000000 | -1.392640000000 |
| P | -3.127524000000 | -1.174521000000 | 0.798699000000 |
| N | 3.713928000000 | -1.261014000000 | 1.485396000000 |
| N | 2.757103000000 | 0.933584000000 | 1.936782000000 |
| N | -4.669338000000 | -1.526519000000 | 0.177828000000 |
| N | -3.787967000000 | -0.878051000000 | 2.336887000000 |
| N | 1.216490000000 | 0.055139000000 | -2.467209000000 |
| N | -0.432610000000 | 1.874087000000 | -2.743077000000 |
| C | 1.026586000000 | -1.087487000000 | 1.467202000000 |
| C | -1.907284000000 | -2.290447000000 | 0.578062000000 |
| C | -0.932501000000 | 0.092461000000 | -0.645684000000 |
| C | 3.767423000000 | -1.026487000000 | 2.922022000000 |
| H | 4.759950000000 | -1.289092000000 | 3.315661000000 |
| H | 3.020694000000 | -1.653742000000 | 3.445621000000 |
| C | 3.448311000000 | 0.469560000000 | 3.139002000000 |
| H | 2.830218000000 | 0.603400000000 | 4.045053000000 |
| H | 4.374779000000 | 1.054786000000 | 3.279611000000 |
| C | 4.596040000000 | -2.164446000000 | 0.824287000000 |
| C | 4.112028000000 | -3.388794000000 | 0.320301000000 |
| C | 4.995748000000 | -4.252323000000 | -0.334448000000 |
| H | 4.616542000000 | -5.200815000000 | -0.726975000000 |
| C | 6.343089000000 | -3.926190000000 | -0.478623000000 |

| | | | |
|---|---|---|---|
| H | 7.023716000000 | -4.612011000000 | -0.991551000000 |
| C | 6.817316000000 | -2.724217000000 | 0.038273000000 |
| H | 7.873576000000 | -2.459317000000 | -0.071659000000 |
| C | 5.958659000000 | -1.831545000000 | 0.690681000000 |
| C | 2.473255000000 | 2.322420000000 | 1.777814000000 |
| C | 1.244010000000 | 2.835478000000 | 2.233883000000 |
| C | 0.997980000000 | 4.208805000000 | 2.129414000000 |
| H | 0.043063000000 | 4.607005000000 | 2.485767000000 |
| C | 1.945426000000 | 5.063214000000 | 1.569726000000 |
| H | 1.738706000000 | 6.134224000000 | 1.487181000000 |
| C | 3.151596000000 | 4.546338000000 | 1.103381000000 |
| H | 3.894620000000 | 5.210797000000 | 0.651974000000 |
| C | 3.433822000000 | 3.178661000000 | 1.205118000000 |
| C | -5.759658000000 | -1.138576000000 | 1.068623000000 |
| H | -6.624228000000 | -1.800244000000 | 0.906616000000 |
| H | -6.082958000000 | -0.095807000000 | 0.905155000000 |
| C | -5.185001000000 | -1.285565000000 | 2.470149000000 |
| H | -5.700824000000 | -0.631092000000 | 3.190116000000 |
| H | -5.271536000000 | -2.328887000000 | 2.823024000000 |
| C | -4.928172000000 | -1.966446000000 | -1.154937000000 |
| C | -4.709436000000 | -3.320789000000 | -1.481582000000 |
| C | -4.949703000000 | -3.737281000000 | -2.796051000000 |
| H | -4.771903000000 | -4.783693000000 | -3.061342000000 |
| C | -5.435832000000 | -2.849069000000 | -3.753684000000 |
| H | -5.622052000000 | -3.192180000000 | -4.775532000000 |
| C | -5.711060000000 | -1.530491000000 | -3.399211000000 |
| H | -6.121873000000 | -0.838044000000 | -4.140253000000 |
| C | -5.466011000000 | -1.070890000000 | -2.099830000000 |
| C | -3.024231000000 | -0.657366000000 | 3.523333000000 |
| C | -2.543039000000 | -1.747281000000 | 4.274902000000 |
| C | -1.823623000000 | -1.482816000000 | 5.446165000000 |
| H | -1.429310000000 | -2.321590000000 | 6.027594000000 |
| C | -1.605287000000 | -0.175019000000 | 5.873728000000 |
| H | -1.040773000000 | 0.013906000000 | 6.791422000000 |
| C | -2.119954000000 | 0.891941000000 | 5.139988000000 |
| H | -1.968124000000 | 1.918267000000 | 5.486550000000 |
| C | -2.837428000000 | 0.667531000000 | 3.960335000000 |
| C | 0.461795000000 | -0.091072000000 | -3.716035000000 |
| H | -0.386241000000 | -0.785533000000 | -3.572011000000 |
| H | 1.110982000000 | -0.491298000000 | -4.505907000000 |
| C | -0.041732000000 | 1.311699000000 | -4.034481000000 |
| H | 0.770048000000 | 1.908190000000 | -4.493477000000 |
| H | -0.875978000000 | 1.300168000000 | -4.744650000000 |
| C | 2.634154000000 | -0.116539000000 | -2.523781000000 |
| C | 3.125982000000 | -1.427853000000 | -2.695855000000 |
| C | 4.507066000000 | -1.640846000000 | -2.726624000000 |
| H | 4.884326000000 | -2.661504000000 | -2.828168000000 |
| C | 5.398572000000 | -0.580754000000 | -2.593312000000 |
| H | 6.476445000000 | -0.764170000000 | -2.597156000000 |
| C | 4.904017000000 | 0.713828000000 | -2.462947000000 |
| C | 3.527316000000 | 0.971146000000 | -2.442537000000 |
| C | -1.524245000000 | 2.760632000000 | -2.590035000000 |
| C | -2.807796000000 | 2.420695000000 | -3.076982000000 |
| C | -3.865284000000 | 3.316036000000 | -2.878018000000 |
| H | -4.857450000000 | 3.053733000000 | -3.257078000000 |
| C | -3.683709000000 | 4.507324000000 | -2.181180000000 |
| H | -4.526256000000 | 5.185906000000 | -2.020114000000 |

| | | | |
|---|---|---|---|
| C | -2.418476000000 | 4.828998000000 | -1.694219000000 |
| H | -2.261632000000 | 5.769238000000 | -1.156716000000 |
| C | -1.327190000000 | 3.980765000000 | -1.904249000000 |
| C | 2.669478000000 | -3.780118000000 | 0.499326000000 |
| H | 2.501588000000 | -4.812351000000 | 0.159317000000 |
| H | 2.369586000000 | -3.700774000000 | 1.554284000000 |
| H | 1.992728000000 | -3.127030000000 | -0.068978000000 |
| C | 6.501605000000 | -0.539251000000 | 1.241416000000 |
| H | 6.847910000000 | -0.662822000000 | 2.281724000000 |
| H | 7.358433000000 | -0.188713000000 | 0.647182000000 |
| H | 5.729119000000 | 0.236018000000 | 1.240774000000 |
| C | 4.749561000000 | 2.639786000000 | 0.708991000000 |
| H | 5.263960000000 | 3.381951000000 | 0.081751000000 |
| H | 5.416378000000 | 2.391481000000 | 1.550207000000 |
| H | 4.608646000000 | 1.725304000000 | 0.117555000000 |
| C | 0.194653000000 | 1.920555000000 | 2.803653000000 |
| H | 0.618845000000 | 1.186998000000 | 3.503538000000 |
| H | -0.580026000000 | 2.498071000000 | 3.324509000000 |
| H | -0.291596000000 | 1.340499000000 | 2.004288000000 |
| C | -2.789363000000 | -3.171543000000 | 3.843484000000 |
| H | -2.096473000000 | -3.853178000000 | 4.357641000000 |
| H | -3.814672000000 | -3.487060000000 | 4.098208000000 |
| H | -2.655187000000 | -3.287999000000 | 2.757765000000 |
| C | -3.417380000000 | 1.822564000000 | 3.183222000000 |
| H | -2.872178000000 | 1.989543000000 | 2.241456000000 |
| H | -4.467358000000 | 1.634469000000 | 2.913278000000 |
| H | -3.364606000000 | 2.751108000000 | 3.769878000000 |
| C | -4.283077000000 | -4.313596000000 | -0.431326000000 |
| H | -4.330397000000 | -5.338916000000 | -0.826083000000 |
| H | -3.262127000000 | -4.097358000000 | -0.080566000000 |
| H | -4.940900000000 | -4.244459000000 | 0.450204000000 |
| C | -5.815216000000 | 0.348411000000 | -1.725533000000 |
| H | -5.002948000000 | 0.837613000000 | -1.166955000000 |
| H | -6.026107000000 | 0.947175000000 | -2.621637000000 |
| H | -6.718020000000 | 0.379234000000 | -1.095777000000 |
| C | -3.082600000000 | 1.108475000000 | -3.769103000000 |
| H | -2.519412000000 | 0.285977000000 | -3.304662000000 |
| H | -4.148515000000 | 0.863483000000 | -3.714118000000 |
| H | -2.819018000000 | 1.138918000000 | -4.838457000000 |
| C | 0.041799000000 | 4.381882000000 | -1.419282000000 |
| H | 0.041392000000 | 5.426310000000 | -1.075876000000 |
| H | 0.384340000000 | 3.756269000000 | -0.580528000000 |
| H | 0.786733000000 | 4.273314000000 | -2.221264000000 |
| C | 3.056719000000 | 2.400128000000 | -2.332108000000 |
| H | 2.217778000000 | 2.603316000000 | -3.012034000000 |
| H | 2.710762000000 | 2.638687000000 | -1.315885000000 |
| H | 3.874897000000 | 3.091555000000 | -2.581193000000 |
| C | 2.185632000000 | -2.596719000000 | -2.845923000000 |
| H | 2.695090000000 | -3.536941000000 | -2.589607000000 |
| H | 1.304330000000 | -2.477415000000 | -2.201538000000 |
| H | 1.822503000000 | -2.686743000000 | -3.883702000000 |
| H | 5.597346000000 | 1.557086000000 | -2.386390000000 |

**2M**

$N_{imag}$ = 0

***SP$_{M062x}$* =** -3058.904930
***G$_{M062x}$* =** -3058.217637

| | | | |
|---|---|---|---|
| P | 0.060142000000 | 0.085498000000 | 2.139963000000 |
| P | -0.373421000000 | 0.250122000000 | -0.974077000000 |
| P | -2.173560000000 | 0.282725000000 | 0.100512000000 |
| P | 2.425107000000 | 0.049866000000 | 0.492803000000 |
| N | -3.112167000000 | 1.686064000000 | 0.238652000000 |
| N | -3.433849000000 | -0.566340000000 | -0.615867000000 |
| N | 2.853824000000 | 0.577027000000 | -1.096919000000 |
| N | 2.612140000000 | -1.605555000000 | 0.010504000000 |
| C | -1.775635000000 | -0.115418000000 | 1.834598000000 |
| C | 0.586986000000 | 0.189042000000 | 0.535417000000 |
| C | -4.374463000000 | 1.625222000000 | -0.493879000000 |
| H | -4.275295000000 | 2.043962000000 | -1.510609000000 |
| H | -5.143808000000 | 2.198834000000 | 0.043775000000 |
| C | -4.721591000000 | 0.132027000000 | -0.557812000000 |
| H | -5.301341000000 | -0.177286000000 | 0.330889000000 |
| H | -5.313170000000 | -0.112658000000 | -1.452181000000 |
| C | -2.802629000000 | 2.644077000000 | 1.238081000000 |
| C | -2.565979000000 | 2.172044000000 | 2.541646000000 |
| C | -2.219985000000 | 3.082725000000 | 3.539954000000 |
| H | -2.019289000000 | 2.720906000000 | 4.552623000000 |
| C | -2.139447000000 | 4.445624000000 | 3.248803000000 |
| H | -1.874557000000 | 5.157621000000 | 4.035377000000 |
| C | -2.378760000000 | 4.899182000000 | 1.951949000000 |
| H | -2.291830000000 | 5.965711000000 | 1.723294000000 |
| C | -2.695647000000 | 4.006961000000 | 0.920511000000 |
| C | -3.256330000000 | -1.762502000000 | -1.378004000000 |
| C | -3.324017000000 | -3.012106000000 | -0.742104000000 |
| C | -3.127183000000 | -4.165090000000 | -1.513511000000 |
| H | -3.174678000000 | -5.145257000000 | -1.029472000000 |
| C | -2.873258000000 | -4.074243000000 | -2.878735000000 |
| H | -2.715979000000 | -4.982638000000 | -3.467262000000 |
| C | -2.827476000000 | -2.825221000000 | -3.499864000000 |
| H | -2.634797000000 | -2.754844000000 | -4.574353000000 |
| C | -3.023508000000 | -1.654432000000 | -2.763551000000 |
| C | 3.092825000000 | -0.518443000000 | -2.041179000000 |
| H | 4.179732000000 | -0.670065000000 | -2.182082000000 |
| H | 2.654140000000 | -0.294557000000 | -3.028530000000 |
| C | 2.444824000000 | -1.762508000000 | -1.432356000000 |
| H | 1.386521000000 | -1.808668000000 | -1.738172000000 |
| H | 2.945621000000 | -2.683151000000 | -1.771041000000 |
| C | 3.583840000000 | 1.790518000000 | -1.318982000000 |
| C | 4.880558000000 | 1.960974000000 | -0.792509000000 |
| C | 5.570756000000 | 3.151599000000 | -1.053032000000 |
| H | 6.575702000000 | 3.283507000000 | -0.640281000000 |
| C | 5.001882000000 | 4.152456000000 | -1.835185000000 |
| H | 5.553876000000 | 5.075655000000 | -2.034593000000 |
| C | 3.729053000000 | 3.966951000000 | -2.371363000000 |
| H | 3.279085000000 | 4.745677000000 | -2.994675000000 |
| C | 3.006912000000 | 2.794240000000 | -2.124382000000 |
| C | 2.462474000000 | -2.679552000000 | 0.933377000000 |
| C | 3.473848000000 | -2.867227000000 | 1.904330000000 |
| C | 3.352091000000 | -3.901811000000 | 2.836810000000 |
| H | 4.140590000000 | -4.035865000000 | 3.583581000000 |
| C | 2.261506000000 | -4.767110000000 | 2.810776000000 |

| | | | |
|---|---|---|---|
| H | 2.180216000000 | -5.578391000000 | 3.539896000000 |
| C | 1.276713000000 | -4.587761000000 | 1.844860000000 |
| H | 0.411550000000 | -5.257755000000 | 1.818608000000 |
| C | 1.347367000000 | -3.547888000000 | 0.907711000000 |
| H | -1.969032000000 | -1.196361000000 | 1.940833000000 |
| C | -2.675325000000 | 0.685145000000 | 2.793743000000 |
| H | -2.402082000000 | 0.457487000000 | 3.835020000000 |
| H | -3.724475000000 | 0.367837000000 | 2.657639000000 |
| C | -2.881265000000 | 4.472857000000 | -0.500803000000 |
| H | -3.942748000000 | 4.466062000000 | -0.798303000000 |
| H | -2.503346000000 | 5.496897000000 | -0.630485000000 |
| H | -2.341979000000 | 3.805151000000 | -1.191201000000 |
| C | 1.626670000000 | 2.625521000000 | -2.703813000000 |
| H | 1.529243000000 | 1.680603000000 | -3.258657000000 |
| H | 0.867551000000 | 2.588522000000 | -1.908714000000 |
| H | 1.387375000000 | 3.456518000000 | -3.383714000000 |
| C | 5.532353000000 | 0.895617000000 | 0.050837000000 |
| H | 6.625902000000 | 1.012409000000 | 0.048397000000 |
| H | 5.186224000000 | 0.955695000000 | 1.094270000000 |
| H | 5.286929000000 | -0.112062000000 | -0.309269000000 |
| C | -3.581414000000 | -3.132677000000 | 0.739733000000 |
| H | -2.631191000000 | -3.212513000000 | 1.293554000000 |
| H | -4.126452000000 | -2.263146000000 | 1.131549000000 |
| H | -4.162175000000 | -4.040424000000 | 0.959674000000 |
| C | -2.991112000000 | -0.304822000000 | -3.435996000000 |
| H | -3.972078000000 | 0.194051000000 | -3.379930000000 |
| H | -2.254999000000 | 0.363113000000 | -2.963404000000 |
| H | -2.725019000000 | -0.406532000000 | -4.497482000000 |
| C | 4.693504000000 | -1.984592000000 | 1.931984000000 |
| H | 4.434471000000 | -0.952423000000 | 2.212904000000 |
| H | 5.430642000000 | -2.364144000000 | 2.654123000000 |
| H | 5.162233000000 | -1.939372000000 | 0.938186000000 |
| C | 0.212037000000 | -3.398179000000 | -0.074786000000 |
| H | -0.640171000000 | -4.016574000000 | 0.238710000000 |
| H | -0.127019000000 | -2.356331000000 | -0.157492000000 |
| H | 0.493317000000 | -3.727820000000 | -1.087676000000 |

**3M**

***N*<sub></sub>** 

$\boldsymbol{N_{imag}}$ **=** 0
$\boldsymbol{SP_{M062X}}$ **=** -4588.318088
$\boldsymbol{G_{M062X}}$ **=** -4587.277422

| | | | |
|---|---|---|---|
| P | -0.143087000000 | -1.492220000000 | 2.130761000000 |
| P | -0.111148000000 | -1.484760000000 | -0.451177000000 |
| P | -2.459630000000 | 0.488059000000 | -0.515763000000 |
| P | 2.541426000000 | -0.129349000000 | 0.859674000000 |
| P | 0.284171000000 | 1.346851000000 | -1.601095000000 |
| P | -2.822503000000 | -1.387559000000 | 0.584896000000 |
| N | 3.555756000000 | -1.152880000000 | 1.808125000000 |
| N | 2.407544000000 | 0.967505000000 | 2.185244000000 |
| N | -3.933625000000 | -2.348907000000 | -0.246713000000 |
| N | -3.852707000000 | -1.191147000000 | 1.893502000000 |
| N | 0.784529000000 | 0.641599000000 | -3.085592000000 |
| N | -0.748104000000 | 2.443355000000 | -2.447747000000 |
| C | 0.916837000000 | -0.924921000000 | 0.940460000000 |

| | | | |
|---|---|---|---|
| C | -1.268471000000 | -2.017982000000 | 0.838463000000 |
| C | -0.877063000000 | 0.093374000000 | -0.919610000000 |
| C | 3.173098000000 | -1.067178000000 | 3.209583000000 |
| H | 3.982738000000 | -1.410902000000 | 3.866895000000 |
| H | 2.295104000000 | -1.712905000000 | 3.408380000000 |
| C | 2.809187000000 | 0.409323000000 | 3.472635000000 |
| H | 2.001329000000 | 0.476905000000 | 4.224641000000 |
| H | 3.679748000000 | 0.962908000000 | 3.871355000000 |
| C | 4.638834000000 | -1.924275000000 | 1.318095000000 |
| C | 4.405207000000 | -2.917587000000 | 0.340857000000 |
| C | 5.486807000000 | -3.646313000000 | -0.165056000000 |
| H | 5.297672000000 | -4.411400000000 | -0.924299000000 |
| C | 6.783049000000 | -3.426591000000 | 0.295714000000 |
| H | 7.617935000000 | -4.009616000000 | -0.103660000000 |
| C | 7.005224000000 | -2.456743000000 | 1.269769000000 |
| H | 8.021060000000 | -2.268138000000 | 1.631411000000 |
| C | 5.952559000000 | -1.690748000000 | 1.784406000000 |
| C | 2.245700000000 | 2.374734000000 | 2.037749000000 |
| C | 0.980644000000 | 2.955030000000 | 2.263058000000 |
| C | 0.847655000000 | 4.346152000000 | 2.187271000000 |
| H | -0.133650000000 | 4.797424000000 | 2.360671000000 |
| C | 1.940870000000 | 5.153106000000 | 1.879691000000 |
| H | 1.822077000000 | 6.239190000000 | 1.821566000000 |
| C | 3.180178000000 | 4.571157000000 | 1.624167000000 |
| H | 4.036631000000 | 5.199314000000 | 1.360290000000 |
| C | 3.349635000000 | 3.183269000000 | 1.697201000000 |
| C | -5.267095000000 | -2.337770000000 | 0.348285000000 |
| H | -5.755685000000 | -3.312707000000 | 0.196871000000 |
| H | -5.912256000000 | -1.557754000000 | -0.093963000000 |
| C | -5.031863000000 | -2.060717000000 | 1.835994000000 |
| H | -5.892458000000 | -1.546159000000 | 2.289735000000 |
| H | -4.860563000000 | -3.003134000000 | 2.383818000000 |
| C | -3.664273000000 | -2.778861000000 | -1.584164000000 |
| C | -2.942497000000 | -3.977950000000 | -1.760031000000 |
| C | -2.606414000000 | -4.372956000000 | -3.058372000000 |
| H | -2.039410000000 | -5.297032000000 | -3.203446000000 |
| C | -2.986975000000 | -3.607429000000 | -4.159392000000 |
| H | -2.707636000000 | -3.923321000000 | -5.168617000000 |
| C | -3.729547000000 | -2.444805000000 | -3.974175000000 |
| H | -4.042146000000 | -1.850954000000 | -4.838447000000 |
| C | -4.087783000000 | -2.015516000000 | -2.689509000000 |
| C | -3.446808000000 | -0.567987000000 | 3.116120000000 |
| C | -2.862302000000 | -1.344749000000 | 4.137183000000 |
| C | -2.458552000000 | -0.705375000000 | 5.313033000000 |
| H | -1.989219000000 | -1.296275000000 | 6.105030000000 |
| C | -2.637506000000 | 0.666741000000 | 5.479133000000 |
| H | -2.311547000000 | 1.152976000000 | 6.403073000000 |
| C | -3.234803000000 | 1.417155000000 | 4.470482000000 |
| H | -3.384955000000 | 2.492609000000 | 4.604344000000 |
| C | -3.649858000000 | 0.815735000000 | 3.276289000000 |
| C | -0.196055000000 | 0.878849000000 | -4.141819000000 |
| H | -1.058725000000 | 0.190974000000 | -4.080864000000 |
| H | 0.277191000000 | 0.760945000000 | -5.127908000000 |
| C | -0.670180000000 | 2.308123000000 | -3.902811000000 |
| H | 0.048515000000 | 3.037167000000 | -4.324124000000 |
| H | -1.650506000000 | 2.485498000000 | -4.372672000000 |
| C | 1.854809000000 | -0.289218000000 | -3.188287000000 |

| | | | |
|---|---|---|---|
| C | 1.630031000000 | -1.614422000000 | -3.621760000000 |
| C | 2.721818000000 | -2.486740000000 | -3.709452000000 |
| H | 2.546516000000 | -3.515547000000 | -4.038600000000 |
| C | 4.004395000000 | -2.081449000000 | -3.351493000000 |
| H | 4.840132000000 | -2.784955000000 | -3.399212000000 |
| C | 4.213654000000 | -0.774108000000 | -2.920358000000 |
| C | 3.158922000000 | 0.141702000000 | -2.853902000000 |
| C | -1.205532000000 | 3.665798000000 | -1.874460000000 |
| C | -2.588361000000 | 3.953363000000 | -1.891977000000 |
| C | -3.042738000000 | 5.125028000000 | -1.276329000000 |
| H | -4.115701000000 | 5.341661000000 | -1.276113000000 |
| C | -2.153951000000 | 6.009032000000 | -0.668493000000 |
| H | -2.525419000000 | 6.916901000000 | -0.184296000000 |
| C | -0.787456000000 | 5.742888000000 | -0.704149000000 |
| H | -0.079375000000 | 6.449791000000 | -0.262102000000 |
| C | -0.291754000000 | 4.586760000000 | -1.317940000000 |
| C | 3.011693000000 | -3.205993000000 | -0.152810000000 |
| H | 2.988750000000 | -4.147276000000 | -0.720718000000 |
| H | 2.299997000000 | -3.271142000000 | 0.682948000000 |
| H | 2.649953000000 | -2.415174000000 | -0.827257000000 |
| C | 6.249943000000 | -0.621606000000 | 2.805828000000 |
| H | 6.145859000000 | -0.998589000000 | 3.836955000000 |
| H | 7.281128000000 | -0.255653000000 | 2.693458000000 |
| H | 5.562766000000 | 0.224957000000 | 2.692195000000 |
| C | 4.702094000000 | 2.573061000000 | 1.427685000000 |
| H | 5.364866000000 | 3.299502000000 | 0.935607000000 |
| H | 5.187229000000 | 2.263942000000 | 2.366929000000 |
| H | 4.626522000000 | 1.681501000000 | 0.788034000000 |
| C | -0.222320000000 | 2.094891000000 | 2.554975000000 |
| H | -0.016116000000 | 1.345260000000 | 3.333034000000 |
| H | -1.073418000000 | 2.710025000000 | 2.879652000000 |
| H | -0.525415000000 | 1.536177000000 | 1.656542000000 |
| C | -2.676469000000 | -2.835949000000 | 3.994302000000 |
| H | -1.831191000000 | -3.176098000000 | 4.608995000000 |
| H | -3.576167000000 | -3.374600000000 | 4.336507000000 |
| H | -2.481911000000 | -3.130652000000 | 2.953844000000 |
| C | -4.292182000000 | 1.659133000000 | 2.203124000000 |
| H | -3.527421000000 | 2.172305000000 | 1.597393000000 |
| H | -4.898202000000 | 1.051184000000 | 1.518021000000 |
| H | -4.930001000000 | 2.433935000000 | 2.654197000000 |
| C | -2.558912000000 | -4.826864000000 | -0.574668000000 |
| H | -2.153680000000 | -5.793806000000 | -0.905800000000 |
| H | -1.807206000000 | -4.312522000000 | 0.042948000000 |
| H | -3.431579000000 | -5.011453000000 | 0.071267000000 |
| C | -4.953801000000 | -0.788268000000 | -2.539808000000 |
| H | -4.788007000000 | -0.269745000000 | -1.587397000000 |
| H | -4.758039000000 | -0.073134000000 | -3.351953000000 |
| H | -6.020780000000 | -1.064193000000 | -2.587961000000 |
| C | -3.580230000000 | 3.059141000000 | -2.595562000000 |
| H | -3.205693000000 | 2.032661000000 | -2.683923000000 |
| H | -4.536708000000 | 3.033248000000 | -2.051890000000 |
| H | -3.786467000000 | 3.439286000000 | -3.610998000000 |
| C | 1.197583000000 | 4.367745000000 | -1.401728000000 |
| H | 1.730388000000 | 5.321451000000 | -1.282062000000 |
| H | 1.554749000000 | 3.687710000000 | -0.614588000000 |
| H | 1.478007000000 | 3.928402000000 | -2.369948000000 |
| C | 3.428473000000 | 1.568038000000 | -2.453669000000 |

| | | | |
|---|---|---|---|
| H | 2.908513000000 | 2.265943000000 | -3.126986000000 |
| H | 3.070657000000 | 1.765770000000 | -1.431154000000 |
| H | 4.507064000000 | 1.780054000000 | -2.483985000000 |
| C | 0.255095000000 | -2.126714000000 | -3.977405000000 |
| H | 0.245068000000 | -3.225562000000 | -3.963512000000 |
| H | -0.503832000000 | -1.782012000000 | -3.262960000000 |
| H | -0.058158000000 | -1.803842000000 | -4.983646000000 |
| H | 5.219018000000 | -0.444694000000 | -2.641957000000 |

**TS4**

$N_{imag}$ = 0, -260.8 $cm^{-1}$
$SP_{M062X}$ = -4588.277494
$G_{M062X}$ = -4587.237572

| | | | |
|---|---|---|---|
| P | 0.180183000000 | -2.550588000000 | 0.881066000000 |
| P | 0.036341000000 | -0.746987000000 | -1.062090000000 |
| P | -1.697498000000 | 0.561659000000 | 0.738973000000 |
| P | 2.885786000000 | -0.750593000000 | 0.450587000000 |
| P | 0.388060000000 | 2.445979000000 | -0.499080000000 |
| P | -2.808111000000 | -1.385689000000 | 0.044610000000 |
| N | 3.797276000000 | -2.110829000000 | 0.975738000000 |
| N | 2.709696000000 | -0.242115000000 | 2.094812000000 |
| N | -3.784087000000 | -2.431006000000 | -0.891347000000 |
| N | -3.547327000000 | -1.925876000000 | 1.473852000000 |
| N | 1.449602000000 | 2.151274000000 | -1.823023000000 |
| N | -0.775322000000 | 3.197908000000 | -1.584950000000 |
| C | 1.223629000000 | -1.432614000000 | 0.119392000000 |
| C | -1.111798000000 | -1.704559000000 | 0.019720000000 |
| C | -0.380372000000 | 0.818697000000 | -0.252190000000 |
| C | 3.491253000000 | -2.483879000000 | 2.352486000000 |
| H | 4.332153000000 | -3.046644000000 | 2.786345000000 |
| H | 2.586139000000 | -3.115429000000 | 2.425740000000 |
| C | 3.264692000000 | -1.155943000000 | 3.086191000000 |
| H | 2.580457000000 | -1.295905000000 | 3.939938000000 |
| H | 4.221104000000 | -0.762905000000 | 3.483658000000 |
| C | 4.675700000000 | -2.846292000000 | 0.136173000000 |
| C | 4.400849000000 | -4.189727000000 | -0.193747000000 |
| C | 5.290014000000 | -4.881129000000 | -1.025252000000 |
| H | 5.072738000000 | -5.922271000000 | -1.283351000000 |
| C | 6.424546000000 | -4.258740000000 | -1.540252000000 |
| H | 7.103743000000 | -4.808680000000 | -2.198106000000 |
| C | 6.692115000000 | -2.932647000000 | -1.205601000000 |
| H | 7.591964000000 | -2.442101000000 | -1.589383000000 |
| C | 5.839540000000 | -2.216959000000 | -0.358058000000 |
| C | 2.281506000000 | 1.066604000000 | 2.454259000000 |
| C | 1.047312000000 | 1.243994000000 | 3.113485000000 |
| C | 0.622393000000 | 2.541203000000 | 3.422350000000 |
| H | -0.346358000000 | 2.678205000000 | 3.913120000000 |
| C | 1.412077000000 | 3.646467000000 | 3.112283000000 |
| H | 1.066685000000 | 4.654972000000 | 3.358576000000 |
| C | 2.647784000000 | 3.460175000000 | 2.497203000000 |
| H | 3.285128000000 | 4.321074000000 | 2.273631000000 |
| C | 3.098768000000 | 2.178930000000 | 2.162707000000 |
| C | -4.794152000000 | -3.158968000000 | -0.128309000000 |
| H | -4.920874000000 | -4.170876000000 | -0.545434000000 |

| | | | |
|---|---|---|---|
| H | -5.775694000000 | -2.651989000000 | -0.145115000000 |
| C | -4.234898000000 | -3.204775000000 | 1.294435000000 |
| H | -5.032755000000 | -3.301008000000 | 2.045012000000 |
| H | -3.543342000000 | -4.059554000000 | 1.407677000000 |
| C | -3.788746000000 | -2.407127000000 | -2.317533000000 |
| C | -2.805374000000 | -3.125186000000 | -3.025032000000 |
| C | -2.813384000000 | -3.066628000000 | -4.425119000000 |
| H | -2.051933000000 | -3.619531000000 | -4.983441000000 |
| C | -3.775297000000 | -2.327681000000 | -5.107528000000 |
| H | -3.765692000000 | -2.292817000000 | -6.200745000000 |
| C | -4.753517000000 | -1.634115000000 | -4.395648000000 |
| H | -5.508895000000 | -1.049412000000 | -4.929283000000 |
| C | -4.774393000000 | -1.662170000000 | -2.998664000000 |
| C | -3.592104000000 | -1.205198000000 | 2.700537000000 |
| C | -2.927207000000 | -1.713078000000 | 3.832479000000 |
| C | -2.976586000000 | -0.982538000000 | 5.024516000000 |
| H | -2.453037000000 | -1.366325000000 | 5.905244000000 |
| C | -3.657994000000 | 0.230719000000 | 5.091808000000 |
| H | -3.673888000000 | 0.799401000000 | 6.025933000000 |
| C | -4.329530000000 | 0.712630000000 | 3.969493000000 |
| H | -4.883440000000 | 1.654380000000 | 4.024607000000 |
| C | -4.325265000000 | -0.003700000000 | 2.768190000000 |
| C | 0.726961000000 | 2.131441000000 | -3.101316000000 |
| H | 0.274139000000 | 1.139503000000 | -3.274855000000 |
| H | 1.424349000000 | 2.338707000000 | -3.924905000000 |
| C | -0.354971000000 | 3.206167000000 | -2.983565000000 |
| H | 0.060654000000 | 4.195611000000 | -3.254833000000 |
| H | -1.195584000000 | 3.010656000000 | -3.663042000000 |
| C | 2.832418000000 | 2.497854000000 | -1.824795000000 |
| C | 3.748891000000 | 1.560869000000 | -2.349722000000 |
| C | 5.111604000000 | 1.876967000000 | -2.362906000000 |
| H | 5.820950000000 | 1.143856000000 | -2.758869000000 |
| C | 5.570561000000 | 3.097511000000 | -1.873418000000 |
| H | 6.639799000000 | 3.327756000000 | -1.880120000000 |
| C | 4.655099000000 | 4.029819000000 | -1.392619000000 |
| C | 3.281427000000 | 3.759134000000 | -1.375442000000 |
| C | -2.118773000000 | 3.366599000000 | -1.181568000000 |
| C | -3.147026000000 | 2.541926000000 | -1.694201000000 |
| C | -4.460455000000 | 2.747460000000 | -1.254518000000 |
| H | -5.256868000000 | 2.113906000000 | -1.656079000000 |
| C | -4.757955000000 | 3.711172000000 | -0.294572000000 |
| H | -5.788063000000 | 3.847918000000 | 0.046998000000 |
| C | -3.732310000000 | 4.489186000000 | 0.239103000000 |
| H | -3.956874000000 | 5.244816000000 | 0.998207000000 |
| C | -2.412354000000 | 4.336936000000 | -0.197028000000 |
| C | 3.163963000000 | -4.881060000000 | 0.321895000000 |
| H | 2.971281000000 | -5.805082000000 | -0.242175000000 |
| H | 3.262697000000 | -5.153306000000 | 1.385373000000 |
| H | 2.285232000000 | -4.228002000000 | 0.232965000000 |
| C | 6.179907000000 | -0.806180000000 | 0.044359000000 |
| H | 6.079328000000 | -0.681147000000 | 1.132731000000 |
| H | 7.209017000000 | -0.553428000000 | -0.249651000000 |
| H | 5.501933000000 | -0.076547000000 | -0.422462000000 |
| C | 4.449212000000 | 1.992231000000 | 1.522892000000 |
| H | 5.043352000000 | 2.914342000000 | 1.591300000000 |
| H | 5.003174000000 | 1.175800000000 | 2.008336000000 |
| H | 4.351998000000 | 1.735034000000 | 0.458126000000 |

| | | | |
|---|---|---|---|
| C | 0.197504000000 | 0.066429000000 | 3.513604000000 |
| H | 0.265949000000 | -0.738173000000 | 2.770582000000 |
| H | 0.527848000000 | -0.348699000000 | 4.481654000000 |
| H | -0.855742000000 | 0.357475000000 | 3.624648000000 |
| C | -2.180184000000 | -3.020110000000 | 3.769861000000 |
| H | -1.466169000000 | -3.102735000000 | 4.601754000000 |
| H | -2.870755000000 | -3.877431000000 | 3.834211000000 |
| H | -1.625652000000 | -3.115700000000 | 2.826612000000 |
| C | -5.098005000000 | 0.499183000000 | 1.577508000000 |
| H | -4.433711000000 | 0.946211000000 | 0.820789000000 |
| H | -5.648735000000 | -0.320434000000 | 1.091612000000 |
| H | -5.813963000000 | 1.276950000000 | 1.878476000000 |
| C | -1.756765000000 | -3.951107000000 | -2.321291000000 |
| H | -2.096505000000 | -4.272660000000 | -1.327859000000 |
| H | -1.500509000000 | -4.837284000000 | -2.920904000000 |
| H | -0.834626000000 | -3.366664000000 | -2.175427000000 |
| C | -5.827680000000 | -0.891146000000 | -2.241605000000 |
| H | -6.658385000000 | -1.546157000000 | -1.931819000000 |
| H | -5.411379000000 | -0.435026000000 | -1.331735000000 |
| H | -6.251371000000 | -0.094419000000 | -2.869985000000 |
| C | -2.866618000000 | 1.430617000000 | -2.674242000000 |
| H | -1.876930000000 | 0.987146000000 | -2.501800000000 |
| H | -3.613514000000 | 0.634488000000 | -2.572985000000 |
| H | -2.902552000000 | 1.778365000000 | -3.719914000000 |
| C | -1.318301000000 | 5.207998000000 | 0.366961000000 |
| H | -1.742202000000 | 6.033184000000 | 0.957559000000 |
| H | -0.640894000000 | 4.630914000000 | 1.017640000000 |
| H | -0.698619000000 | 5.625861000000 | -0.440286000000 |
| C | 2.329976000000 | 4.836211000000 | -0.912716000000 |
| H | 1.426388000000 | 4.870837000000 | -1.537841000000 |
| H | 1.997917000000 | 4.665636000000 | 0.121716000000 |
| H | 2.819559000000 | 5.819998000000 | -0.958896000000 |
| C | 3.282463000000 | 0.247702000000 | -2.924738000000 |
| H | 4.065811000000 | -0.519195000000 | -2.832832000000 |
| H | 2.379918000000 | -0.111890000000 | -2.416548000000 |
| H | 3.037545000000 | 0.353098000000 | -3.995523000000 |
| H | 5.004775000000 | 5.003883000000 | -1.036749000000 |

**INT3**

$N_{imag}$ **=** 0
$SP_{M062X}$ **=** -4588.324886
$G_{M062X}$ **=** -4587.284928

| | | | |
|---|---|---|---|
| P | -0.448437000000 | -1.634008000000 | 1.562923000000 |
| P | -0.047340000000 | -0.441284000000 | -0.754133000000 |
| P | -1.558089000000 | 1.191651000000 | 0.658464000000 |
| P | 2.685406000000 | -0.904699000000 | 0.812382000000 |
| P | 0.824154000000 | 2.731888000000 | -0.790602000000 |
| P | -3.097388000000 | -1.269394000000 | -0.351175000000 |
| N | 3.235726000000 | -2.277463000000 | 1.681251000000 |
| N | 2.750936000000 | 0.038031000000 | 2.256349000000 |
| N | -2.937334000000 | -2.984975000000 | -0.315642000000 |
| N | -4.139278000000 | -1.308978000000 | 1.026446000000 |
| N | 1.719543000000 | 2.111621000000 | -2.116180000000 |
| N | -0.333715000000 | 3.476953000000 | -1.887473000000 |

| | | | |
|---|---|---|---|
| C | 0.873528000000 | -1.133019000000 | 0.649944000000 |
| C | -1.513160000000 | -0.682377000000 | 0.386263000000 |
| C | -0.182578000000 | 1.306091000000 | -0.296334000000 |
| C | 2.957269000000 | -2.174193000000 | 3.110870000000 |
| H | 3.640142000000 | -2.825715000000 | 3.677076000000 |
| H | 1.920161000000 | -2.463259000000 | 3.360443000000 |
| C | 3.175211000000 | -0.696994000000 | 3.443560000000 |
| H | 2.586792000000 | -0.403146000000 | 4.328657000000 |
| H | 4.241969000000 | -0.498134000000 | 3.665444000000 |
| C | 3.716502000000 | -3.449288000000 | 1.038830000000 |
| C | 3.036239000000 | -4.678626000000 | 1.170154000000 |
| C | 3.538410000000 | -5.805671000000 | 0.508960000000 |
| H | 3.007087000000 | -6.757208000000 | 0.609209000000 |
| C | 4.681134000000 | -5.726476000000 | -0.283240000000 |
| H | 5.054598000000 | -6.613886000000 | -0.802131000000 |
| C | 5.348114000000 | -4.509397000000 | -0.404673000000 |
| H | 6.256953000000 | -4.442493000000 | -1.010555000000 |
| C | 4.891029000000 | -3.364662000000 | 0.257300000000 |
| C | 2.715532000000 | 1.462062000000 | 2.264881000000 |
| C | 1.639154000000 | 2.125067000000 | 2.889158000000 |
| C | 1.614815000000 | 3.524366000000 | 2.890784000000 |
| H | 0.772394000000 | 4.040760000000 | 3.361625000000 |
| C | 2.640760000000 | 4.258572000000 | 2.301393000000 |
| H | 2.606577000000 | 5.352063000000 | 2.307027000000 |
| C | 3.712355000000 | 3.595318000000 | 1.707575000000 |
| H | 4.529667000000 | 4.165484000000 | 1.255970000000 |
| C | 3.769528000000 | 2.198284000000 | 1.684144000000 |
| C | -3.640481000000 | -3.621106000000 | 0.802210000000 |
| H | -3.029307000000 | -4.422307000000 | 1.252109000000 |
| H | -4.584657000000 | -4.077424000000 | 0.450883000000 |
| C | -3.917216000000 | -2.514351000000 | 1.822602000000 |
| H | -4.809881000000 | -2.737440000000 | 2.427766000000 |
| H | -3.056294000000 | -2.428977000000 | 2.506899000000 |
| C | -2.788268000000 | -3.747397000000 | -1.520042000000 |
| C | -1.604446000000 | -4.487833000000 | -1.710218000000 |
| C | -1.463177000000 | -5.257440000000 | -2.870206000000 |
| H | -0.543972000000 | -5.832190000000 | -3.018235000000 |
| C | -2.472281000000 | -5.295669000000 | -3.830217000000 |
| H | -2.348765000000 | -5.900478000000 | -4.733288000000 |
| C | -3.641698000000 | -4.566011000000 | -3.631862000000 |
| H | -4.440949000000 | -4.598850000000 | -4.378639000000 |
| C | -3.819771000000 | -3.790632000000 | -2.479730000000 |
| C | -4.727029000000 | -0.115704000000 | 1.540488000000 |
| C | -4.300347000000 | 0.473025000000 | 2.751757000000 |
| C | -4.910818000000 | 1.657839000000 | 3.181980000000 |
| H | -4.571247000000 | 2.113327000000 | 4.117039000000 |
| C | -5.909616000000 | 2.273280000000 | 2.434949000000 |
| H | -6.364072000000 | 3.205351000000 | 2.782287000000 |
| C | -6.321310000000 | 1.690848000000 | 1.239477000000 |
| H | -7.110841000000 | 2.160223000000 | 0.644694000000 |
| C | -5.752585000000 | 0.497875000000 | 0.784297000000 |
| C | 0.914703000000 | 2.039358000000 | -3.340501000000 |
| H | 0.329208000000 | 1.103292000000 | -3.369351000000 |
| H | 1.574146000000 | 2.057895000000 | -4.219860000000 |
| C | -0.011447000000 | 3.258802000000 | -3.295971000000 |
| H | 0.510427000000 | 4.141957000000 | -3.710440000000 |
| H | -0.915932000000 | 3.104693000000 | -3.898361000000 |

| | | | |
|---|---|---|---|
| C | 3.141119000000 | 2.164517000000 | -2.198468000000 |
| C | 3.833786000000 | 0.993757000000 | -2.574772000000 |
| C | 5.230303000000 | 1.027041000000 | -2.655660000000 |
| H | 5.767126000000 | 0.115893000000 | -2.936870000000 |
| C | 5.936063000000 | 2.194019000000 | -2.372331000000 |
| H | 7.028291000000 | 2.204683000000 | -2.428388000000 |
| C | 5.240921000000 | 3.351883000000 | -2.031765000000 |
| C | 3.842706000000 | 3.365414000000 | -1.957947000000 |
| C | -1.623058000000 | 3.798967000000 | -1.399337000000 |
| C | -2.766680000000 | 3.038666000000 | -1.742739000000 |
| C | -3.991769000000 | 3.353150000000 | -1.142174000000 |
| H | -4.870296000000 | 2.750191000000 | -1.390232000000 |
| C | -4.096668000000 | 4.372853000000 | -0.201239000000 |
| H | -5.057626000000 | 4.581856000000 | 0.276267000000 |
| C | -2.964947000000 | 5.109869000000 | 0.140011000000 |
| H | -3.038600000000 | 5.916452000000 | 0.875995000000 |
| C | -1.726249000000 | 4.843423000000 | -0.452086000000 |
| C | 1.779175000000 | -4.799060000000 | 1.995612000000 |
| H | 1.238780000000 | -5.722120000000 | 1.742449000000 |
| H | 2.002100000000 | -4.827939000000 | 3.074391000000 |
| H | 1.108153000000 | -3.947993000000 | 1.820269000000 |
| C | 5.664208000000 | -2.074676000000 | 0.147623000000 |
| H | 5.735402000000 | -1.579971000000 | 1.127383000000 |
| H | 6.679178000000 | -2.262802000000 | -0.231458000000 |
| H | 5.171742000000 | -1.360394000000 | -0.531358000000 |
| C | 4.952007000000 | 1.497360000000 | 1.068425000000 |
| H | 5.785949000000 | 2.197818000000 | 0.922409000000 |
| H | 5.293495000000 | 0.666557000000 | 1.703266000000 |
| H | 4.695321000000 | 1.075377000000 | 0.085584000000 |
| C | 0.541747000000 | 1.358180000000 | 3.582659000000 |
| H | 0.416073000000 | 0.363800000000 | 3.136658000000 |
| H | 0.776343000000 | 1.217876000000 | 4.652008000000 |
| H | -0.414145000000 | 1.898572000000 | 3.520316000000 |
| C | -3.184873000000 | -0.098791000000 | 3.592698000000 |
| H | -2.843739000000 | 0.645382000000 | 4.325921000000 |
| H | -3.501748000000 | -0.995096000000 | 4.148591000000 |
| H | -2.318146000000 | -0.384795000000 | 2.981259000000 |
| C | -6.251055000000 | -0.120706000000 | -0.495290000000 |
| H | -5.497355000000 | -0.051862000000 | -1.295310000000 |
| H | -6.468823000000 | -1.188979000000 | -0.353366000000 |
| H | -7.163924000000 | 0.386161000000 | -0.839599000000 |
| C | -0.488959000000 | -4.437160000000 | -0.699344000000 |
| H | 0.230202000000 | -5.252871000000 | -0.863650000000 |
| H | 0.062208000000 | -3.487216000000 | -0.775039000000 |
| H | -0.866543000000 | -4.501101000000 | 0.330386000000 |
| C | -5.092067000000 | -3.001467000000 | -2.302407000000 |
| H | -5.417480000000 | -2.978893000000 | -1.254021000000 |
| H | -4.947276000000 | -1.955567000000 | -2.615702000000 |
| H | -5.902539000000 | -3.427521000000 | -2.911433000000 |
| C | -2.708556000000 | 1.867473000000 | -2.692601000000 |
| H | -1.765973000000 | 1.313296000000 | -2.588511000000 |
| H | -3.528390000000 | 1.167416000000 | -2.479832000000 |
| H | -2.802252000000 | 2.183679000000 | -3.744770000000 |
| C | -0.515728000000 | 5.663040000000 | -0.077039000000 |
| H | -0.818234000000 | 6.603018000000 | 0.407453000000 |
| H | 0.141254000000 | 5.117427000000 | 0.620698000000 |
| H | 0.090582000000 | 5.893572000000 | -0.965480000000 |

| | | | |
|---|---|---|---|
| C | 3.128589000000 | 4.663917000000 | -1.668977000000 |
| H | 2.230657000000 | 4.773880000000 | -2.294403000000 |
| H | 2.800773000000 | 4.720358000000 | -0.620539000000 |
| H | 3.794210000000 | 5.516770000000 | -1.866567000000 |
| C | 3.089251000000 | -0.272699000000 | -2.910057000000 |
| H | 3.745756000000 | -1.150191000000 | -2.819078000000 |
| H | 2.227424000000 | -0.412236000000 | -2.245764000000 |
| H | 2.702077000000 | -0.245294000000 | -3.942395000000 |
| H | 5.788609000000 | 4.279663000000 | -1.839449000000 |

**TS5**

$\boldsymbol{N_{imag}}$ **=** 0, 397.9 $cm^{-1}$
$\boldsymbol{SP_{M062X}}$ **=** -4588.27897533
$\boldsymbol{G_{M062X}}$ **=** -4587.241676

| | | | |
|---|---|---|---|
| P | 1.094469000000 | 1.341985000000 | 0.025603000000 |
| P | -0.693989000000 | -0.308059000000 | -1.022239000000 |
| P | 0.703318000000 | -1.593249000000 | 1.011311000000 |
| P | -1.728706000000 | 2.694769000000 | -0.435918000000 |
| P | -2.212290000000 | -2.558668000000 | 0.644890000000 |
| P | 3.412462000000 | -0.630295000000 | 0.267332000000 |
| N | -0.744579000000 | 4.069834000000 | -0.157455000000 |
| N | -2.271637000000 | 2.735802000000 | 1.209781000000 |
| N | 3.940278000000 | -0.322329000000 | -1.359889000000 |
| N | 4.126163000000 | 0.856375000000 | 0.773259000000 |
| N | -3.292000000000 | -2.216483000000 | -0.654144000000 |
| N | -1.632861000000 | -3.980633000000 | -0.197325000000 |
| C | -0.556385000000 | 1.288763000000 | -0.359882000000 |
| C | 1.618098000000 | -0.328384000000 | 0.171040000000 |
| C | -0.828548000000 | -1.402525000000 | 0.332138000000 |
| C | -0.468688000000 | 4.295840000000 | 1.266316000000 |
| H | -0.275190000000 | 5.364481000000 | 1.438531000000 |
| H | 0.420075000000 | 3.730179000000 | 1.593646000000 |
| C | -1.716593000000 | 3.818647000000 | 2.014260000000 |
| H | -1.462563000000 | 3.474007000000 | 3.027975000000 |
| H | -2.447501000000 | 4.644140000000 | 2.118753000000 |
| C | -0.602007000000 | 5.131460000000 | -1.098170000000 |
| C | 0.697723000000 | 5.567689000000 | -1.433173000000 |
| C | 0.850446000000 | 6.607618000000 | -2.355637000000 |
| H | 1.859286000000 | 6.939155000000 | -2.620006000000 |
| C | -0.259116000000 | 7.211881000000 | -2.943323000000 |
| H | -0.126790000000 | 8.018932000000 | -3.669610000000 |
| C | -1.538299000000 | 6.792923000000 | -2.587193000000 |
| H | -2.413948000000 | 7.283683000000 | -3.023106000000 |
| C | -1.733025000000 | 5.766202000000 | -1.654665000000 |
| C | -3.048113000000 | 1.689217000000 | 1.777585000000 |
| C | -2.470922000000 | 0.773034000000 | 2.683329000000 |
| C | -3.269117000000 | -0.255507000000 | 3.198985000000 |
| H | -2.820290000000 | -0.978991000000 | 3.886010000000 |
| C | -4.606375000000 | -0.380468000000 | 2.834778000000 |
| H | -5.212283000000 | -1.197947000000 | 3.235513000000 |
| C | -5.169595000000 | 0.533904000000 | 1.948279000000 |
| H | -6.217425000000 | 0.435623000000 | 1.655803000000 |
| C | -4.406712000000 | 1.575267000000 | 1.413861000000 |
| C | 4.796586000000 | 0.840230000000 | -1.535338000000 |
| H | 4.559387000000 | 1.368806000000 | -2.473282000000 |

| | | | |
|---|---|---|---|
| H | 5.864476000000 | 0.548343000000 | -1.586174000000 |
| C | 4.531013000000 | 1.740613000000 | -0.323027000000 |
| H | 5.433501000000 | 2.300524000000 | -0.033854000000 |
| H | 3.738429000000 | 2.472488000000 | -0.558633000000 |
| C | 3.701043000000 | -1.218339000000 | -2.434502000000 |
| C | 2.829590000000 | -0.839442000000 | -3.477861000000 |
| C | 2.602547000000 | -1.733264000000 | -4.532025000000 |
| H | 1.920470000000 | -1.442355000000 | -5.336986000000 |
| C | 3.218956000000 | -2.981314000000 | -4.559519000000 |
| H | 3.026705000000 | -3.671586000000 | -5.385977000000 |
| C | 4.081180000000 | -3.348309000000 | -3.527207000000 |
| H | 4.574407000000 | -4.324958000000 | -3.547399000000 |
| C | 4.339217000000 | -2.478023000000 | -2.462922000000 |
| C | 4.727149000000 | 0.998545000000 | 2.062922000000 |
| C | 4.157255000000 | 1.887215000000 | 2.995466000000 |
| C | 4.760830000000 | 2.039753000000 | 4.248772000000 |
| H | 4.318529000000 | 2.731033000000 | 4.972557000000 |
| C | 5.906714000000 | 1.320577000000 | 4.582029000000 |
| H | 6.366996000000 | 1.445339000000 | 5.566449000000 |
| C | 6.467224000000 | 0.446609000000 | 3.653429000000 |
| H | 7.372723000000 | -0.113225000000 | 3.906500000000 |
| C | 5.895807000000 | 0.279650000000 | 2.386474000000 |
| C | -2.891888000000 | -2.889027000000 | -1.898366000000 |
| H | -2.145829000000 | -2.288635000000 | -2.450519000000 |
| H | -3.770980000000 | -3.015734000000 | -2.546049000000 |
| C | -2.290966000000 | -4.229152000000 | -1.476923000000 |
| H | -3.088266000000 | -4.987686000000 | -1.361172000000 |
| H | -1.583635000000 | -4.610903000000 | -2.226014000000 |
| C | -4.664961000000 | -1.864493000000 | -0.493167000000 |
| C | -5.137990000000 | -0.753942000000 | -1.220841000000 |
| C | -6.491815000000 | -0.417087000000 | -1.150047000000 |
| H | -6.857921000000 | 0.444021000000 | -1.717104000000 |
| C | -7.366572000000 | -1.154615000000 | -0.353488000000 |
| H | -8.424518000000 | -0.882242000000 | -0.299428000000 |
| C | -6.886221000000 | -2.240659000000 | 0.373571000000 |
| C | -5.539464000000 | -2.622241000000 | 0.308778000000 |
| C | -0.384003000000 | -4.547633000000 | 0.155299000000 |
| C | 0.758890000000 | -4.363482000000 | -0.655746000000 |
| C | 1.986589000000 | -4.878286000000 | -0.219007000000 |
| H | 2.870981000000 | -4.727856000000 | -0.845255000000 |
| C | 2.098013000000 | -5.536462000000 | 1.001699000000 |
| H | 3.067832000000 | -5.917106000000 | 1.334561000000 |
| C | 0.967991000000 | -5.701286000000 | 1.802038000000 |
| H | 1.048743000000 | -6.220993000000 | 2.761647000000 |
| C | -0.278811000000 | -5.220684000000 | 1.392667000000 |
| C | 1.908571000000 | 4.931713000000 | -0.800124000000 |
| H | 2.820721000000 | 5.175589000000 | -1.363833000000 |
| H | 2.051579000000 | 5.283784000000 | 0.235205000000 |
| H | 1.796530000000 | 3.840259000000 | -0.750643000000 |
| C | -3.139036000000 | 5.392417000000 | -1.251976000000 |
| H | -3.196276000000 | 5.138860000000 | -0.183735000000 |
| H | -3.827465000000 | 6.227255000000 | -1.448381000000 |
| H | -3.500847000000 | 4.514625000000 | -1.809394000000 |
| C | -5.036659000000 | 2.581913000000 | 0.483873000000 |
| H | -6.118052000000 | 2.404347000000 | 0.397343000000 |
| H | -4.875311000000 | 3.607437000000 | 0.850616000000 |
| H | -4.599420000000 | 2.532849000000 | -0.524899000000 |

| | | | |
|---|---|---|---|
| C | -1.035864000000 | 0.884769000000 | 3.133192000000 |
| H | -0.407330000000 | 1.327298000000 | 2.351249000000 |
| H | -0.949511000000 | 1.513659000000 | 4.035739000000 |
| H | -0.632872000000 | -0.107399000000 | 3.382040000000 |
| C | 2.899438000000 | 2.649003000000 | 2.664079000000 |
| H | 2.679252000000 | 3.397996000000 | 3.438850000000 |
| H | 2.973314000000 | 3.159002000000 | 1.692969000000 |
| H | 2.040543000000 | 1.962048000000 | 2.600189000000 |
| C | 6.529568000000 | -0.657608000000 | 1.390595000000 |
| H | 5.899384000000 | -1.546346000000 | 1.234271000000 |
| H | 6.657386000000 | -0.175326000000 | 0.410375000000 |
| H | 7.515329000000 | -0.993179000000 | 1.743326000000 |
| C | 2.154637000000 | 0.508395000000 | -3.481742000000 |
| H | 1.179769000000 | 0.458312000000 | -3.988589000000 |
| H | 2.002247000000 | 0.871931000000 | -2.457860000000 |
| H | 2.766398000000 | 1.258185000000 | -4.011520000000 |
| C | 5.298285000000 | -2.879305000000 | -1.371208000000 |
| H | 6.028201000000 | -2.079650000000 | -1.177925000000 |
| H | 4.769877000000 | -3.064777000000 | -0.422682000000 |
| H | 5.842362000000 | -3.793791000000 | -1.648452000000 |
| C | 0.715939000000 | -3.603574000000 | -1.956558000000 |
| H | -0.054839000000 | -2.824220000000 | -1.942592000000 |
| H | 1.681782000000 | -3.120287000000 | -2.148757000000 |
| H | 0.507246000000 | -4.267285000000 | -2.812135000000 |
| C | -1.495811000000 | -5.417496000000 | 2.262143000000 |
| H | -1.299697000000 | -6.166055000000 | 3.043528000000 |
| H | -1.791015000000 | -4.477421000000 | 2.756703000000 |
| H | -2.358479000000 | -5.742474000000 | 1.661461000000 |
| C | -5.070745000000 | -3.826789000000 | 1.087449000000 |
| H | -4.353509000000 | -4.430025000000 | 0.512263000000 |
| H | -4.561838000000 | -3.525379000000 | 2.016665000000 |
| H | -5.923165000000 | -4.466823000000 | 1.357320000000 |
| C | -4.182505000000 | 0.074427000000 | -2.039631000000 |
| H | -4.692731000000 | 0.937196000000 | -2.490317000000 |
| H | -3.372777000000 | 0.462656000000 | -1.400339000000 |
| H | -3.711445000000 | -0.503802000000 | -2.848956000000 |
| H | -7.571548000000 | -2.826962000000 | 0.993457000000 |

**1M**

$N_{imag}$ **=** 0
$SP_{M062X}$ **=** -4588.333203
$G_{M062X}$ **=** -4587.296524

| | | | |
|---|---|---|---|
| P | 0.454817000000 | 1.674721000000 | 0.449348000000 |
| P | 0.910428000000 | -1.429419000000 | 0.643984000000 |
| P | -1.843790000000 | -0.305709000000 | -0.373638000000 |
| P | 3.113620000000 | 0.353322000000 | 1.336024000000 |
| P | -1.450187000000 | -3.187461000000 | -0.241539000000 |
| P | -2.051562000000 | 2.768421000000 | -0.574805000000 |
| N | 3.127909000000 | 1.861308000000 | 2.162748000000 |
| N | 3.888780000000 | 1.068061000000 | -0.029581000000 |
| N | -0.997719000000 | -4.128822000000 | 1.113647000000 |
| N | -0.183821000000 | -3.929027000000 | -1.178907000000 |
| N | -3.665147000000 | 2.310304000000 | -0.181562000000 |
| N | -2.380324000000 | 2.551078000000 | -2.251804000000 |
| C | 1.352565000000 | 0.213160000000 | 0.767160000000 |

| | | | |
|---|---|---|---|
| C | -0.709748000000 | -1.512182000000 | 0.023997000000 |
| C | -1.088196000000 | 1.244632000000 | -0.134837000000 |
| C | 3.561982000000 | 3.000620000000 | 1.349663000000 |
| H | 4.540073000000 | 3.369107000000 | 1.702235000000 |
| H | 2.841823000000 | 3.833754000000 | 1.423546000000 |
| C | 3.659263000000 | 2.508830000000 | -0.104029000000 |
| H | 2.731873000000 | 2.766900000000 | -0.642406000000 |
| H | 4.496194000000 | 2.989457000000 | -0.635041000000 |
| C | 3.315841000000 | 1.914548000000 | 3.582163000000 |
| C | 2.185002000000 | 2.021299000000 | 4.418346000000 |
| C | 2.365936000000 | 2.082450000000 | 5.803693000000 |
| H | 1.488335000000 | 2.167162000000 | 6.451621000000 |
| C | 3.642871000000 | 2.040026000000 | 6.361403000000 |
| H | 3.770398000000 | 2.089205000000 | 7.446730000000 |
| C | 4.753547000000 | 1.928352000000 | 5.530796000000 |
| H | 5.758029000000 | 1.881910000000 | 5.963076000000 |
| C | 4.609712000000 | 1.861280000000 | 4.138988000000 |
| C | 4.381305000000 | 0.319777000000 | -1.139405000000 |
| C | 5.537230000000 | -0.475489000000 | -0.960077000000 |
| C | 6.055103000000 | -1.186778000000 | -2.046727000000 |
| H | 6.947873000000 | -1.801681000000 | -1.898826000000 |
| C | 5.473541000000 | -1.095032000000 | -3.309636000000 |
| H | 5.900368000000 | -1.640814000000 | -4.155927000000 |
| C | 4.337891000000 | -0.310847000000 | -3.479368000000 |
| H | 3.859244000000 | -0.246688000000 | -4.460925000000 |
| C | 3.759366000000 | 0.380060000000 | -2.405755000000 |
| C | 0.215197000000 | -4.928216000000 | 0.936769000000 |
| H | 0.115289000000 | -5.880814000000 | 1.479721000000 |
| H | 1.105160000000 | -4.401316000000 | 1.326475000000 |
| C | 0.342124000000 | -5.147544000000 | -0.572221000000 |
| H | -0.236579000000 | -6.036794000000 | -0.889486000000 |
| H | 1.388398000000 | -5.311140000000 | -0.867431000000 |
| C | -1.935734000000 | -4.406441000000 | 2.152958000000 |
| C | -1.894906000000 | -3.653882000000 | 3.341078000000 |
| C | -2.823892000000 | -3.936435000000 | 4.349946000000 |
| H | -2.795768000000 | -3.358465000000 | 5.278689000000 |
| C | -3.777300000000 | -4.938683000000 | 4.181475000000 |
| H | -4.497975000000 | -5.147955000000 | 4.977418000000 |
| C | -3.813561000000 | -5.673666000000 | 2.997073000000 |
| H | -4.565139000000 | -6.457364000000 | 2.861569000000 |
| C | -2.897210000000 | -5.419882000000 | 1.971080000000 |
| C | 0.113747000000 | -3.559072000000 | -2.517305000000 |
| C | 1.437337000000 | -3.200666000000 | -2.865286000000 |
| C | 1.709354000000 | -2.799772000000 | -4.178820000000 |
| H | 2.734000000000 | -2.520587000000 | -4.438735000000 |
| C | 0.704104000000 | -2.742463000000 | -5.140286000000 |
| H | 0.932781000000 | -2.418622000000 | -6.159831000000 |
| C | -0.592557000000 | -3.115794000000 | -4.796174000000 |
| H | -1.384669000000 | -3.099024000000 | -5.551091000000 |
| C | -0.903843000000 | -3.540902000000 | -3.499695000000 |
| C | -4.500391000000 | 1.960280000000 | -1.331351000000 |
| H | -5.202990000000 | 2.782489000000 | -1.562160000000 |
| H | -5.100622000000 | 1.058156000000 | -1.121700000000 |
| C | -3.547595000000 | 1.710177000000 | -2.506265000000 |
| H | -3.299854000000 | 0.637247000000 | -2.558189000000 |
| H | -4.008512000000 | 1.996697000000 | -3.464857000000 |
| C | -4.312362000000 | 2.799570000000 | 0.998990000000 |

| | | | |
|---|---|---|---|
| C | -4.655838000000 | 4.162369000000 | 1.110907000000 |
| C | -5.308244000000 | 4.605686000000 | 2.267209000000 |
| H | -5.573989000000 | 5.663513000000 | 2.355430000000 |
| C | -5.624501000000 | 3.719203000000 | 3.293907000000 |
| H | -6.138507000000 | 4.077965000000 | 4.190376000000 |
| C | -5.286250000000 | 2.373165000000 | 3.172996000000 |
| C | -4.628005000000 | 1.896278000000 | 2.033420000000 |
| C | -1.453312000000 | 2.914623000000 | -3.270997000000 |
| C | -1.190937000000 | 4.287099000000 | -3.486502000000 |
| C | -0.279111000000 | 4.662516000000 | -4.478044000000 |
| H | -0.079431000000 | 5.726423000000 | -4.637817000000 |
| C | 0.349819000000 | 3.708033000000 | -5.274176000000 |
| H | 1.052433000000 | 4.014999000000 | -6.054127000000 |
| C | 0.072594000000 | 2.360069000000 | -5.069609000000 |
| H | 0.565886000000 | 1.601803000000 | -5.685441000000 |
| C | -0.811289000000 | 1.940082000000 | -4.067022000000 |
| H | -5.533805000000 | 1.672415000000 | 3.976052000000 |
| C | -4.245396000000 | 0.440274000000 | 1.942219000000 |
| H | -4.490351000000 | 0.011365000000 | 0.960847000000 |
| H | -3.159483000000 | 0.316141000000 | 2.085022000000 |
| H | -4.753254000000 | -0.147377000000 | 2.720703000000 |
| C | -4.307712000000 | 5.144984000000 | 0.021898000000 |
| H | -4.849097000000 | 6.091332000000 | 0.163319000000 |
| H | -3.228894000000 | 5.364988000000 | 0.026983000000 |
| H | -4.549873000000 | 4.752411000000 | -0.975456000000 |
| C | -1.896318000000 | 5.347683000000 | -2.682102000000 |
| H | -1.520054000000 | 5.382546000000 | -1.647675000000 |
| H | -1.750276000000 | 6.339121000000 | -3.134271000000 |
| H | -2.973748000000 | 5.137678000000 | -2.623602000000 |
| C | -1.022811000000 | 0.459946000000 | -3.866735000000 |
| H | -0.240249000000 | -0.118299000000 | -4.376087000000 |
| H | -0.991807000000 | 0.193207000000 | -2.802400000000 |
| H | -1.992601000000 | 0.120849000000 | -4.264104000000 |
| C | -0.886611000000 | -2.545342000000 | 3.518022000000 |
| H | 0.114450000000 | -2.850803000000 | 3.180190000000 |
| H | -1.170944000000 | -1.662593000000 | 2.922379000000 |
| H | -0.824891000000 | -2.236108000000 | 4.571518000000 |
| C | -2.942055000000 | -6.206087000000 | 0.684632000000 |
| H | -3.069695000000 | -5.536473000000 | -0.181023000000 |
| H | -2.010512000000 | -6.771359000000 | 0.525615000000 |
| H | -3.776706000000 | -6.921454000000 | 0.692937000000 |
| C | -2.309401000000 | -4.003838000000 | -3.198037000000 |
| H | -2.823975000000 | -4.284071000000 | -4.128870000000 |
| H | -2.305691000000 | -4.873231000000 | -2.523727000000 |
| H | -2.903874000000 | -3.219658000000 | -2.702959000000 |
| C | 2.474673000000 | 1.131243000000 | -2.651679000000 |
| H | 2.632166000000 | 2.216038000000 | -2.760902000000 |
| H | 2.003151000000 | 0.774893000000 | -3.576471000000 |
| H | 1.760675000000 | 0.977359000000 | -1.832684000000 |
| C | 6.250064000000 | -0.527792000000 | 0.366625000000 |
| H | 7.111446000000 | -1.208902000000 | 0.315259000000 |
| H | 6.618944000000 | 0.472027000000 | 0.642090000000 |
| H | 5.588490000000 | -0.866158000000 | 1.178093000000 |
| C | 2.578146000000 | -3.262385000000 | -1.879047000000 |
| H | 3.116502000000 | -4.221895000000 | -1.965430000000 |
| H | 3.304354000000 | -2.461041000000 | -2.075172000000 |
| H | 2.228984000000 | -3.168275000000 | -0.844155000000 |

| | | | |
|---|---|---|---|
| C | 5.841969000000 | 1.729867000000 | 3.279034000000 |
| H | 5.598991000000 | 1.348456000000 | 2.281891000000 |
| H | 6.341832000000 | 2.705061000000 | 3.153795000000 |
| H | 6.568127000000 | 1.046699000000 | 3.744720000000 |
| C | 0.793551000000 | 2.060476000000 | 3.838980000000 |
| H | 0.514914000000 | 1.084075000000 | 3.411488000000 |
| H | 0.055302000000 | 2.314359000000 | 4.613358000000 |
| H | 0.721097000000 | 2.798790000000 | 3.027080000000 |

**ORCA(Geometries original systems at(gas-phase)-ωB97x-D3BJ/def2-SVP)**

**1**

| | | | |
|---|---|---|---|
| P | 26.783511000000 | 3.179471000000 | 22.110748000000 |
| P | 25.654129000000 | 3.353174000000 | 19.184017000000 |
| P | 27.641866000000 | 5.545936000000 | 20.234720000000 |
| P | 24.927127000000 | 1.078920000000 | 20.824787000000 |
| P | 26.620985000000 | 5.785411000000 | 17.532286000000 |
| P | 28.786060000000 | 5.048401000000 | 23.066804000000 |
| N | 24.024481000000 | 1.205457000000 | 22.299801000000 |
| N | 26.001765000000 | -0.022481000000 | 21.594521000000 |
| N | 24.978502000000 | 6.020934000000 | 17.079673000000 |
| N | 26.773240000000 | 4.564182000000 | 16.310399000000 |
| N | 28.380496000000 | 6.708965000000 | 23.364024000000 |
| N | 30.255686000000 | 5.553146000000 | 22.333253000000 |
| C | 25.830078000000 | 2.689992000000 | 20.749028000000 |
| C | 26.598918000000 | 4.803880000000 | 19.100370000000 |
| C | 27.647047000000 | 4.590200000000 | 21.680901000000 |
| C | 24.327602000000 | 0.151923000000 | 23.259748000000 |
| H | 23.691246000000 | -0.741974000000 | 23.095833000000 |
| H | 24.146162000000 | 0.506947000000 | 24.287512000000 |
| C | 25.800582000000 | -0.179817000000 | 23.032000000000 |
| H | 26.438607000000 | 0.503223000000 | 23.622921000000 |
| H | 26.036639000000 | -1.213727000000 | 23.328523000000 |
| C | 22.775858000000 | 1.889394000000 | 22.387712000000 |
| C | 21.666699000000 | 1.497656000000 | 21.605564000000 |
| C | 20.472530000000 | 2.218768000000 | 21.723292000000 |
| H | 19.612936000000 | 1.934807000000 | 21.109888000000 |
| C | 20.354809000000 | 3.278591000000 | 22.616249000000 |
| H | 19.410894000000 | 3.824858000000 | 22.699103000000 |
| C | 21.445088000000 | 3.640290000000 | 23.401819000000 |
| H | 21.353462000000 | 4.476623000000 | 24.100561000000 |
| C | 22.664530000000 | 2.965758000000 | 23.297325000000 |
| C | 21.719478000000 | 0.325476000000 | 20.635487000000 |
| H | 22.688799000000 | -0.174493000000 | 20.761075000000 |
| C | 20.635895000000 | -0.717044000000 | 20.938308000000 |
| H | 20.757139000000 | -1.594973000000 | 20.282794000000 |
| H | 20.693815000000 | -1.058164000000 | 21.983916000000 |
| H | 19.623504000000 | -0.315746000000 | 20.771068000000 |
| C | 21.640500000000 | 0.815959000000 | 19.184382000000 |
| H | 21.704420000000 | -0.028415000000 | 18.480313000000 |
| H | 20.689707000000 | 1.341658000000 | 18.997092000000 |
| H | 22.467868000000 | 1.507136000000 | 18.960266000000 |
| C | 23.840106000000 | 3.419667000000 | 24.148557000000 |
| H | 24.686317000000 | 2.760769000000 | 23.910634000000 |
| C | 23.547357000000 | 3.288100000000 | 25.648204000000 |
| H | 24.439635000000 | 3.556169000000 | 26.236454000000 |

| | | | |
|---|---|---|---|
| H | 22.729680000000 | 3.958894000000 | 25.958927000000 |
| H | 23.254215000000 | 2.259771000000 | 25.913948000000 |
| C | 24.259622000000 | 4.849451000000 | 23.792545000000 |
| H | 25.151391000000 | 5.153154000000 | 24.360571000000 |
| H | 24.493367000000 | 4.935729000000 | 22.722246000000 |
| H | 23.462555000000 | 5.575965000000 | 24.016973000000 |
| C | 26.917980000000 | -0.819845000000 | 20.849049000000 |
| C | 26.428794000000 | -1.779181000000 | 19.933024000000 |
| C | 27.345144000000 | -2.491626000000 | 19.152093000000 |
| H | 26.976249000000 | -3.221604000000 | 18.426020000000 |
| C | 28.716107000000 | -2.304518000000 | 19.297593000000 |
| H | 29.417324000000 | -2.876103000000 | 18.682887000000 |
| C | 29.188755000000 | -1.394027000000 | 20.237601000000 |
| H | 30.266343000000 | -1.254532000000 | 20.360509000000 |
| C | 28.308914000000 | -0.634383000000 | 21.014137000000 |
| C | 24.944786000000 | -2.081333000000 | 19.781844000000 |
| H | 24.411031000000 | -1.490643000000 | 20.538740000000 |
| C | 24.642762000000 | -3.557537000000 | 20.072144000000 |
| H | 23.555488000000 | -3.737769000000 | 20.050209000000 |
| H | 25.105206000000 | -4.222481000000 | 19.325013000000 |
| H | 25.020552000000 | -3.852891000000 | 21.063645000000 |
| C | 24.421272000000 | -1.662341000000 | 18.403157000000 |
| H | 23.343433000000 | -1.875204000000 | 18.315430000000 |
| H | 24.570953000000 | -0.584268000000 | 18.241097000000 |
| H | 24.938461000000 | -2.211542000000 | 17.599169000000 |
| C | 28.873334000000 | 0.353001000000 | 22.023940000000 |
| H | 28.029264000000 | 0.931697000000 | 22.417964000000 |
| C | 29.525810000000 | -0.375493000000 | 23.205998000000 |
| H | 29.879627000000 | 0.348997000000 | 23.957397000000 |
| H | 28.817571000000 | -1.063475000000 | 23.694337000000 |
| H | 30.393981000000 | -0.968699000000 | 22.874507000000 |
| C | 29.840080000000 | 1.356997000000 | 21.390838000000 |
| H | 30.182883000000 | 2.089211000000 | 22.137994000000 |
| H | 30.737185000000 | 0.870568000000 | 20.976956000000 |
| H | 29.351646000000 | 1.911152000000 | 20.577154000000 |
| C | 24.442195000000 | 4.998963000000 | 16.184335000000 |
| H | 23.674468000000 | 5.442932000000 | 15.530929000000 |
| H | 23.982368000000 | 4.153202000000 | 16.724971000000 |
| C | 25.645986000000 | 4.505305000000 | 15.387356000000 |
| H | 25.823655000000 | 5.144816000000 | 14.499627000000 |
| H | 25.494192000000 | 3.472515000000 | 15.030800000000 |
| C | 24.185491000000 | 7.075695000000 | 17.623918000000 |
| C | 23.099683000000 | 6.786131000000 | 18.476647000000 |
| C | 22.340244000000 | 7.845262000000 | 18.988572000000 |
| H | 21.492407000000 | 7.626554000000 | 19.643154000000 |
| C | 22.663020000000 | 9.164230000000 | 18.699619000000 |
| H | 22.071378000000 | 9.981748000000 | 19.121332000000 |
| C | 23.745706000000 | 9.443456000000 | 17.867105000000 |
| H | 23.986058000000 | 10.483711000000 | 17.640891000000 |
| C | 24.509047000000 | 8.418418000000 | 17.302801000000 |
| C | 22.719146000000 | 5.361088000000 | 18.846674000000 |
| H | 23.549998000000 | 4.715256000000 | 18.539087000000 |
| C | 22.549681000000 | 5.160390000000 | 20.354231000000 |
| H | 22.409966000000 | 4.093847000000 | 20.586686000000 |
| H | 23.433657000000 | 5.514424000000 | 20.900236000000 |
| H | 21.672515000000 | 5.695703000000 | 20.750368000000 |
| C | 21.462153000000 | 4.907612000000 | 18.092665000000 |

| | | | |
|---|---|---|---|
| H | 21.229360000000 | 3.856578000000 | 18.327175000000 |
| H | 20.593144000000 | 5.520162000000 | 18.384545000000 |
| H | 21.584045000000 | 5.001717000000 | 17.002601000000 |
| C | 25.622661000000 | 8.737104000000 | 16.312381000000 |
| H | 26.462977000000 | 8.065845000000 | 16.537091000000 |
| C | 26.169111000000 | 10.162158000000 | 16.422794000000 |
| H | 26.477757000000 | 10.398469000000 | 17.453304000000 |
| H | 27.050314000000 | 10.273301000000 | 15.771099000000 |
| H | 25.432055000000 | 10.916491000000 | 16.102850000000 |
| C | 25.153835000000 | 8.447234000000 | 14.879772000000 |
| H | 24.817574000000 | 7.406089000000 | 14.774519000000 |
| H | 24.312570000000 | 9.105461000000 | 14.607063000000 |
| H | 25.969283000000 | 8.616878000000 | 14.157850000000 |
| C | 28.054733000000 | 4.147932000000 | 15.834918000000 |
| C | 28.420984000000 | 2.791974000000 | 15.979191000000 |
| C | 29.684464000000 | 2.385068000000 | 15.540055000000 |
| H | 29.981156000000 | 1.338990000000 | 15.657775000000 |
| C | 30.572369000000 | 3.291689000000 | 14.969066000000 |
| H | 31.562528000000 | 2.960632000000 | 14.642926000000 |
| C | 30.192050000000 | 4.620380000000 | 14.806356000000 |
| H | 30.886988000000 | 5.326186000000 | 14.342807000000 |
| C | 28.930199000000 | 5.066388000000 | 15.214032000000 |
| C | 27.478560000000 | 1.769940000000 | 16.595829000000 |
| H | 26.548808000000 | 2.299475000000 | 16.844682000000 |
| C | 28.044410000000 | 1.197102000000 | 17.898849000000 |
| H | 27.335122000000 | 0.494196000000 | 18.362805000000 |
| H | 28.986185000000 | 0.650529000000 | 17.730925000000 |
| H | 28.249958000000 | 1.998395000000 | 18.623152000000 |
| C | 27.128667000000 | 0.650232000000 | 15.607818000000 |
| H | 26.390764000000 | -0.035666000000 | 16.054674000000 |
| H | 26.704989000000 | 1.054950000000 | 14.674711000000 |
| H | 28.017296000000 | 0.054666000000 | 15.341717000000 |
| C | 28.543174000000 | 6.517636000000 | 14.964289000000 |
| H | 27.488941000000 | 6.637206000000 | 15.247871000000 |
| C | 29.367377000000 | 7.475886000000 | 15.832805000000 |
| H | 29.048222000000 | 8.518709000000 | 15.671995000000 |
| H | 29.238589000000 | 7.242129000000 | 16.900303000000 |
| H | 30.440305000000 | 7.409174000000 | 15.588003000000 |
| C | 28.645777000000 | 6.879510000000 | 13.476917000000 |
| H | 28.285837000000 | 7.907142000000 | 13.306504000000 |
| H | 29.685474000000 | 6.828854000000 | 13.116022000000 |
| H | 28.041626000000 | 6.196636000000 | 12.859040000000 |
| C | 29.501928000000 | 7.624479000000 | 23.200152000000 |
| H | 30.071195000000 | 7.741728000000 | 24.145179000000 |
| H | 29.142695000000 | 8.622522000000 | 22.900901000000 |
| C | 30.372463000000 | 6.992948000000 | 22.116611000000 |
| H | 30.014524000000 | 7.293472000000 | 21.114545000000 |
| H | 31.426458000000 | 7.296150000000 | 22.212002000000 |
| C | 27.256591000000 | 7.113848000000 | 24.142283000000 |
| C | 27.127921000000 | 6.736387000000 | 25.497445000000 |
| C | 25.996803000000 | 7.154136000000 | 26.208320000000 |
| H | 25.880002000000 | 6.854363000000 | 27.253485000000 |
| C | 25.024238000000 | 7.950317000000 | 25.613155000000 |
| H | 24.149703000000 | 8.270685000000 | 26.186571000000 |
| C | 25.170314000000 | 8.335886000000 | 24.283892000000 |
| H | 24.404406000000 | 8.960593000000 | 23.815608000000 |
| C | 26.273866000000 | 7.926113000000 | 23.530611000000 |

| | | | |
|---|---|---|---|
| C | 28.163281000000 | 5.878730000000 | 26.211255000000 |
| H | 29.013806000000 | 5.741924000000 | 25.531334000000 |
| C | 27.594111000000 | 4.488303000000 | 26.522105000000 |
| H | 28.349394000000 | 3.855912000000 | 27.014493000000 |
| H | 26.722866000000 | 4.560558000000 | 27.193850000000 |
| H | 27.276409000000 | 3.979165000000 | 25.598730000000 |
| C | 28.704110000000 | 6.562244000000 | 27.473130000000 |
| H | 29.513944000000 | 5.959696000000 | 27.916263000000 |
| H | 29.106035000000 | 7.561315000000 | 27.242126000000 |
| H | 27.923018000000 | 6.683138000000 | 28.240887000000 |
| C | 26.383783000000 | 8.360599000000 | 22.076668000000 |
| H | 27.301824000000 | 7.911851000000 | 21.673101000000 |
| C | 25.213191000000 | 7.827175000000 | 21.244814000000 |
| H | 25.336380000000 | 8.075823000000 | 20.178746000000 |
| H | 25.141519000000 | 6.733759000000 | 21.331191000000 |
| H | 24.250158000000 | 8.250253000000 | 21.572272000000 |
| C | 26.503158000000 | 9.884228000000 | 21.945919000000 |
| H | 27.352629000000 | 10.275695000000 | 22.528186000000 |
| H | 26.646427000000 | 10.168995000000 | 20.890863000000 |
| H | 25.592011000000 | 10.389179000000 | 22.306380000000 |
| C | 31.348725000000 | 4.659703000000 | 22.138436000000 |
| C | 31.991168000000 | 4.076201000000 | 23.253165000000 |
| C | 33.008140000000 | 3.141925000000 | 23.028169000000 |
| H | 33.498738000000 | 2.668180000000 | 23.883230000000 |
| C | 33.420660000000 | 2.821563000000 | 21.738630000000 |
| H | 34.222290000000 | 2.093861000000 | 21.583146000000 |
| C | 32.816280000000 | 3.441580000000 | 20.648704000000 |
| H | 33.149194000000 | 3.199768000000 | 19.635765000000 |
| C | 31.773208000000 | 4.354555000000 | 20.826318000000 |
| C | 31.635369000000 | 4.446361000000 | 24.685600000000 |
| H | 30.891367000000 | 5.253227000000 | 24.640186000000 |
| C | 31.004400000000 | 3.267841000000 | 25.435847000000 |
| H | 30.746625000000 | 3.557972000000 | 26.467443000000 |
| H | 30.083411000000 | 2.935460000000 | 24.933827000000 |
| H | 31.700174000000 | 2.414436000000 | 25.491001000000 |
| C | 32.851767000000 | 5.003601000000 | 25.436840000000 |
| H | 32.555565000000 | 5.355339000000 | 26.438680000000 |
| H | 33.633497000000 | 4.238494000000 | 25.570790000000 |
| H | 33.300597000000 | 5.850136000000 | 24.893700000000 |
| C | 31.134116000000 | 5.013844000000 | 19.615578000000 |
| H | 30.219159000000 | 5.506617000000 | 19.968319000000 |
| C | 30.716666000000 | 4.009674000000 | 18.539059000000 |
| H | 30.136844000000 | 4.509150000000 | 17.748179000000 |
| H | 31.584809000000 | 3.537130000000 | 18.053183000000 |
| H | 30.094050000000 | 3.209105000000 | 18.961263000000 |
| C | 32.050088000000 | 6.096418000000 | 19.029153000000 |
| H | 32.317416000000 | 6.852390000000 | 19.783783000000 |
| H | 32.987373000000 | 5.653573000000 | 18.653240000000 |
| H | 31.554852000000 | 6.607247000000 | 18.187379000000 |

**4**

| | | | |
|---|---|---|---|
| P | 9.585555000000 | 10.208714000000 | 26.712932000000 |
| P | 11.851864000000 | 10.892808000000 | 24.625278000000 |
| P | 11.675095000000 | 12.491427000000 | 27.342865000000 |
| P | 9.974950000000 | 8.633757000000 | 24.225622000000 |
| P | 13.887970000000 | 12.899275000000 | 25.408209000000 |

| | | | |
|---|---|---|---|
| P | 9.650238000000 | 11.611156000000 | 29.324237000000 |
| S | 8.732680000000 | 7.567670000000 | 25.277169000000 |
| S | 14.688304000000 | 11.954440000000 | 23.906285000000 |
| S | 10.874573000000 | 12.732856000000 | 30.340549000000 |
| N | 9.547639000000 | 9.208065000000 | 22.689270000000 |
| N | 11.339459000000 | 7.839693000000 | 23.617299000000 |
| N | 13.593220000000 | 14.568165000000 | 25.361544000000 |
| N | 14.857511000000 | 13.050438000000 | 26.783233000000 |
| N | 8.001498000000 | 11.986819000000 | 29.218307000000 |
| N | 9.318853000000 | 10.080860000000 | 29.964195000000 |
| C | 10.481090000000 | 10.054056000000 | 25.251001000000 |
| C | 12.320925000000 | 12.057658000000 | 25.803934000000 |
| C | 10.327301000000 | 11.460318000000 | 27.638652000000 |
| C | 10.318745000000 | 8.599589000000 | 21.606501000000 |
| H | 10.446423000000 | 9.326174000000 | 20.790274000000 |
| H | 9.805013000000 | 7.709327000000 | 21.198038000000 |
| C | 11.655759000000 | 8.227562000000 | 22.240336000000 |
| H | 12.133534000000 | 7.383423000000 | 21.723964000000 |
| H | 12.348621000000 | 9.087599000000 | 22.220397000000 |
| C | 8.397909000000 | 10.000938000000 | 22.372492000000 |
| C | 7.120241000000 | 9.406180000000 | 22.292497000000 |
| C | 6.022142000000 | 10.225537000000 | 22.007350000000 |
| H | 5.024288000000 | 9.782579000000 | 21.955245000000 |
| C | 6.182116000000 | 11.585521000000 | 21.761282000000 |
| H | 5.310891000000 | 12.208610000000 | 21.539480000000 |
| C | 7.456579000000 | 12.143360000000 | 21.768195000000 |
| H | 7.584403000000 | 13.204670000000 | 21.538042000000 |
| C | 8.580440000000 | 11.366343000000 | 22.063392000000 |
| C | 6.914937000000 | 7.902551000000 | 22.393838000000 |
| H | 7.842021000000 | 7.463787000000 | 22.779524000000 |
| C | 5.800981000000 | 7.519624000000 | 23.371937000000 |
| H | 5.772075000000 | 6.425960000000 | 23.502057000000 |
| H | 5.979461000000 | 7.969548000000 | 24.359238000000 |
| H | 4.809672000000 | 7.838780000000 | 23.010216000000 |
| C | 6.657913000000 | 7.315145000000 | 20.998151000000 |
| H | 6.581102000000 | 6.216576000000 | 21.050188000000 |
| H | 5.718210000000 | 7.700863000000 | 20.569301000000 |
| H | 7.470514000000 | 7.569572000000 | 20.299301000000 |
| C | 9.960908000000 | 11.995583000000 | 21.974419000000 |
| H | 10.689367000000 | 11.234753000000 | 22.285853000000 |
| C | 10.115871000000 | 13.196033000000 | 22.909098000000 |
| H | 11.143154000000 | 13.588235000000 | 22.871573000000 |
| H | 9.439154000000 | 14.018893000000 | 22.632369000000 |
| H | 9.894334000000 | 12.923016000000 | 23.949877000000 |
| C | 10.296203000000 | 12.390613000000 | 20.529453000000 |
| H | 10.204735000000 | 11.534746000000 | 19.841936000000 |
| H | 9.620205000000 | 13.182000000000 | 20.166562000000 |
| H | 11.327018000000 | 12.775770000000 | 20.469009000000 |
| C | 11.961320000000 | 6.698766000000 | 24.224349000000 |
| C | 11.327826000000 | 5.438816000000 | 24.149419000000 |
| C | 11.920190000000 | 4.359982000000 | 24.814422000000 |
| H | 11.436754000000 | 3.380592000000 | 24.785259000000 |
| C | 13.125991000000 | 4.507645000000 | 25.493234000000 |
| H | 13.570570000000 | 3.652461000000 | 26.010576000000 |
| C | 13.782214000000 | 5.734535000000 | 25.483166000000 |
| H | 14.750097000000 | 5.834931000000 | 25.979594000000 |
| C | 13.220269000000 | 6.844355000000 | 24.845444000000 |

| | | | |
|---|---|---|---|
| C | 10.106862000000 | 5.194689000000 | 23.273319000000 |
| H | 9.637597000000 | 6.164850000000 | 23.075681000000 |
| C | 9.044844000000 | 4.312567000000 | 23.933661000000 |
| H | 8.137320000000 | 4.285350000000 | 23.308685000000 |
| H | 9.390294000000 | 3.273006000000 | 24.054177000000 |
| H | 8.766835000000 | 4.709826000000 | 24.919882000000 |
| C | 10.548549000000 | 4.604185000000 | 21.925118000000 |
| H | 11.281810000000 | 5.251775000000 | 21.419602000000 |
| H | 11.019631000000 | 3.617075000000 | 22.062612000000 |
| H | 9.682437000000 | 4.478253000000 | 21.254511000000 |
| C | 14.010066000000 | 8.138485000000 | 24.746026000000 |
| H | 13.288649000000 | 8.943310000000 | 24.547181000000 |
| C | 14.785763000000 | 8.505321000000 | 26.012630000000 |
| H | 15.167621000000 | 9.534345000000 | 25.932360000000 |
| H | 14.158400000000 | 8.436910000000 | 26.911346000000 |
| H | 15.657411000000 | 7.847816000000 | 26.161594000000 |
| C | 14.966057000000 | 8.083254000000 | 23.544900000000 |
| H | 15.479159000000 | 9.050399000000 | 23.427361000000 |
| H | 15.726677000000 | 7.299330000000 | 23.696934000000 |
| H | 14.436874000000 | 7.855243000000 | 22.607791000000 |
| C | 14.468231000000 | 15.331344000000 | 26.251569000000 |
| H | 15.387465000000 | 15.656740000000 | 25.728944000000 |
| H | 13.939976000000 | 16.228577000000 | 26.605771000000 |
| C | 14.790182000000 | 14.376787000000 | 27.397633000000 |
| H | 14.003479000000 | 14.417012000000 | 28.173424000000 |
| H | 15.754458000000 | 14.610769000000 | 27.870023000000 |
| C | 12.782443000000 | 15.270972000000 | 24.412487000000 |
| C | 11.536638000000 | 15.786548000000 | 24.834104000000 |
| C | 10.746978000000 | 16.474378000000 | 23.908438000000 |
| H | 9.777002000000 | 16.869767000000 | 24.222022000000 |
| C | 11.177119000000 | 16.663062000000 | 22.598898000000 |
| H | 10.543048000000 | 17.194191000000 | 21.883131000000 |
| C | 12.428893000000 | 16.197435000000 | 22.210488000000 |
| H | 12.781253000000 | 16.383926000000 | 21.192688000000 |
| C | 13.260837000000 | 15.517683000000 | 23.107908000000 |
| C | 11.056611000000 | 15.671983000000 | 26.271553000000 |
| H | 11.782742000000 | 15.049669000000 | 26.811600000000 |
| C | 9.689641000000 | 14.991068000000 | 26.374673000000 |
| H | 9.685851000000 | 14.017830000000 | 25.864410000000 |
| H | 9.414664000000 | 14.824488000000 | 27.427412000000 |
| H | 8.895026000000 | 15.602598000000 | 25.920413000000 |
| C | 11.026812000000 | 17.045136000000 | 26.957274000000 |
| H | 12.006844000000 | 17.545830000000 | 26.909327000000 |
| H | 10.290104000000 | 17.711458000000 | 26.479665000000 |
| H | 10.744955000000 | 16.936464000000 | 28.017120000000 |
| C | 14.675147000000 | 15.162818000000 | 22.676394000000 |
| H | 15.113949000000 | 14.526098000000 | 23.453083000000 |
| C | 14.706518000000 | 14.359557000000 | 21.372956000000 |
| H | 15.735043000000 | 14.029825000000 | 21.155786000000 |
| H | 14.078493000000 | 13.460715000000 | 21.456771000000 |
| H | 14.358155000000 | 14.957464000000 | 20.514679000000 |
| C | 15.528566000000 | 16.435054000000 | 22.571056000000 |
| H | 16.572026000000 | 16.179139000000 | 22.324227000000 |
| H | 15.150089000000 | 17.108372000000 | 21.784279000000 |
| H | 15.528379000000 | 16.997714000000 | 23.518291000000 |
| C | 15.853219000000 | 12.099968000000 | 27.181742000000 |
| C | 15.606583000000 | 11.268130000000 | 28.290724000000 |

| | | | |
|---|---|---|---|
| C | 16.559179000000 | 10.299267000000 | 28.625720000000 |
| H | 16.371579000000 | 9.635379000000 | 29.473927000000 |
| C | 17.735818000000 | 10.173127000000 | 27.897044000000 |
| H | 18.465498000000 | 9.402242000000 | 28.161256000000 |
| C | 18.001292000000 | 11.054069000000 | 26.850960000000 |
| H | 18.951284000000 | 10.981252000000 | 26.316198000000 |
| C | 17.083383000000 | 12.043222000000 | 26.487243000000 |
| C | 14.373722000000 | 11.427385000000 | 29.165082000000 |
| H | 13.777978000000 | 12.254534000000 | 28.756022000000 |
| C | 13.489210000000 | 10.181497000000 | 29.157488000000 |
| H | 12.613220000000 | 10.335752000000 | 29.802364000000 |
| H | 13.135636000000 | 9.960229000000 | 28.140589000000 |
| H | 14.025252000000 | 9.292852000000 | 29.526806000000 |
| C | 14.753480000000 | 11.822071000000 | 30.597892000000 |
| H | 13.840845000000 | 12.012338000000 | 31.184286000000 |
| H | 15.319119000000 | 11.020194000000 | 31.099949000000 |
| H | 15.374502000000 | 12.732176000000 | 30.611393000000 |
| C | 17.482779000000 | 13.089761000000 | 25.456060000000 |
| H | 16.567592000000 | 13.583861000000 | 25.109403000000 |
| C | 18.166145000000 | 12.498219000000 | 24.220762000000 |
| H | 18.308336000000 | 13.281028000000 | 23.458015000000 |
| H | 19.161199000000 | 12.088397000000 | 24.458404000000 |
| H | 17.551486000000 | 11.700917000000 | 23.780111000000 |
| C | 18.373479000000 | 14.153549000000 | 26.116586000000 |
| H | 17.877420000000 | 14.618439000000 | 26.982952000000 |
| H | 19.316417000000 | 13.709468000000 | 26.475600000000 |
| H | 18.624710000000 | 14.950449000000 | 25.397263000000 |
| C | 7.148254000000 | 11.041606000000 | 29.937963000000 |
| H | 6.171594000000 | 10.971576000000 | 29.436964000000 |
| H | 6.977958000000 | 11.367521000000 | 30.981407000000 |
| C | 7.903369000000 | 9.716769000000 | 29.888959000000 |
| H | 7.647164000000 | 9.065414000000 | 30.736344000000 |
| H | 7.680009000000 | 9.173544000000 | 28.952380000000 |
| C | 7.439825000000 | 13.210440000000 | 28.729005000000 |
| C | 6.803889000000 | 13.206594000000 | 27.468067000000 |
| C | 6.269422000000 | 14.403267000000 | 26.982297000000 |
| H | 5.784760000000 | 14.414412000000 | 26.002230000000 |
| C | 6.341914000000 | 15.575132000000 | 27.728832000000 |
| H | 5.928112000000 | 16.506360000000 | 27.331171000000 |
| C | 6.916000000000 | 15.552006000000 | 28.995620000000 |
| H | 6.930951000000 | 16.464748000000 | 29.596827000000 |
| C | 7.453027000000 | 14.373969000000 | 29.527510000000 |
| C | 6.621426000000 | 11.931959000000 | 26.661305000000 |
| H | 7.170291000000 | 11.134801000000 | 27.180236000000 |
| C | 7.195652000000 | 12.047739000000 | 25.248278000000 |
| H | 7.144093000000 | 11.080080000000 | 24.726011000000 |
| H | 6.640989000000 | 12.778968000000 | 24.640508000000 |
| H | 8.246435000000 | 12.368167000000 | 25.269469000000 |
| C | 5.143847000000 | 11.518447000000 | 26.611638000000 |
| H | 5.034320000000 | 10.555455000000 | 26.087186000000 |
| H | 4.716376000000 | 11.413716000000 | 27.621444000000 |
| H | 4.540313000000 | 12.266541000000 | 26.072151000000 |
| C | 7.930645000000 | 14.367659000000 | 30.971427000000 |
| H | 8.446942000000 | 13.417544000000 | 31.150287000000 |
| C | 8.937746000000 | 15.483658000000 | 31.263839000000 |
| H | 9.345483000000 | 15.368802000000 | 32.280828000000 |
| H | 9.780597000000 | 15.440206000000 | 30.558917000000 |

| | | | |
|---|---|---|---|
| H | 8.473034000000 | 16.481409000000 | 31.200941000000 |
| C | 6.726465000000 | 14.439511000000 | 31.921878000000 |
| H | 7.058110000000 | 14.366877000000 | 32.970711000000 |
| H | 6.180740000000 | 15.390283000000 | 31.804269000000 |
| H | 6.013719000000 | 13.621739000000 | 31.729747000000 |
| C | 10.248722000000 | 9.297531000000 | 30.723034000000 |
| C | 10.824677000000 | 8.148865000000 | 30.143027000000 |
| C | 11.786719000000 | 7.443546000000 | 30.873766000000 |
| H | 12.249221000000 | 6.556431000000 | 30.433280000000 |
| C | 12.159431000000 | 7.853485000000 | 32.148826000000 |
| H | 12.926662000000 | 7.302361000000 | 32.700362000000 |
| C | 11.524137000000 | 8.942661000000 | 32.740003000000 |
| H | 11.781785000000 | 9.223653000000 | 33.763877000000 |
| C | 10.542509000000 | 9.665597000000 | 32.055759000000 |
| C | 10.360123000000 | 7.598979000000 | 28.803925000000 |
| H | 9.699999000000 | 8.347644000000 | 28.346113000000 |
| C | 11.497924000000 | 7.329543000000 | 27.818404000000 |
| H | 12.024226000000 | 8.257642000000 | 27.555794000000 |
| H | 12.242084000000 | 6.624785000000 | 28.221332000000 |
| H | 11.097585000000 | 6.894965000000 | 26.891981000000 |
| C | 9.514351000000 | 6.335705000000 | 29.018189000000 |
| H | 8.674701000000 | 6.526443000000 | 29.705718000000 |
| H | 9.105995000000 | 5.989470000000 | 28.055624000000 |
| H | 10.120983000000 | 5.521715000000 | 29.448538000000 |
| C | 9.736503000000 | 10.720546000000 | 32.801133000000 |
| H | 9.247116000000 | 11.357192000000 | 32.055334000000 |
| C | 10.590737000000 | 11.637504000000 | 33.679582000000 |
| H | 9.972087000000 | 12.456753000000 | 34.080298000000 |
| H | 11.020163000000 | 11.100199000000 | 34.540656000000 |
| H | 11.409170000000 | 12.083150000000 | 33.096870000000 |
| C | 8.638549000000 | 10.034650000000 | 33.629149000000 |
| H | 7.988986000000 | 9.407096000000 | 32.999060000000 |
| H | 9.077690000000 | 9.383187000000 | 34.402487000000 |
| H | 8.006380000000 | 10.783949000000 | 34.133740000000 |

## 5. References


1 SAINT V8.41 (2024), Bruker AXS, Madison, WI, USA.

2 L. Krause, R. Herbst-Irmer and G. M. Sheldrick, D. Stalke, "Comparison of Ag and Mo microfocus X-ray sources for single-crystal structure determination" *J. Appl. Cryst.*, 2015, **48**, 3–10.

3 G. M. Sheldrick, "SHELXT–Integrated space-group and crystal-structure determination" *Acta Cryst.*, 2015, **A71**, 3–8.

4 G. M. Sheldrick, "Crystal structure refinement with SHELXL" *Acta Cryst.*, 2015, **C71**, 3–8.

5 A. L. Spek, "PLATON SQUEEZE: a tool for the calculation of the disordered solvent contribution to the calculated structure factors" *Acta Cryst.*, 2015, **C71**, 9–18.

6 A. Immirzi and B. Perini, "Prediction of density in organic crystals" *Acta Cryst.* 1977, **A33**, 216–218.

7 F. Neese, "Software Update: The ORCA Program System—Version 6.0" *WIREs Comput Mol Sci.*, 2025, **15**, e70019.

8 A. Najibi and L. Goerigk. "The Nonlocal Kernel in van der Waals Density Functionals as an Additive Correction: An Extensive Analysis with Special Emphasis on the B97M-V and ωB97M-V Approaches" *J. Chem. Theory Comput.*, 2018, **14**, 5725–5738.

9 F. Weigend and R. Alhrichs, "Balanced basis sets of split valence, triple zeta valence and quadruple zeta valence quality for H to Rn: Design and assessment of accuracy" *Phys. Chem. Chem. Phy*s., 2005, **7**, 3297–3305.

10 B. Helmich-Paris, B. de Souza, F. Neese and R. Izsák. "An improved chain of spheres for exchange algorithm" *J. Chem. Phys.*, 2021, **155**, 104109–104122.

11 Y. Zhao and D. G. Truhlar, "The M06 suite of density functionals for main group thermochemistry, thermochemical kinetics, noncovalent interactions, excited states, and transition elements: two new functionals and systematic testing of four M06-class functionals and 12 other functionals" *Theor. Chem. Acc.*, 2008, **120***,* 215–241.

12 V. Barone and M. Cossi, "Quantum Calculation of Molecular Energies and Energy Gradients in Solution by a Conductor Solvent Model" *J. Phys. Chem. A*, 1998, 102, 1995–2001.
